# Sibanye Methods for Prevention Packages Program Project Protocol: Pilot Study of HIV Prevention Interventions for Men Who Have Sex With Men in South Africa

**DOI:** 10.2196/resprot.3737

**Published:** 2014-10-16

**Authors:** AD McNaghten, Rachel Kearns, Aaron J Siegler, Nancy Phaswana-Mafuya, Linda-Gail Bekker, Rob Stephenson, Stefan D Baral, Ron Brookmeyer, Clarence S Yah, Andrew J Lambert, Benjamin Brown, Eli Rosenberg, Mondie Blalock Tharp, Alex de Voux, Chris Beyrer, Patrick S Sullivan

**Affiliations:** ^1^Department of EpidemiologyEmory UniversityAtlanta, GAUnited States; ^2^HIV/AIDS/STI/TB Research ProgrammeHuman Sciences Research CouncilPort ElizabethSouth Africa; ^3^Office of the Deputy Vice Chancellor: Research & EngagementNelson Mandela Metropolitan UniversityPort ElizabethSouth Africa; ^4^Desmond Tutu HIV FoundationCape TownSouth Africa; ^5^Hubert Department of Global HealthEmory UniversityAtlanta, GAUnited States; ^6^Department of EpidemiologyJohns Hopkins UniversityBaltimore, MDUnited States; ^7^Department of BiostatisticsUniversity of California, Los AngelesLos Angeles, CAUnited States; ^8^Key Populations ProgrammeTB/HIV Care AssociationCape TownSouth Africa; ^9^Center for Public Health and Human RightsJohns Hopkins UniversityBaltimore, MDUnited States

**Keywords:** HIV, prevention & control, South Africa, Truvada

## Abstract

**Background:**

Human immunodeficiency virus (HIV) prevention intervention programs and related research for men who have sex with men (MSM) in the southern African region remain limited, despite the emergence of a severe epidemic among this group. With a lack of understanding of their social and sexual lives and HIV risks, and with MSM being a hidden and stigmatized group in the region, optimized HIV prevention packages for southern African MSM are an urgent public health and research priority.

**Objective:**

The objective of the Sibanye Health Project is to develop and evaluate a combination package of biomedical, behavioral, and community-level HIV prevention interventions and services for MSM in South Africa.

**Methods:**

The project consists of three phases: (1) a comprehensive literature review and summary of current HIV prevention interventions (Phase I), (2) agent-based mathematical modeling of HIV transmission in southern African MSM (Phase II), and (3) formative and stigma-related qualitative research, community engagement, training on providing health care to MSM, and the pilot study (Phase III). The pilot study is a prospective one-year study of 200 men in Cape Town and Port Elizabeth, South Africa. The study will assess a package of HIV prevention services, including condom and condom-compatible lubricant choices, risk-reduction counseling, couples HIV testing and counseling, pre-exposure prophylaxis (PrEP) for eligible men, and non-occupational post-exposure prophylaxis for men with a high risk exposure. The pilot study will begin in October 2014.

**Results:**

Preliminary results from all components but the pilot study are available. We developed a literature review database with meta-data extracted from 3800 documents from 67 countries. Modeling results indicate that regular HIV testing and promotion of condom use can significantly impact new HIV infections among South African MSM, even in the context of high coverage of early treatment of HIV-positive men and high coverage of PrEP for at-risk HIV-negative men. Formative qualitative research consisted of 79 in-depth interviews, and six focus group discussions in Cape Town and Port Elizabeth. Analysis of these data has informed pilot study protocol development and has been documented in peer-reviewed manuscripts. Qualitative work regarding stigma faced by South African MSM resulted in finalized scales for use in the pilot study questionnaire. A total of 37 health care providers completed training designed to facilitate clinically and culturally competent care for MSM in the Eastern Cape.

**Conclusions:**

The design of a future, larger study of the HIV prevention package will be conducted at the end of the pilot study, powered to detect efficacy of the prevention package. Data from the updated mathematical model, results of the pilot study, acceptability data, and advancements in HIV prevention sciences will be considered in developing the final proposed package and study design.

**Trial Registration:**

ClinicalTrials.gov NCT02043015; http://clinicaltrials.gov/show/NCT02043015 (Archived by WebCite at http://www.webcitation.org/6THvp7rAj).

## Introduction

Human immunodeficiency virus (HIV) prevention intervention programs for, and research on, men who have sex with men (MSM) in the southern African region remain limited, despite the emergence of a severe epidemic among this group [1]. Visible communities of MSM continue to emerge in southern Africa, including in countries with very high generalized HIV epidemics. The understanding of these men, their social and sexual lives, and the multiple vulnerabilities and risks associated with their sexual behaviors in the African context is still in the early stages. The very existence of MSM and their networks remains a culturally sensitive issue in several sub-Saharan African countries, and same-sex behavior is illegal in most African nations [2]. Consequently, sub-Saharan African MSM remain among the most hidden and stigmatized groups at risk in the global HIV epidemic, and a group for whom optimized HIV prevention packages, appropriate for challenging local environments, are an urgent public health and research priority.

It is clear that, at present, no single biomedical or behavioral HIV prevention intervention is sufficiently effective to change the epidemic dynamics within specific risk communities or the broader population [3], and this is particularly true for the complex social and structural contexts of MSM in southern Africa. Combined biomedical and behavioral approaches offer prospects both for reducing individual behavioral HIV acquisition risks and for lowering transmission rates within communities [4,5]. Furthermore, the social and cultural stigmas surrounding same-sex behavior in southern Africa limit the safe spaces in which men can disclose their sexual behavior or seek clinically and culturally competent sexual health and HIV prevention services, reducing the reach and impact of many individual-level HIV prevention interventions.

This protocol describes a multiphase Methods for Prevention Packages Program (MP3) project. MP3s are multidisciplinary research programs funded by the National Institutes of Health that devise combination HIV prevention packages for specific populations, examine the safety and efficacy of such approaches in the target population, and conduct pilot activities to demonstrate acceptability to the target population and appropriateness and feasibility of the study design [6]. The outcomes of the MP3 are intended to inform the design of larger prevention trials powered to determine the efficacy of an intervention package. The organizations participating in the MP3 study outlined in this protocol are Emory University, Atlanta, Georgia; Johns Hopkins Bloomberg School of Public Health, Baltimore, Maryland; University of California, Los Angeles, Los Angeles, California; Desmond Tutu HIV Foundation (DTHF), Cape Town, South Africa; and Human Sciences Research Council (HSRC), Port Elizabeth, South Africa. The objective of the Sibanye (meaning “we are one” in Xhosa) Health Project is to develop and establish the feasibility of a combination package of biomedical, behavioral, and community-level HIV prevention interventions and services for MSM in South Africa, and to establish a knowledge base to inform future appropriately scaled combination prevention research and programmatic efforts.

## Methods

### Overview

**Figure 1:**
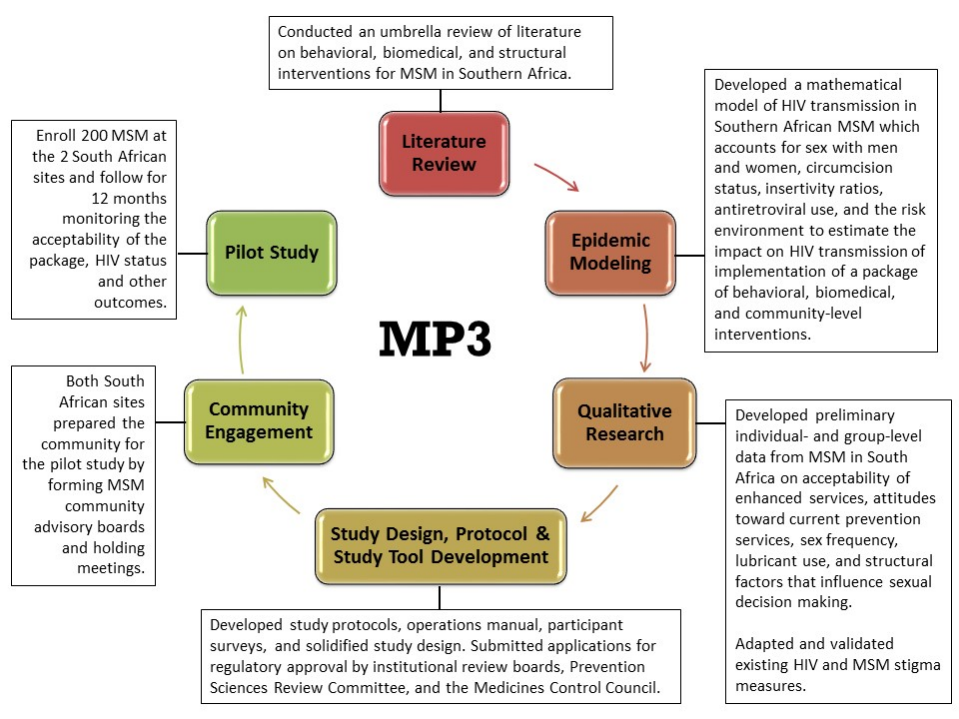
Study overview.

The study is being conducted in three phases (Figure 1). In Phase I of the project, a comprehensive literature review and summary of current knowledge of HIV prevention interventions was conducted resulting in the development of a database, providing inputs for the agent-based mathematical modeling effort (Phase II) and informing the components of the prevention package for the pilot study (Phase III). In Phase II, agent-based computer simulation models of HIV transmission in Southern African MSM were developed to estimate the impact that variations on a combination HIV prevention package might have on HIV transmission among MSM in southern Africa. Modeling outcomes were used to inform the components of the prevention package for the pilot study. Phase III includes both qualitative studies and a pilot study of the acceptability of the prevention package. The formative qualitative study incorporated in-depth interviews and focus groups to gain an understanding of the acceptability of intervention components and to determine optimal methods of intervention delivery. Community development, provider training, and stigma assessment were also conducted in preparation for the pilot study. The pilot study (to begin October 2014) will establish feasibility of cohort recruitment, retention, and prospective data collection of behavioral and biological outcomes in this study setting and will provide the basis for the design of a larger prevention trial powered to demonstrate the efficacy of the combined prevention package developed through the MP3 process. Institutional review board approval was obtained by Emory University, DTHF, and HSRC and will be obtained from the National Health Laboratory Service prior to implementation of study activities. Approval from the National Institute of Allergy and Infectious Disease, Division of AIDS, Prevention Sciences Review Committee was also obtained prior to implementation.

### Phase I: Literature Review

The goal of the literature review was to create a living database that could guide the development of Sibanye, and also be a public resource to other community members, researchers, and policy makers. This involved an umbrella review of HIV prevention interventions for MSM globally. There is consensus that best practices and experience from community organizations should inform development of guidelines. There is also a need to encourage dialogue between community members, program designers, and researchers to ensure that lessons learned are shared across countries and regions. Many of the strongest examples of community best practices supporting the needs of MSM in low and middle income countries remain undocumented in the sphere of peer-reviewed literature; however, these best practices are often documented in digital program reports for funders.

Therefore, we relied on several different methods to include peer-reviewed and grey literature implementation data instead of relying on a traditional systematic review. An electronic global consultation was completed in October 2011. Letters requesting information on epidemiology, rights contexts, and programming for MSM were sent out through HIV- and MSM-focused listservs in Asia, Africa, Latin America and the Caribbean, and Eastern Europe. These letters were also sent out by key funders of related initiatives including amfAR with its MSM Initiative to its grantees, the MSM Global Forum for HIV, and key United Nations agencies including the United Nations Development Programme and the Joint United Nations Programme on HIV/AIDS (UNAIDS). In addition, key informants were contacted in 28 countries requesting information specific to their country. A PubMed search (Multimedia Appendix 1) was completed to find additional peer-reviewed literature. To attain implementation data from larger implementers, Google and Bing, and the websites of large international HIV prevention implementers known to provide services for most-at-risk populations were searched with the same keywords used for the systematic searches.

**Figure 2:**
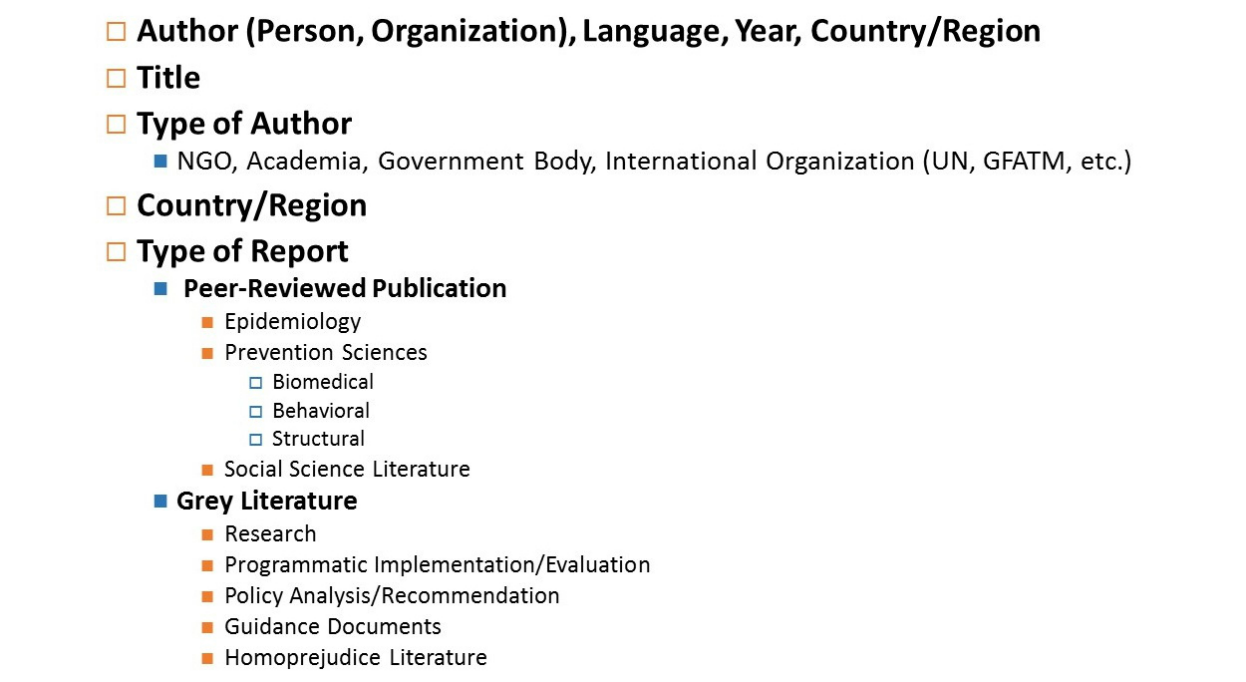
Meta-data elements extracted for materials identified in umbrella review of relevant literature, 2011-2013.

We developed a database to extract the meta-data listed in Figure 2. Data extraction included author, language, title, type of author, region of the publication, and type of report including peer-reviewed studies, HIV prevention sciences work categorized by biomedical, behavioral, structural approaches, and also non-peer-reviewed literature categorized by research, program implementation, policy analysis, guidance documents from normative agencies, and literature focused on stigma, homophobia, or homoprejudice. For documents that were freely available (ie, not peer-reviewed literature), portable document format files (PDFs) or links to the documents were included. For the remaining documents, corresponding journals were contacted to obtain rights agreements.

### Phase II: Modeling

Agent-based computer simulation modeling was conducted to develop plausible assumptions and input parameters, inform assessment of key parameters to measure for the pilot study, and explore how different combinations of interventions to increase desired prevention outcomes could influence HIV transmission at the population level. An agent-based model was developed to simulate the impact of various combinations of HIV prevention packages. The model had a number of features including accounting for sexual networks of main, regular, and casual partners among MSM. The model used South African MSM parameters to the extent they were available based on the aforementioned literature review, and later supplemented these with qualitative data collected from the formative assessment. The endpoint was the percentage of infections that could be prevented over 5 years under a range of prevention combination scenarios [7].

HIV prevention packages that involved influencing four key prevention outcomes were examined. The four components were (1) reducing unprotected anal intercourse, (2) increasing antiretroviral therapy (ART) coverage among eligible HIV-infected persons (CD4 <350 and receipt of an HIV test), (3) Truvada-based oral pre-exposure prophylaxis (PrEP) for MSM at highest risk defined as >10 partners in 6 months or persons in sero-discordant couples, and (4) reducing the proportion of the MSM population who have never been tested for HIV by 50%.

### Phase III

#### Formative Qualitative Assessment

##### Overview

To inform development of the pilot study, a series of focus group discussions and in-depth interviews was conducted with participants recruited from sites in Port Elizabeth and Cape Town, South Africa. Both qualitative methodologies were infused with a participatory learning and action approach that employed visual aids to provide structure and active participation to conversation. Detailed methods have been described previously [8]. Local languages were used in the conduct of the qualitative data collection whenever necessary.

##### Focus Group Discussions With MSM and Health Care Providers

Separate focus group discussions were conducted with health care providers and MSM, using semistructured interview guides (Multimedia Appendices 2 and 3). Men in the community were recruited using pre-existing contact lists of MSM interested in participating in research projects gathered by DTHF and HSRC. Once these men were screened and enrolled for a focus group discussion, further participants were recruited using snowball sampling from this initial group. Men were screened by telephone and given a brief description of the study procedures and aims. Men who were interested in participating were scheduled for a focus group discussion at one of several private locations at local venues and community-based organizations most convenient to participants. Men were selected to represent MSM from urban and peri-urban areas in Cape Town and Port Elizabeth. Eligible participants were male at birth, reported anal sex with a man in the past 6 months, were aged 18 years or older, and spoke English, Afrikaans, or Xhosa. Health care providers were recruited for focus group discussions from local clinics and health organizations that served the communities from which MSM were recruited. All health care staff who provided any HIV counseling, testing, or treatment services were invited to participate in the focus group discussions. The primary purpose of the focus group discussions was to explore health care service experiences and priorities in clinical settings from the perspectives of both providers and MSM. Topics included clinic-based experiences with HIV prevention services for MSM, barriers to accessing or providing services for MSM, and identification of highest priority HIV prevention services for MSM. In each group, participants were shown a list of prevention options (including condoms, lubricants, and PrEP) and were asked to rank the importance of those options for MSM. For provider focus group discussions, we also assessed experience with lesbian, gay, bisexual, and transgender (LGBT) training, interest in future training opportunities, and potential formats of provider training.

##### Interviews With MSM

In-depth interviews were held to inform continued quantitative modeling and to inform the development of the pilot intervention package. The same methods were used to recruit, screen, and enroll men for in-depth interviews as described for the focus group discussions. Face-to-face interviews were conducted at a location convenient to the participant. The interviews included a short survey (Multimedia Appendix 4) to develop parameter estimates for mathematical modeling, such as number of sexual partners in the last 6 months and frequency of sex, by sex act, over different recall periods. Each interview used a semistructured guide (Multimedia Appendix 4) anchored in participatory learning and action approaches to explore the context of HIV prevention and risk behaviors among MSM. Participants completed a timeline activity, arranging markers for topics of interest, such as history of sexual debut, safer sex behaviors, sexual identity/outness, previous relationships, HIV testing behaviors, and involvement with community organizations (see sample timeline, Multimedia Appendix 4). Active participation was encouraged by allowing participants to select markers relevant to their lives, and discussing each in the context of other life events located on the timeline. This participatory approach sought to engage participants in the discussion and to bring a visual to make concrete the discussion of past experiences. The latter part of the interview involved a network activity, where participants listed frequented locations, sexual partners in the last 6 months, close friends, and family members. Participants were then encouraged to draw connections between elements, which facilitated conversation about how each sexual partnership was related to other components of participants’ lives.

##### Analysis of Qualitative Data

The primary utility of the data analysis was to inform the packaging, content, and delivery of the combined prevention package. Analysis of transcribed data was guided by Grounded Theory, in particular drawing on constant comparison methodology [9] and using conceptual mapping to visualize relationships across data themes [10]. An inductive codebook was developed based on iterations of independent analysis among three coders followed by consensus revisions. Data management and analysis were conducted using MAXQDA software version 10.

#### Community Development and Training

DTHF has extensive experience working with MSM in the Cape Town community and has convened community advisory boards involving MSM to obtain community input on projects serving MSM. As part of community engagement activities in Port Elizabeth, the HSRC community team established a community consultation group (CCG) in July 2012 following the *Good Participatory Practice Guidelines* from UNAIDS and the AIDS Vaccine Advocacy Coalition (AVAC) [11]. This group initially consisted of key MSM stakeholder organizations such as community service, education and advocacy groups, public health representatives, and public and private sector health providers, but later became a more focused MSM CCG consisting entirely of MSM community representatives to maximize the voices of the MSM community at large. The group has more than 20 members, with approximately 10-15 members attending twice-monthly meetings to discuss a variety of health and human rights topics and receive updates on the upcoming study. The frequency of meetings has increased as the pilot study initiation nears. In essence, not only has the CCG evolved to better tailor and provide MSM community input into the study, but the CCG has become a “safe space” where MSM have been able to collectively come together from many walks of life to feel safe to discuss and collaborate on an array of topics.

#### Provider Training

Faculty from the Fenway Institute in Boston and HSRC conducted training in Port Elizabeth to increase the clinical and cultural competence of health care providers to provide care for MSM. A six-module training program was used to support these aims based on the *Guide to Lesbian, Gay, Bisexual and Transgender Health* developed by Fenway Health [12]. Training included information on the epidemiology of HIV and sexually transmitted infections (STIs) among MSM in sub-Saharan Africa, interacting with MSM patients including ascertaining patient sexual histories and physical sexual health examination, HIV prevention interventions for MSM, and risk reduction counseling methods. The training program consisted of didactic teaching and facilitated discussion with participants.

#### Stigma Measurement and Prospective Assessment

To explore how HIV and MSM stigmas compound to influence health seeking and health risk behaviors, the protocol calls for adaptation of validated stigma measures to the context of MSM in South Africa. To adapt stigma measures for MSM in South Africa, we conducted a brief literature review to identify scales that were validated for use in other populations and that fit well into Earnshaw’s Stigma Measurement model [13], which focuses on anticipated, internalized, and experienced stigma. An expert panel was convened to identify preferred scales and scale items (Multimedia Appendix 5) and to recommend appropriate adaptations of items or scale formats. Following this process, we conducted four focus groups, with 21 participants in total, to identify emic experiences with stigma for new item development. Focus groups included a pile-sorting activity to identify whether scale items aligned with Earnshaw’s stigma subdomains of anticipated, internalized, and experienced stigma. This was followed by cognitive interviews to explore participant comprehension of individual scale items (Multimedia Appendix 6). Results from focus group discussions and cognitive interviews were shared with the expert review panel, allowing for consultation on the optimal tailoring and adaptation of items.

The finalized set of stigma scale items is included in the baseline questionnaire for the pilot study that will be completed by an estimated 160 HIV-uninfected respondents and 40 HIV-infected respondents. Assessment of scale validity will include determination of scale reliability and scale factors and also correlational validity based on scale correlations with theoretically supported constructs.

Analysis will assess the impact of the pilot study’s provision of combination HIV prevention services on HIV and MSM stigmas. We hypothesize that for MSM enrolled in Sibanye, access to combination HIV prevention services provided in a culturally competent atmosphere will result in reductions in anticipated, internalized, and enacted MSM and HIV stigma.

#### Pilot Study

##### Overview

The pilot study will be a prospective one-year assessment of the implementation of a package of combination HIV prevention services. In addition to providing information on acceptability and uptake of the prevention package, the longitudinal study will develop capacity for conducting prospective data collection and providing prevention interventions and services. Data from the pilot study will also allow for refinement of estimates of key model parameters and support development of training curricula.

Approximately 200 MSM, 100 each in Cape Town and Port Elizabeth, will be followed for a period of 12 months. Participants will be offered prevention interventions, including condom choices with an assortment of styles, sizes, and features; condom-compatible lubricant choices, including water- and silicone-based types; couples HIV testing and counseling (CHTC) [14,15]; and PrEP for eligible men. Non-occupational post-exposure prophylaxis (nPEP) for men with an exposure at high risk for HIV transmission will be made available. Data on service utilization, condom use, HIV and STI incidence, acceptability of the prevention package, HIV-related knowledge, and other outcomes will be collected.

##### Participants and Enrollment

Men eligible to participate in the study will be aged 18 years and older, self-report anal intercourse with a man in the past year, be current residents of the study city, plan to stay in the city for the next year, be able to answer survey questions in English, Xhosa, or Afrikaans, be male sex at birth, be willing to provide contact information, and have a phone to facilitate the scheduling of study clinic visits. All study participants will provide written informed consent prior to participating in the pilot study.

The study will enroll both MSM who are living with HIV (HIV-positive) and those who are HIV-negative. Up to 20% of men followed for one year will be living with HIV. Additional HIV-positive men recruited will be enrolled for a baseline visit only and will not count towards the 100 men per site sample size. Recruitment and enrollment will be monitored in stages to ensure sufficient HIV-negative men are enrolled to adequately assess elements of the HIV prevention package. Enrollment of HIV-positive men will be monitored in steps. Once five HIV-positive men are enrolled prospectively at baseline, prospective enrollment of positives will pause until 20 HIV-negative men are enrolled. Men who seroconvert during follow-up will remain in the study for the full follow-up period. Recruitment activities will be conducted for approximately 3 months or until 100 MSM have been recruited and enrolled in each city. In both Cape Town and Port Elizabeth, MSM will be recruited from multiple areas including urban areas, peri-urban communities, and township areas in order to recruit diverse communities of MSM. We will employ different methods of recruitment, including event- and venue-based, online, participant referral, and walk-ins at study clinics. Men who meet the eligibility criteria during recruitment will be asked to provide contact information and be scheduled for an enrollment baseline visit.

##### Study Design and Procedures

**Figure 3:**
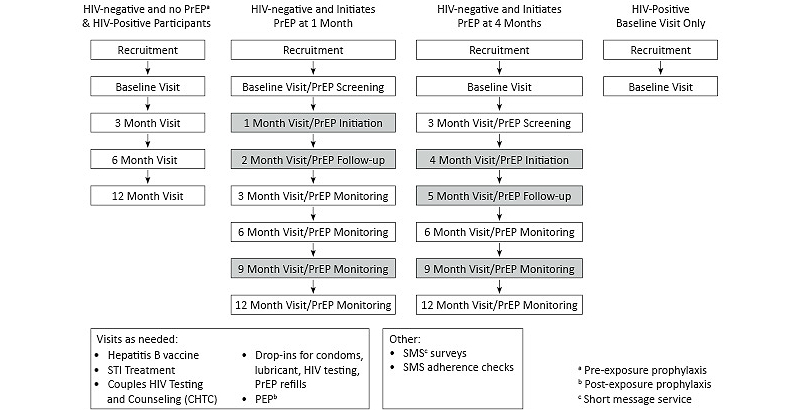
Visit schedules by participant type.

Men will be screened at recruitment, and eligible men will be invited to attend a baseline enrollment visit. Following consent and enrollment, the baseline visit will include a self-administered behavioral survey, HIV prevention counseling and testing, and a clinical exam (Figure 3). The clinical exam will assess STI history, circumcision status, STI and liver disease symptoms, and include laboratory testing for syphilis, urethral and rectal chlamydia and gonorrhea, hepatitis B, and urine drug screening. HIV-negative men will have additional testing for creatinine, liver enzymes (aspartate transaminase–aspartate aminotransferase/alanine transaminase–alanine aminotransferase [AST/ALT]), serum phosphate, proteinuria, and glycosuria to assess eligibility for PrEP. A dried blood spot specimen card will be prepared and stored. Men who present with STI symptoms will be prescribed medication at the visit. Participants can receive referrals for HIV treatment and care, circumcision, STI treatment (if outside the scope of study-provided treatment), alcohol and drug abuse counseling, and domestic violence counseling as needed. Men who test positive for STIs at baseline will return to the study clinic for treatment (if not prescribed medication during the visit). Men who are hepatitis B susceptible will be offered to initiate the hepatitis B vaccination series at their next study visit.

Follow-up study visits at 3, 6, and 12 months will include surveys, HIV prevention counseling, HIV testing for men who tested HIV-negative at their last visit, a clinical exam assessing STI symptoms, and blood and urine collection. At the 6-month and 12-month visits, all men will be tested for syphilis, and urethral and rectal chlamydia and gonorrhea. Men on PrEP will have additional monitoring at their standard and PrEP study visits and additional PrEP visits 1 month after initiating PrEP, and at 9 months to assess creatinine level, AST/ALT levels, phosphorus, proteinuria, glycosuria, HIV testing, medication adherence, and to monitor side effects. Men who test positive for HIV will have blood drawn for CD4 and HIV viral load testing. Prospectively enrolled HIV-positive participants will have additional CD4 testing at 6 and 12 months and HIV viral load testing at 3, 6, and 12 months.

##### Study Intervention

**Figure 4:**
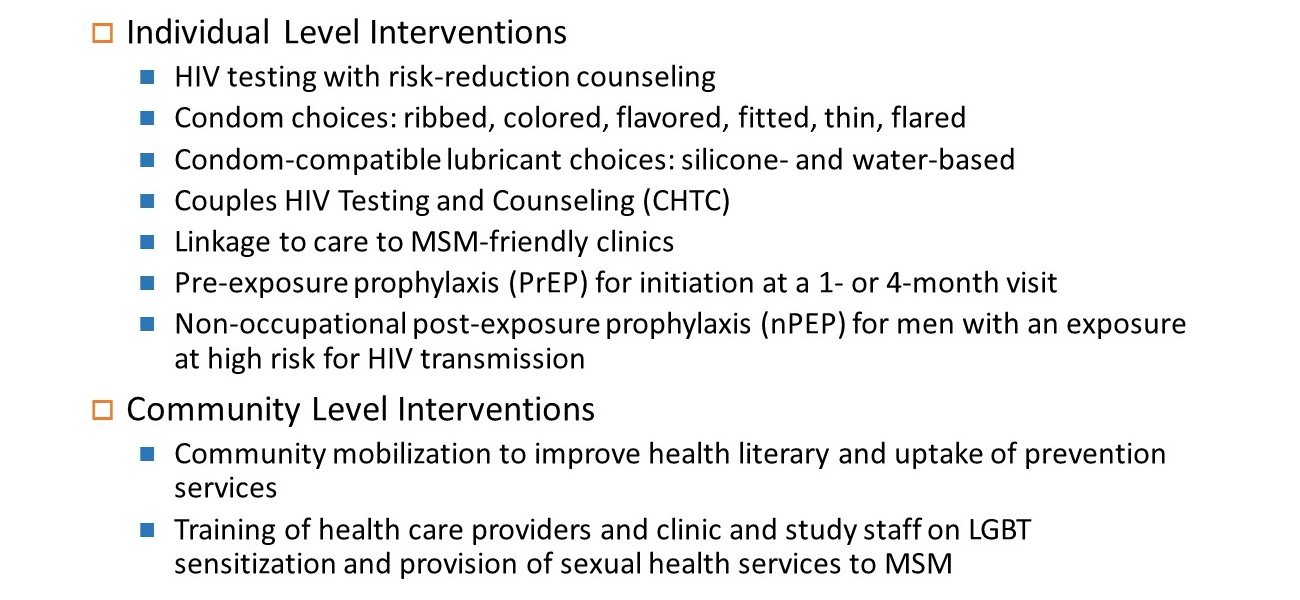
Intervention components.

The intervention package will be available starting at baseline and continuing throughout the study and will include condom and condom-compatible lubricant choices, risk reduction counseling, CHTC, linkage to care for HIV-positive men, and PrEP with emtricitabine/tenofovir disoproxil fumarate for eligible men for initiation at a 1-month or 4-month visit (Figure 4).

At their baseline visit, all participants will receive a package with condom and condom-compatible lubricant choices: ribbed, colored, flavored, fitted, thin, and flared condoms, and silicone-based and water-based lubricants. Participants will be given a Condom Scorecard to rate their condom preferences, to be returned at their next study visit (Multimedia Appendix 7). At all follow-up study visits, participants will be encouraged to pick up their preferred condom and lubricant types. Participants can also drop in to each study clinic during study clinic hours to pick up additional condoms and condom-compatible lubricant.

HIV testing will be provided to all men at their baseline visit. Risk-reduction counseling will be provided to all men regardless of their HIV status and at all standard and PrEP visits. Risk reduction counseling will be client-centered, according to the Centers for Disease Control and Prevention’s (CDC) Fundamentals of HIV Prevention Counseling curriculum. HIV testing will be provided to all men at their 3, 6, and 12-month visit who tested negative at their previous visit. HIV testing will be performed by counselors trained in using South African provincial HIV testing protocols.

Participants will be invited to schedule CHTC appointments with a clinic counselor at any point after they complete their baseline visit. The CHTC model differs from individual testing in that a couple receives HIV testing and counseling at the level of the couple, with tailored messaging based on dyadic characteristics. Participants can attend multiple CHTC sessions with different partners; these partners do not have to be enrolled in the study to participate. All CHTC sessions will be performed by counselors who completed a 3-day training curriculum on CHTC for MSM.

Men who test positive for HIV at baseline or during the study will be linked to HIV treatment and care services as needed, including antiretroviral medication and mental health services. Referrals will be made to local providers known to be MSM-friendly.

PrEP will be available for men who meet the following eligibility criteria: HIV-negative, high-risk for acquiring HIV, adequate kidney and liver functioning, initiated hepatitis B vaccine series if susceptible, not known to have hypertension or diabetes, and willing to follow PrEP dosing and visit guidelines [16]. Men who express interest and meet eligibility criteria at their baseline visit can initiate PrEP at a 1-month initiation visit. For men who do not initiate PrEP at 1 month and later express interest and meet the eligibility criteria, PrEP will be available to initiate at a 4-month initiation visit. PrEP visits will follow the guidelines determined by the Southern African HIV Clinicians Society [16]. Throughout the study, as part of the standard of care, nPEP for men with an exposure at high risk for HIV transmission will be available.

Community-level interventions include community mobilization efforts to improve health literacy and uptake of prevention services among MSM, and training of health care providers and clinic and study staff to deliver sexual health services to MSM. Community mobilization efforts began in 2012 to build the necessary capacity to conduct the pilot study, to obtain feedback on the study protocol (eg, this feedback informed our decision to include HIV positive men), and to identify and establish appropriate and accessible clinic sites in Port Elizabeth. Community leaders and members of community organizations were consulted to determine how to include HIV prevention and health education messages as part of study recruitment events. Through engagement with the community, community members have been considered and hired in study staff positions and therefore are able to play a key role in the collection, presentation, and validation of data that characterize this population. We plan to engage with the community throughout the pilot study to further create community ownership over the interventions and facilitate uptake of the interventions. The previously mentioned provider trainings will ensure that MSM in the community have access to health care from MSM-friendly clinics and providers trained in MSM-specific health issues, improving the quality of care available to MSM in the community.

##### Data Collection

Data will be collected via case report forms, self-administered surveys during study visits, and monthly short message service (SMS) surveys. Paper-based case report forms will be used to capture eligibility and enrollment data, as well as all clinical assessments, interventions, laboratory results, adverse events, and social harms reporting (Multimedia Appendix 8). All case report forms will be completed by study staff, rather than participants. These forms will be sent by scan to Emory University, with data captured using DataFax, and securely stored in participant study binders at the DTHF and HSRC offices.

At each standard study visit, participants will complete surveys on iPads or computers via SurveyGizmo, which has secure servers and a Health Insurance Portability and Accountability Act business partner agreement with Emory. Surveys will be offered in English, Xhosa, and Afrikaans (Multimedia Appendix 9). Surveys were translated from English to Xhosa and Afrikaans, then back-translated to ensure conceptual and cultural equivalence. These surveys will collect data on demographics, current use of health care services, history of HIV and STI testing, outness to health care providers, alcohol and substance abuse, history of sexual activities and condom and condom-compatible lubricant use, barriers to safer sex practices, stigma, knowledge of HIV transmission and prevention strategies, and the participant’s sexual network.

An SMS survey will be sent to consenting participants each month. These brief surveys will collect information on recent sexual activity with male and female partners, including the number of partners, frequency of sex acts, condom use, lubricant use, and HIV testing outside of the study.

##### Outcomes

**Figure 5:**
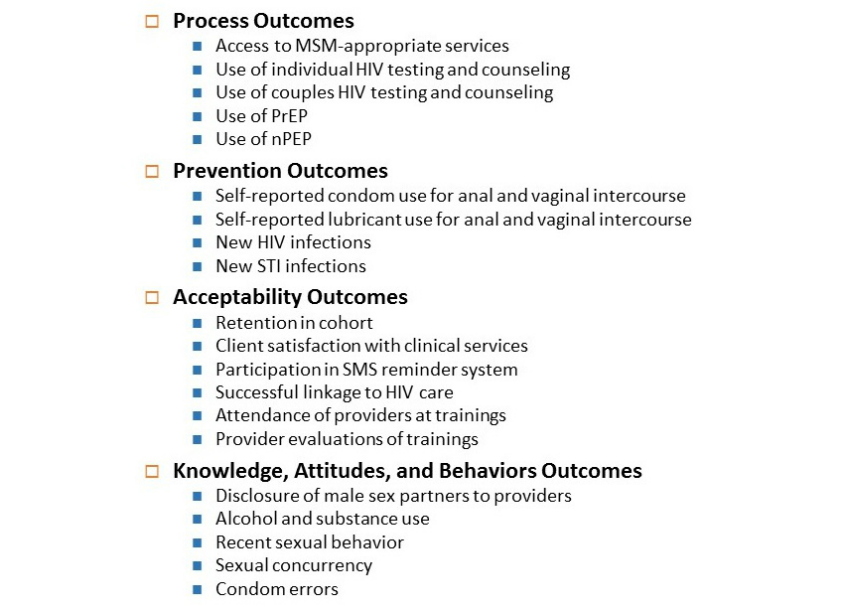
Study outcomes.

Study outcomes will be a combination of process-level outcomes, prevention impact outcomes, and acceptability and knowledge, attitudes and behaviors data, outlined in Figure 5.

##### Data Analysis Plan

The primary design for analysis will be a pre-post design. For measures that are collected from MSM participants (eg, use of condom and condom-compatible lubricant, disclosure of male sex partners to providers), data will be compared between baseline surveys and follow-up surveys. Bivariate analyses will be conducted by comparing proportions of men reporting a behavior between baseline and follow-up, using chi-square statistics. If differential retention results in follow-up data being available from a group with substantially different characteristics (eg, if older men are differentially retained, so that the 12-month participants are considerably older than the baseline sample), stratified analyses or regression modeling may be used to account for potential confounders. For biological measures, we will describe the prevalence of HIV, syphilis, chlamydia, and gonorrhea at baseline and will calculate the incidence of HIV, syphilis, chlamydia, and gonorrhea during prospective follow-up. We will conduct an intent-to-treat analysis; participants who are lost or withdraw from the study will be considered failures in analysis of uptake of the particular intervention or service of interest. HIV-positive participants who are enrolled for a baseline visit only will be included in analyses to understand prevalent behaviors and clinical features, like HIV viral load, access to care, and condom use.

##### Sample Size

Our pilot feasibility study is not powered to measure differences in pre-post measures in clinical outcomes; rather, we hope to develop reasonable estimates of the prevalence of certain behaviors at baseline and of the incidence of HIV infection and STIs, which may be important endpoints in a larger, future prevention trial. The choice of 100 participants per site is based on feasibility within time and budgetary constraints and will provide reasonable estimates of key parameters, including retention in the cohort and uptake of PrEP.

## Results

### Phase I: Literature Review

In total, 3800 documents were collected from 67 countries. Information gathered from the literature review has been made available on the Internet as a searchable electronic library. Ultimately, 2600 unique records were entered into a database, which can be viewed on the mp3docs website [17].

### Phase II: Modeling

The results of the modeling activities were presented at a plenary session at the 2013 Conference on Retroviruses and Opportunistic Infections [18], and in a published manuscript [7]. The modeling results indicate that regular HIV testing and promotion of condom use produce significant and important benefits in preventing new HIV infections among South African MSM, even in the context of high coverage of early treatment of HIV-positive men and high coverage of PrEP for at-risk HIV-negative men. With a combination package that achieves a 15% reduction in unprotected anal intercourse, some PrEP coverage, some increase in ART treatment services, and some increase in HIV testing, approximately a 35% reduction in infections can be achieved over 5 years. Modeling also found that the combined effects of the four model components to the package were not simply additive; there are some interaction effects. For example, there is an interaction between the HIV testing component and the PrEP component. If testing is increased, then PrEP as an intervention becomes more powerful because more persons become eligible for PrEP. In addition to estimating the impact of these interventions on the epidemic among South African MSM, these findings suggest that the demonstration of acceptability of a packaged prevention approach is a critical next step in optimizing prevention services for MSM in Africa.

A by-product of our modeling and simulation work was an evaluation of the sources of variation in the spread of HIV that arise from overlapping sexual networks and heterogeneity in biological and behavioral risk factors. These sources of variation are not routinely accounted for in the design of HIV prevention trials. Our work on agent-based modeling in the Sibanye MP3 project has led to useful methods for calculating required sample sizes in the design of HIV prevention trials [7].

### Phase III

#### Formative Qualitative Assessment

A qualitative assessment involving in-depth interviews and focus groups with MSM and HIV service providers was conducted in 2012 to obtain information regarding the acceptability and optimal methods of providing HIV prevention interventions for MSM.

Findings from the qualitative data collection were used to inform the components of the prevention package. Data collection for the formative assessment, including 79 in-depth interviews and surveys, six focus group discussions with health care providers and four with MSM, was completed in early 2012. Analysis of qualitative data has informed development of the pilot study interventions. For instance, in focus group discussions participants identified access to condom-compatible lubricant as a high priority service. During in-depth interviews, a number of men discussed using petroleum-based lubricant, and these men often experienced condom breakage. In part based on these findings, we decided to include in the intervention silicone-based lubricant in addition to water-based lubricant to enhance lubricant choice and fulfill the demand for lubricant provision. The analysis of qualitative data to date has also led to a manuscript regarding the contexts of condom use [8] and a manuscript regarding repeat HIV testing [19].

#### Provider Training

A total of 37 providers attended the 2-day training in Port Elizabeth, including physicians, nurses, and clinical officers from across the Eastern Cape. Participants prepared for the training by reading distributed materials in advance of participation. While the goal of the training was to increase provider skills for Sibanye, a secondary benefit was the increased clinical and cultural competence of these providers in addressing the health-related needs of MSM. Among participants who were trained and completed evaluations, many of the attendees (76%, 28/37) reported that they lacked knowledge to care for MSM prior to the training. The training was well received with 100% endorsing a statement that it is important to be sensitized to MSM needs and to learn about MSM and 100% indicating they would recommend this training to colleagues.

#### Stigma Measurement and Prospective Assessment

An expert panel came to consensus on scale preferences and revisions to individual items for a South African stigma scale for MSM. For example, to assess the anticipated MSM stigma domain of Earnshaw’s model, experts overall preferred the Liu public homosexual stigma subscale to the Pinel Stigma Consciousness Questionnaire due to use of less complex language and less focus on sexual identity. Experts also provided suggestions on individual items, including the need to allow for men of diverse sexual identities to feel included. To this end, we added an item that allows men to choose their preferred term (such as “MSM”, “gay”, or “bisexual”), which will automatically populate where relevant for all stigma items on the electronic questionnaire. Expert panel findings were compiled, resulting in a final set of scale items that were used for focus group discussions and cognitive interviews. Data from focus group discussions and cognitive interviews are currently being analyzed.

## Discussion

Preliminary results from all components but the pilot study are available, and several products have been developed through our preliminary work. A publically accessible searchable database is now available. The findings from the modeling indicating that regular HIV testing and promotion of condom use can significantly impact new HIV infections among South African MSM have been published in a peer-reviewed journal. In-depth interview and focus group discussion data were used to inform the pilot study protocol development and will soon be published. Stigma qualitative work resulted in finalized scales for use in the pilot study questionnaire. Finally, a total of 37 health care providers in the Eastern Cape completed training on LGBT sensitization and the provision of care to MSM.

Reducing new HIV transmission by expanded, early, and consistent use of ART is key to ending the HIV epidemic [20,21]. Testing, linkage to care, and treatment in South Africa falls short of that needed to significantly decrease new infections: national data from 2012 indicate that 52.6% of women and 37.5% of men had been tested and were aware of their status [22]. Although South Africa has the largest ART program in the world [23], less than 40% of ART eligible patients are on treatment [24]. MSM also face high levels of stigma and discrimination and experience barriers to accessing health care [25]. To address these issues, other MSM-targeted programs aimed at early identification of HIV infection, decreasing HIV and STI disease burden, and improving the general health and well-being of MSM are also being evaluated. A CDC-funded implementation science project focused on MSM proposes to study community- and peer-based interventions focused on finding people who are unaware of their HIV status, increasing the linkage to care and treatment initiation for those eligible, and achieving viral suppression through adherence support. This project will be conducted around the same time as Sibanye, comprehensively addressing multiple levels of HIV risk at different stages of the HIV treatment cascade for MSM in Cape Town, Port Elizabeth, and other areas across South Africa.

Although the goal of the Sibanye Health Project is to prevent HIV infection among MSM, the project activities align with other efforts in South Africa to identify and test persons at risk for HIV infection and optimize the continuum of HIV care for those living with HIV, which is a crucial component of comprehensive HIV prevention, treatment, and care programs. The Sibanye study activities allow men who test positive for HIV to be linked to MSM-friendly providers and services.

The design of a future, larger study of the HIV prevention package will be conducted at the end of the pilot study, powered to detect efficacy of the prevention package. The elements of the final proposed package will be determined by the study’s scientific advisory board and study investigators. Data from the updated mathematical model, results of pilot studies, and acceptability data will all be considered in developing the final proposed package, and the study design. We will also take into account advancements in HIV prevention sciences that may take place during our study. For example, it is likely that additional data on the real world effectiveness of PrEP as well as efficacy of intermittent PrEP will be reported during the period of our study. Depending on the specific elements that are proposed in our final prevention package, we anticipate proposing either a neighborhood- or clinic-randomized design, or an individually randomized design. This may also be influenced by whether there are more definitive data about efficacy of specific individual-level interventions including, but not limited to, circumcision, PrEP, and nPEP.

## Multimedia Appendix 1

PubMed search strategy.

## Multimedia Appendix 2

Focus group discussion guide - health care workers.

## Multimedia Appendix 3

Focus group discussion guide - men who have sex with men.

## Multimedia Appendix 4

In-depth interview guide.

## Multimedia Appendix 5

Expert review scales.

## Multimedia Appendix 6

Cognitive interview guide.

## Multimedia Appendix 7

Condom scorecard.

## Multimedia Appendix 8

Case report forms.

## Multimedia Appendix 9

Baseline survey.

## Abbreviations

ART: antiretroviral therapy
AST/ALT: aspartate transaminase–aspartate aminotransferase/alanine transaminase–alanine aminotransferase
CCG: community consultation group
CDC: Centers for Disease Control and Prevention
CHTC: couples HIV testing and counseling
DTHF: Desmond Tutu HIV Foundation
HIV: human immunodeficiency virus
HSRC: Human Sciences Research Council
LGBT: lesbian, gay, bisexual, and transgender
MSM: men who have sex with men
MP3: Methods for Prevention Packages Program
nPEP: non-occupational post-exposure prophylaxis
PrEP: pre-exposure prophylaxis
SMS: short message service
STI: sexually transmitted infection
