# Supplementary material for: Sibanye Methods for Prevention Packages Program Project Protocol: Pilot Study of HIV Prevention Interventions for Men Who Have Sex With Men in South Africa
Source: JMIR Res Protoc. 2014 Oct 16;3(4):e55. doi: 10.2196/resprot.3737 (PMC4210958; doi:10.2196/resprot.3737)
Supplement: Supplementary file 1 [file resprot_v3i4e55_app1.pdf]

Sibanye Health Project PubMed Search Strategy

HIV/AIDS

(((("HIV"[Mesh]) OR "HIV-2"[Mesh]) OR "HIV-1"[Mesh]) OR ("Acquired Immunodeficiency Syndrome/epidemiology"[Mesh] OR "Acquired Immunodeficiency Syndrome/prevention and control"[Mesh] OR "Acquired Immunodeficiency Syndrome/psychology"[Mesh] OR "Acquired Immunodeficiency Syndrome/therapy"[Mesh])) OR ("HIV Infections/epidemiology"[Mesh] OR "HIV Infections/prevention and control"[Mesh] OR "HIV Infections/psychology"[Mesh] OR "HIV Infections/therapy"[Mesh]) OR “HIV”[all] OR “HIV-1”[all] OR “HIV-2”[all] OR “Human immunodeficiency viruses”[all] OR “AIDS virus”[all] OR “AIDS viruses”[all] OR“HTLV-III”[all] OR “Human Immunodeficiency Virus”[all] OR “Human Immunodeficiency Viruses”[all] OR ”Human T Cell Lymphotropic Virus Type III”[all] OR “Human T Lymphotropic Virus Type III”[all] OR “Human T-Cell Leukemia Virus Type III”[all] OR “Human T Cell Leukemia Virus Type III”[all] OR“Human T-Cell Lymphotropic Virus Type III”[all] OR “Human T-Lymphotropic Virus Type III”[all] OR “LAV-HTLV-III”[all] OR “Lymphadenopathy-Associated Virus”[all] OR “Lymphadenopathy Associated Virus”[all] OR“Lymphadenopathy-Associated Viruses”[all] OR “Acquired Immune Deficiency Syndrome Virus”[all] OR “Acquired Immunodeficiency Syndrome Virus”[all] OR“Human Immunodeficiency Virus Type 1”[all] OR “HIV-I”[all] OR “Human immunodeficiency virus 1”[all] OR “HTLV-IV”[all] OR “HIV-II”[all] OR “Human T Lymphotropic Virus Type IV”[all] OR “Human T-Lymphotropic Virus Type IV”[all] OR “LAV-2”[all] OR “Human immunodeficiency virus 2”[all] OR “Human Immunodeficiency Virus Type 2”[all] OR “SBL-6669”[all] OR “HIV Infections”[mh] OR “Acquired Immunodeficiency Syndrome”[mh] OR “HIV Infections”[all] OR “Acquired Immunodeficiency Syndrome”[all] OR“AIDS-Associated Nephropathy”[all] OR “AIDS Dementia Complex”[all] OR“AIDS-Related Complex”[all] OR “AIDS-Related Opportunistic Infections”[all] OR“HIV-Associated Lipodystrophy Syndrome”[all] OR “HIV Enteropathy”[all] OR “HIV Seropositivity”[all] OR “HIV Wasting Syndrome”[all] OR “HIV Infection”[all] OR “HTLV-III-LAV Infections”[all] OR “HTLV III LAV Infections”[all] OR “HTLV-III-LAV Infection”[all] OR “Human T-Lymphotropic Virus Type III Infections”[all] OR“Human T Lymphotropic Virus Type III Infections”[all] OR “HTLV-III Infections”[all] OR “HTLV III Infections”[all] OR “HTLV-III Infection”[all] OR“Acquired Immunologic Deficiency Syndrome”[all] OR “Acquired Immune Deficiency Syndrome”[all] OR “Acquired Immuno-Deficiency Syndrome”[all] OR “Acquired Immuno Deficiency Syndrome”[all] OR “Acquired Immuno-Deficiency Syndromes”[all] OR “Acquired Immunodeficiency Syndromes”[all] OR “Acquired Immunodeficiency Syndrome”[all] OR “HIV prevalence” [all] AND

Intervention

("Intervention Studies"[Mesh] OR (("behaviour"[All Fields] OR "behavior"[MeSH Terms] OR "behavior"[All Fields]) AND ("Change"[Journal] OR "change"[All Fields]))) OR

(("attitude"[MeSH Terms] OR "attitude"[All Fields]) AND ("Change"[Journal] OR "change"[All Fields])) OR (("social stigma"[MeSH Terms] OR stigma[text word]) AND reduction[all fields]) OR (("discrimination (psychology)"[MeSH Terms] OR discrimination[Text Word]) AND reduction[All Fields]) OR ("evaluation studies"[Publication Type] OR "evaluation studies as topic"[MeSH Terms] OR "evaluation"[All Fields]) OR "legal literacy"[all] OR "legal education"[all]

Men who have sex with Men

“men who have sex with men”[tw] OR “MSM”[tw] OR “males who have sex with males”[tw] OR “bisexual men”[tw] OR “bisexual male”[tw] OR “bisexual males”[tw] OR “HSH”[tw] OR “Hommes ayant des rapports Sexuels avec des Hommes”[tw] OR "Homosexuality, Male"[Mesh] OR “male homosexual*”[tw] OR “gay men”[tw] OR “gay man”[tw] OR “gay male*”[tw] OR “homosexual male*”[tw] OR “homosexual males”[tw] OR “homosexual man”[tw] OR “homosexual men” OR “sex for money”[tw]
