# Supplementary material for: Sibanye Methods for Prevention Packages Program Project Protocol: Pilot Study of HIV Prevention Interventions for Men Who Have Sex With Men in South Africa
Source: JMIR Res Protoc. 2014 Oct 16;3(4):e55. doi: 10.2196/resprot.3737 (PMC4210958; doi:10.2196/resprot.3737)
Supplement: Supplementary file 2 [file resprot_v3i4e55_app2.pdf]

**Human Sciences Research Council & Emory University Rollins School of Public Health**
**Methods for Prevention Packages Program (MP3)**

# Focus Group Discussion Question Guide:
***HIV Service Providers***

*Introduction:*
Hi, my name is _____________ and I want to thank you for joining us today. I am helping to coordinate this study on the health and HIV prevention strategies of local men who have sex with men here in Cape Town, South Africa. We are conducting this research to identify your views about the best ways of delivering HIV care. Your input is very important to this research, and we anticipate that your participation will help us streamline the HIV prevention services for MSM. Today, we will simply be discussing your views, opinions and experiences on a range of topics, so please feel comfortable to say what you honestly feel. There are no right or wrong answers in our discussion. As you read on the consent form, I would like to tape record today's session. Please do not be concerned about this: we take confidentiality of your responses very seriously, and you can trust that the information you tell us will ONLY be used for this research project. As we are tape recording the interview, we ask that you refrain from using names, though if you do, we will strike them from the record. If at any time during the interview you feel uncomfortable you can ask for a break, refuse to answer any question, and are always free to leave. Do you have any questions before we start? Great. Let's begin.

*Question Guide:*

**1.** Please name the health care facilities where you are employed.
1a. What areas of town do your patients come from?
1b. Please describe the populations you serve (elderly, children, couples, etc.). Could you break them down in terms of percentages (20% mothers and children, 40% elderly, etc.)?
1c. Do you feel like your facility is easy for your patients to access in terms of travel costs and time?
1d. What services does your facility provide? (If not mentioned, probe about HIV care, counseling, etc.)

**FACILITATOR → *See Grid 1: HIV Clinical Environment***

**2.** We understand that your facilities offer many services, but today we want to focus on your HIV care services. Thinking about the HIV health care services at your facility, which of these characteristics do they have? Please list by number.

**3.** Are there any characteristics of your facility's HIV services that we have left off, either positive or negative?

**4.** If you had to pick the *three* most important elements of an HIV clinical setting, which of these would you include? Why?

**5.** Please list the three most important elements of an HIV clinical setting for men who have sex with men (by number).

**6.** If you had to rate your comfort level administering HIV prevention services to people who visit your facility on a scale from 1 to 5 (5 being most comfortable), how would you rate yourself?
6a. Please explain why you've given yourself that rating.
6b. Would you change any aspects of your previous or ongoing training to enhance your comfort level administering HIV prevention services?
6c. How well resourced is your facility in terms of providing services for MSM? (Do you have MSM-specific protocols in place?)

**7.** Do you feel you've been trained on working with men who have sex with men in HIV clinical settings? How well?
7a. On average, how many MSM do you see in a typical month?
7b. Is there training or a set of skills that you feel like you're missing when it comes to providing services for that population?

**8.** If you had to rate your comfort level administering HIV prevention services to men who have sex with men on a scale from 1 to 5 (5 being most comfortable), how would you rate yourself?

8a. Please explain why you’ve given yourself that rating.
8b. Have you ever asked a patient seeking HIV testing or care about MSM-specific sexual risk behavior?
8c. What is it about working with MSM that makes you feel most or least comfortable?
8d. When making referrals for medical and mental health follow-up services, how well do your MSM clients comply when compared to other high-risk groups (in other words, is it easy or difficult to get MSM to come back for follow-up care)?

**FACILITATOR → *Refer to Grid 2: HIV Prevention Services. Briefly give definition of each service.***

9**.** Which of these services or strategies have you ever recommended or provided to MSM attending your facility at least once in your lifetime? Please indicate the number.
9a. Which of these services have you recommended or provided to MSM in the past six months?
9b. If we could create the perfect package of HIV prevention services for local MSM, which three of these would you put together for your patients? Why?
9c. Is there anything about your community that makes it difficult for you to administer these services to MSM?
9d. Is there anything about your culture or religion that makes it difficult for you to administer these services to MSM?

We thank you for joining us for this important conversation today. Before we depart, is there anything else that you would like to add or ask us as we begin preparing to design this HIV prevention package?

---

## Focus Group Discussion Visual Aids

**Grid 1: HIV Clinical Environment**

| | |
|---|---|
| 1. 1. Confidentiality of Visit | 2. 2. Friendly Staff |
| 3. 3. Short Wait Time | 4. 4. Same doctor at each visit |
| 5. 5. Clean environment | 6. 6. LGBT Sensitisation Training |
| 7. 7. One-Stop Shop | 8. 8. MSM-Specific Testing Space |
| 9. 9. General Testing Space | 10. 10. MSM-Specific Questions from Provider |

**Grid 2: HIV Prevention Services**

| | | |
|---|---|---|
| **1.** Condoms | **2.** VCT | **3.** PrEP/PEP |
| **4.** Home testing | **5.** HIV Education | **6.** Couples VCT |

| | | |
|---|---|---|
| **7.** SMS Health Advice | **8.** Referral Services | **9.** Lubricant |
