# Supplementary figures and images for: Sibanye Methods for Prevention Packages Program Project Protocol: Pilot Study of HIV Prevention Interventions for Men Who Have Sex With Men in South Africa
Source: JMIR Res Protoc. 2014 Oct 16;3(4):e55. doi: 10.2196/resprot.3737 (PMC4210958; doi:10.2196/resprot.3737)

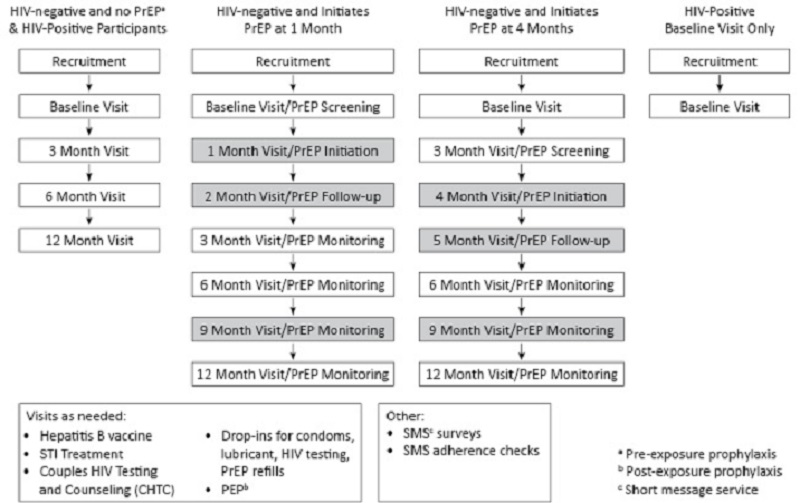

Supplement: Supplementary file 3 [file resprot_v3i4e55_app3.jpg]
