# Supplementary material for: Sibanye Methods for Prevention Packages Program Project Protocol: Pilot Study of HIV Prevention Interventions for Men Who Have Sex With Men in South Africa
Source: JMIR Res Protoc. 2014 Oct 16;3(4):e55. doi: 10.2196/resprot.3737 (PMC4210958; doi:10.2196/resprot.3737)
Supplement: Supplementary file 4 [file resprot_v3i4e55_app4.pdf]

Unique Participant Identifier: CPT_ _ _ /PEL_ _ _ /KMP_ _ _
Date of Interview: Month _____ Date _____

Methods for Prevention Packages Program (MP3)

In-Depth Interview Guide

Welcome Script

Hello, my name is **[insert name]** and I want to thank you for speaking with me today. I am helping to coordinate this study on the health and HIV prevention strategies of men who have sex with men here in **[interview city]**.

What you tell me today will stay between you and me. We will be discussing topics that may be sensitive for you today, but you can trust that the information you tell us will ONLY be used for this research project. There are not right or wrong answers in this conversation. We will simply be discussing your views, opinions and experiences on a range of topics, so please feel comfortable to say what you honestly feel. Our session will last about two hours, and I would like your permission to tape record the session. Please do not be concerned about this: all measures will be taken by the researchers to maintain confidentiality of the interview. During this interview, we will be working on two activities – a timeline of your life and a sexual network map. If at any time during the interview you feel uncomfortable, you can ask for a break, refuse to answer any question, and are always free to leave.

Do you have any questions before we start? Great. Let's begin.

Activity 1: Timeline Activity

Activity Introductory Script:

Our first activity will be to create a timeline of your life, starting from your birth and taking us through to today.

[*Place blank, LAMINATED TIMELINE in front of the participant*]

Here is a blank timeline, with "BIRTH" on one end and "TODAY" on the other. At the beginning of the exercise, I will ask you to put various moments on the timeline in the order they occurred in your life. These will be moments related to your sexual activities and relationships. After you're done, I'll ask you some questions about the timeline you made.

[*Place Worksheet 1: SAMPLE TIMELINE in front of the participant*]

Here's a fictional example of how this activity works. This timeline is about someone's lifetime experience playing football. Let's call this fictional man Sipho.

[*Point to "Watched football with father" Box on Worksheet 1*]

When Sipho was very little, he remembers going with his father to his cousin's house to watch football matches on TV. So he placed a box describing that moment around the time it happened.

[*Point to "Played football with friends" Box on Worksheet 1*]

Around this time, Sipho started playing football with his friends during primary school.

[*Point to "Family bought shin guards and cleats" Box on Worksheet 1*]

Sipho then remembers that when he turned 15 years old, his family bought him a pair of cleats so that he could try out for his school's soccer club, indicated by this box. You can see what Sipho's timeline would look like if he continued describing how football was a part of his life with the moment boxes.

Today, I would like you to build your timeline in a similar way. I am going to give you some boxes that may or may not have happened in your life. For the next 3 to 5 minutes, I would like you to place the moments that happened to you on your timeline in the order and around the time you remember them happening.

[*Give participant laminated Moment Boxes. Give participant 5 minutes to place the boxes on timeline*]

TIMELINE QUESTION GUIDE

I see that you're finished. Now let's talk about the timeline you've created.

[***Throughout the interview, record the participant's responses on your own timeline by moment and age. At the end of each Probe List, ask about Moment Boxes not included on participant's timeline.***]

1. **Sexual Attraction**

So, I noticed you put same-sex attraction/opposite-sex attraction here. How old were you when each sexual attraction moment happened?

Probes:

- How did you react to those feelings?
- Describe how this experience changed the way you viewed yourself.
- Did this change the way other people viewed you, and if so, how?

Sexual Attraction Moment Boxes:

- When you first began to experience same-sex attraction
- When you first began to experience opposite-

2. Sexual Behavior

Next, I'd like us to talk about the sexual experiences you put on your timeline [***Look for Sexual Behavior Moment Boxes on participant's timeline***]. Can you tell me how old you were when each sexual experience happened?

Probes:

- Were these positive or negative experiences? Why?
- What kinds of challenges did this moment cause for you?
- Please describe the way these experiences changed the way you viewed yourself.
- Did this change the way other people viewed you?

Sexual Behavior Moment Boxes:

- When you first had oral sex with a man
- When you first had anal sex with a man
- When you first had oral sex with a woman
- When you first had vaginal sex with a

3. Safer Sex

You say that you began using condoms at this point in time. How old were you when you first learned how to use a condom? How old were you when you actually began using condoms? At what point on your timeline did you begin to use condoms when you had sex with men?

Probes:

- Why did you begin to use condoms?
- Who did you use them with?
- Did you stop using condoms? If so, at what age and why?
- What kinds of challenges did condom use cause for you?
- Please describe the way condom use changed the way you viewed yourself.
- Did your opinion on condom use change the way any partners viewed you?
- Did condom use affect or change your relationship?

Safer Sex Moment Boxes:

- When you first used a condom with a man
- When you first used a condom with a woman
- When you first learned how to properly use a

---

4. Sexual Identity

On your screener form, you told me you call yourself (gay/bisexual/etc.). How old were you when began calling yourself (gay/bisexual/etc.)?

Probes:

Sexual Identity Moment Boxes:

- When you began to identify as heterosexual / straight
- When you began to identify as bisexual
- When you began to identify as homosexual / gay

- Has your sexual identity changed over time, and if so, how?
- What kinds of challenges did this experience cause for you?
- Please describe the way your sexual preferences changed the way you viewed yourself.
- How did this change the way other people viewed you, if at all?

---

5. <u>Closetedness</u>

Many men who have sex with men talk about their experiences "coming out" to friends and family, that is, telling their friends and family about their sexual identity. What prompted you to come out? [***If men excluded one or both of the closetedness Moment Boxes***] I don't see experiences of coming out to your friends/family. What kept you from coming out to your friends/family?

Probes:

- At what age did you first tell your friends about your sexual preference?
- At what age did you first tell your family about your sexual identity, if at all?
- What kinds of challenges did this experience cause for you?
- Please describe the way this experience changed the way you viewed yourself, if at all.
- How did this change the way other people viewed you, if at all?

Closetedness Moment Boxes:

- When you first told your friends about your sexual identity

---

6. <u>Relationships</u>

Tell me about the relationship moments on your timeline. How old were you when these happened?

Probes:

- Did any of these relationships happen at the same time?
- Did you tell your family/friends/colleagues about your boyfriend/partner/husband?
- Please describe the way this experience changed the way you viewed yourself.
- Did this change the way other people viewed you?

Relationship Moment Boxes:

- When you had your first boyfriend
- When you had your first girlfriend
- When you married a woman
- When you married a man

---

7. <u>HIV Testing & Treatment Behavior</u>

Now I would like to move on and talk about moments involving HIV testing and treatment. How old were you when each of these moments happened?

Probes:

- What made you decide to get tested?
- Did you tell your family/friends/colleagues about your HIV status?
- Please describe the way this experience changed the way you viewed yourself.
- Did this change the way other people viewed you?
- Did this change the way other people viewed you?

HIV Moment Boxes:

- When you were first tested for HIV
- When you learned you had HIV
- When you tested negative for HIV
- When began treatment for HIV/AIDS
- [illegible]

---

8. Community Involvement and Everything Else

We're almost finished assembling your timeline. How old were you when each of these remaining moments happened?

Probes:

- What kinds of challenges did these moments cause for you?
- Did this change the way you viewed yourself?
- Did this change the way other people viewed you?

Remaining Moment Boxes:

- When you first joined an LGBT group
- When you interfaced with online LGBT groups

---

9. Conclusion + Resilience

Considering all that we've talked about during this timeline activity, it seems like you've overcome a lot of challenges. How did you make it through? Do you have any advice for other men who may have experienced similar moments?

Would you like to take a break before Activity 2?

Unique Participant Identifier: CPT_ _ _ /PEL_ _ _ /KMP_ _ _

Date of Interview: Month _____ Date _____

Worksheet 1: Sipho's Football Timeline

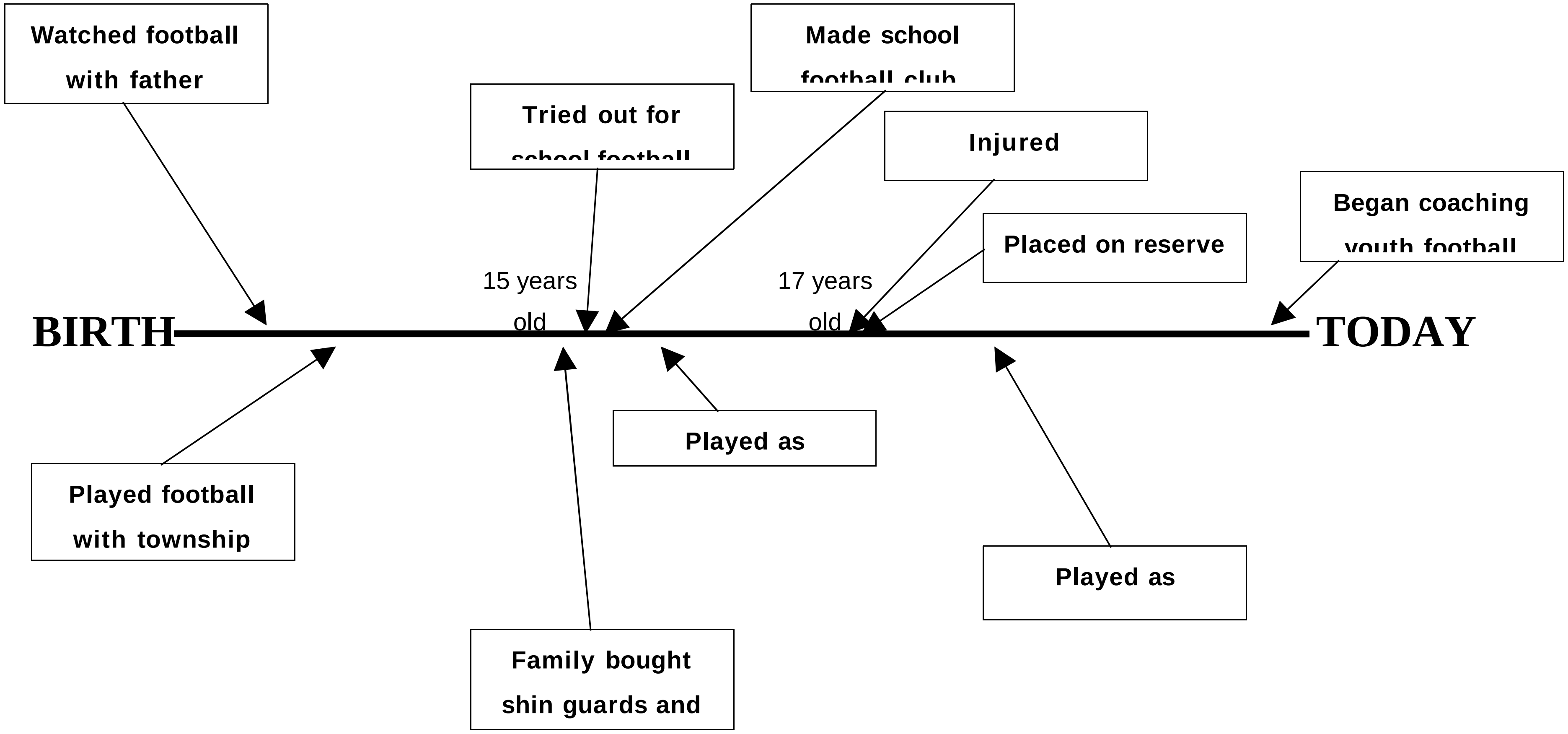

Activity 2: Sexual Network Mapping

Script

Now that I know a little more about your history, I'd like us to move on to our second activity, which will ask you about your recent sexual relationships and the people you feel closest to. If you need it, take a look at this calendar. [Circle Today's Date and the date 6 months prior to it] This is the time period we will be referencing today.

Sexual Network Mapping Question Guide

[Refer to Worksheet 2]

1. In our last activity, we talked about the moment you first joined a group of LGBT people. What general community spaces have you visited often or relied on for services, resources and socializing? These places may include social websites, formal organizations, bars, clubs, informal groups of friends, the gym and other spaces where you spend a lot of free time. [***Complete Community box in Worksheet 2 based on responses***]

2. Please name the first names or nicknames of the people you have felt the closest to in the past 6 months [***Write those names in free space on Worksheet 2***]

3. What categories do each of those people fall in? Are they friends or family? [***Write the names of friends and family members in the appropriate boxes on Worksheet 2***].

4. Which of these people know each other? Do you see these people at any of the community spaces you mentioned previously? [***Draw lines connecting those who know each other and the community spaces they are associated with on Worksheet 2***]

5. How many sex partners have you had in the past 6 months? Were any of the friends you mentioned sex partners in that time period? [***Write the names of sex partners in the appropriate box on Worksheet 2]***

6. Let's discuss the three people closest to you who you have not had sex with in the past 6 months [***Complete Worksheet 3 question-by-question for each of the three closest people the participant wants to discuss. Probe with "why" and "how" after specific questions***].

7. Now let's discuss three of the most important people who have you had sex with in the past 6 months [***Complete Worksheet 4 question-by-question for each of the three sex partners the participant wants to discuss. Probe with "why" and "how" after specific questions***].

That concludes our interview. Thank you so much for your time and honesty.

Do you have any questions before we depart?

Unique Participant Identifier: CPT_ _ _ /PEL_ _ _ /KMP_ _ _
Date of Interview: Month _____ Date _____

Worksheet 2

Instructions: ***Ask the participant the following questions***

1. What community spaces have you visited often and/or relied on for services, resources, and socializing (prompts provided in the top box)? *Write his answers on the lines here →*
2. Please name the first names or nicknames of the people you have felt closest to in the past 6 months. *Write those names in your personal notes.*
3. What categories do those people fall in (friends, family, etc.)?
4. Who knows who? *Draw lines between those who know each other, and the community spaces associated with those mentioned.*
5. How many sex partners have you had in the past 6 months? Were any of the people you mentioned sex partners in that time period?
6. Let's discuss the 3 people closest to you who you have not had sex with in the past 6 months.

Social Network Map

Community
(Websites, Organizations, Bars, Clubs, Informal Groups, Gym, etc.)

YOU

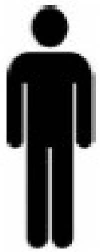

Friends

Family

Sex Partners (*in past 6 months*)

How many?____

How many?____

Unique Participant Identifier: CPT_ _ _ /PEL_ _ _ /KMP_ _ _
Date of Interview: Month _____ Date _____

Worksheet 3: Closest Network Questionnaire

[illegible]

	(Circle one)	(Circle one)	(Circle one)
**Question**	**Partner 1**	**Partner 2**	**Partner 3**
**Nickname**			
**Age**			
**Race/Ethnicity**	White Black Colored Other	White Black Colored Other	White Black Colored Other
**What's this person's sexual identity?**	Straight Gay Bisexual Unsure<br>Out-Yes Out-No	Straight Gay Bisexual Unsure<br>Out-Yes Out-No	Straight Gay Bisexual Unsure<br>Out-Yes Out-No
**Does this person know that you're MSM?**<br>*Why or why not?*	Yes No<br>Reason:	Yes No<br>Reason:	Yes No<br>Reason:
**Does this person know your HIV-status?**	Yes No	Yes No	Yes No
**Is this person in a relationship?**<br>*What type of relationship?*	Yes No<br>Married Dating One Dating Mult. Other	Yes No<br>Married Dating One Dating Mult. Other	Yes No<br>Married Dating One Dating Mult. Other
**How long have you known this person?**	______ Days _______ Weeks ______ Months<br>______ Years	______ Days _______ Weeks ______ Months<br>______ Years	______ Days _______ Weeks ______ Months<br>______ Years
**Has this person ever been to prison?**			
**Have you EVER had sex with this person**			

(excluding the last 6 months)?			

Unique Participant Identifier: CPT_ _ _ /PEL_ _ _ /KMP_ _ _
Date of Interview: Month _____ Date _____

Worksheet 4: Sexual Network Questionnaire

(Only individuals who the participant has had sex with in the past 6 months should be described here. Circle

(Circle one) (Circle one) (Circle one)

Question	Partner 1	Partner 2	Partner 3
**Nickname**			
**Age**			
**Race/Ethnicity**	White Black Colored Other	White Black Colored Other	White Black Colored Other
**What title do you have for this sex partner?**<br>*Does this title mean something to you?*			
**What's this person's sexual identity?**<br>• *If gay, is he/she out?*	Straight Gay Bisexual Unsure<br>Out-Yes Out-No	Straight Gay Bisexual Unsure<br>Out-Yes Out-No	Straight Gay Bisexual Unsure<br>Out-Yes Out-No
**Is this person in a relationship?**<br>• *If yes, what type?*	Yes No<br>Married Dating One Dating Mult. Other	Yes No<br>Married Dating One Dating Mult. Other	Yes No<br>Married Dating One Dating Mult. Other
**How long have you known this person?**	______ Days _______ Weeks ______ Months<br>______ Years	______ Days _______ Weeks ______ Months<br>______ Years	______ Days _______ Weeks ______ Months<br>______ Years
**Do other people know about your sexual relationship?**	Yes No<br>Who?	Yes No<br>Who?	Yes No<br>Who?
**When was the last time you had sex with this person?**<br>• *In that week, how many times did you have oral, vaginal, or anal sex?*<br>• *How often in past month/6 months?*	______________<br>**Week–** Oral Acts: Vaginal Acts: Anal Acts:<br>**Month–** Oral Acts: Vaginal Acts: Anal Acts:<br>**6 Mnts–** Oral Acts: Vaginal Acts: Anal Acts:	______________<br>**Week–** Oral Acts: Vaginal Acts: Anal Acts:<br>**Month –** Oral Acts: Vaginal Acts: Anal Acts:<br>**6 Mnts–** Oral Acts: Vaginal Acts: Anal Acts:	______________<br>**Week–** Oral Acts: Vaginal Acts: Anal Acts:<br>**Month–** Oral Acts: Vaginal Acts: Anal Acts:<br>**6 Mnts–** Oral Acts: Vaginal Acts: Anal Acts:
**Are you the top (insertive) or bottom (receptive) with this partner?**<br>• *About how often?*	Top Bottom<br>Rarely Sometimes Most Times Always	Top Bottom<br>Rarely Sometimes Most Times Always	Top Bottom<br>Rarely Sometimes Most Times Always

**Do you use lubricant with this partner?**<br>• *About how often?*	Yes No<br>Rarely Sometimes Most Times Always	Yes No<br>Rarely Sometimes Most Times Always	Yes No<br>Rarely Sometimes Most Times Always
**Do you use condoms with this partner?**<br>• *About how often?*	Yes No<br>Rarely Sometimes Most Times Always	Yes No<br>Rarely Sometimes Most Times Always	Yes No<br>Rarely Sometimes Most Times Always
**Is money, goods, or another item of value exchanged for sex with this partner?**	Yes No	Yes No	Yes No
**Where do you have sex?**			
**Do you know this person's HIV status?**<br>• *If so, what is it?*	Yes No<br>HIV+ HIV-	Yes No<br>HIV+ HIV-	Yes No<br>HIV+ HIV-
**Does this person know your HIV status?**<br>• *Why or why not?*	Yes No	Yes No	Yes No
**Has this person ever been to prison?**	Yes No Unsure	Yes No Unsure	Yes No Unsure
