# Supplementary material for: Sibanye Methods for Prevention Packages Program Project Protocol: Pilot Study of HIV Prevention Interventions for Men Who Have Sex With Men in South Africa
Source: JMIR Res Protoc. 2014 Oct 16;3(4):e55. doi: 10.2196/resprot.3737 (PMC4210958; doi:10.2196/resprot.3737)
Supplement: Supplementary file 5 [file resprot_v3i4e55_app5.pdf]

Outline of panel review:

I. Introduction/Instructions Page
II. Questions (asked for each scale item, and to compare scales within a construct)
III. Anticipated MSM Stigma
    a. Pinel 1999
    b. Liu 2009
IV. Enacted MSM Stigma
    a. Szymanski 2006
    b. Jeffries 2012
    c. Baral (unpublished)
V. Internalized MSM Stigma
    a. Herek 1997
    b. Smolenski 2010
    c. Ross Rosser 1996
VI. Healthcare MSM Stigma
    a. Baral (unpublished)
VII. Anticipated HIV Stigma
    a. Bunn 2007
    b. Bunn 2007
    c. Sayles 2008
VIII. Enacted HIV Stigma
    a. Bunn 2007
    b. Holzemer 2007
IX. Internalized HIV Stigma
    a. Bunn 2007
    b. Visser 2008
X. Healthcare HIV Stigma
    a. Holzemer

I. INTRODUCTION

Thank you for taking the time today to review stigma scales as part of the Expert Review Panel.

The Sibanye Health Project is a pilot study enrolling 200 MSM in Cape Town and Port Elizabeth, South Africa, followed prospectively for 1 year. Men will be offered an HIV prevention package that includes condom and lubricant options, risk-reduction counseling, couples voluntary counseling and testing, and pre-exposure prophylaxis for eligible men.

This preliminary stigma research aims to use qualitative assessment to adapt measures of MSM-stigma and HIV-stigma. Using previously validated scales, we will seek to maximize their appropriateness for the Southern African MSM context by choosing the most optimal scale, and supplementing it with key items from other scales assessing the same domain. For both MSM and HIV-stigmas, we seek to assess three domains: anticipated, experienced and internalized stigmas. We will additionally address healthcare-specific stigma, which is implicitly a subset of experienced stigma.

The stigma research has three parts.

1. Expert review panel of potential scales
2. Focus group discussions and cognitive interviews with MSM in South Africa to identify any missing items
3. Inclusion of finalized scales in surveys, which will be completed prospectively

As part of the expert review panel, we are asking you to review several validated scales. For each item of the scales, you will rate the overall quality and the appropriateness of the item.

When assessing overall quality, please consider both the match of the item to the construct of interest, and item clarity. When assessing appropriateness, consider appropriateness within the South African context and for MSM populations.

We are seeking to measure stigma from the view of the stigmatized population. Therefore, all men in the prospective study will be asked the stigma items related to MSM, but only HIV positive men will be asked the HIV stigma items.

The above instructions are also in the email we sent, so you can reference them at any time while completing the survey. In addition, the "Stigma Papers" PDF includes publications related to each scale.

II. QUESTIONS

A. Questions were asked after presentation of each scale item, for example:

(from Pinel)

Stereotypes about homosexuals have not affected me personally

	**Very poor**	**Poor**	**Average**	**Good**	**Very Good**
Overall quality of the item	( )	( )	( )	( )	( )
Overall fit of item for South African MSM	( )	( )	( )	( )	( )

Do you have any additional comments about this item?
( ) Yes
( ) No

Provide comments here:
____
____
____

B. A comparative question was asked after multiple scales, for example:

(for example) You have now reviewed the following 2 anticipated MSM stigma scales. Please rank them, with 1 being the best scale to use for South African MSM in this study.
________Pinel 1999
________Liu 2009

Provide comments here:
____
____
____

III. ANTICIPATED MSM STIGMA

SCALE 1. Pinel 1999[1]

Scale Instructions: How strongly do you agree with each questionnaire item?
Scale Response Options: 0 (strongly disagree) to 6 (strongly agree), with a midpoint of 3 (neither agree nor disagree)

1. Stereotypes about homosexuals have not affected me personally. (R)
2. I never worry that my behaviors will be viewed as stereotypical of homosexuals. (R)
3. When interacting with heterosexuals who know of my sexual preference, I feel like they interpret all my behaviors in terms of the fact that I am a homosexual.
4. Most heterosexuals do not judge homosexuals on the basis of their sexual preference. (R)
5. My being homosexual does not influence how homosexuals act with me. (R)
6. I almost never think about the fact that I am homosexual when I interact with heterosexuals. (R)
7. My being homosexual does not influence how people act with me. (R)
8. Most heterosexuals have a lot more homophobic thoughts than they actually express.
9. I often think that heterosexuals are unfairly accused of being homophobic. (R)
10. Most heterosexuals have a problem viewing homosexuals as equals.

SCALE 2. Liu 2009[2]

Scale Instructions: How strongly do you agree with each questionnaire item?
Scale Response Options: Four response options include the following: 1=strongly disagree, 2=disagree, 3=agree, 4=strongly agree

1. Many people unwillingly accept (gay or HIV-positive) individuals
2. (Gay or HIV-positive) individuals would lose their heterosexual partners when these partners knew they were (gay or HIV-positive)
3. Many employers would look down upon a (gay or HIV-positive)man, regardless of his qualifications for the job
4. Many people would treat a (gay or HIV-positive)individual differently than they would treat others
5. Many people have negative attitudes toward people who are (gay or HIV-positive)
6. Many people do not see (gay or HIV-positive)individuals as normal men
7. (Gay or HIV-positive) individuals are not welcome in public gatherings, for example, party, night club or meeting
8. Many families would be disappointed to have a (gay or HIV-positive) son
9. Many people think that gay individuals have HIV and will die of AIDS
10. Many people believe that (gay or HIV-positive) individuals are promiscuous

IV. ENACTED MSM STIGMA

SCALE 1: Szymanski 2006[3]

Scale Instructions: Please think carefully about your life as you answer the questions below. Read each question and then circle the number that best describes events in the past year
Scale Response Options:
Choose 1—If the event has NEVER happened to you;
Choose 2—If the event happened ONCE IN A WHILE (less than 10% of the time);
Choose 3—If the event happened SOMETIMES (10–25% of the time);
Choose 4—If the event happened A LOT (26–49% of the time);

Choose 5—If the event happened MOST OF THE TIME (50–70% of the time);
Choose 6—If the event happened ALMOST ALL OF THE TIME (more than 70% of the time)

Factor 1 Harassment and rejection
12 How many times have you been rejected by friends because you are gay?
14 How many times have you been verbally insulted because you are gay?
10 How many times have you been made fun of, picked on, pushed, shoved, hit, or threatened with harm because you are gay?
13 How many times have you heard anti-gay remarks from family members?
11 How many times have you been rejected by family members because you are gay?
9 How many times have you been called a anti-gay name like homo, fag, or other names?
8 How many times have you been treated unfairly by your family because you are gay?

Factor 2 Workplace and school discrimination
2 How many times have you been treated unfairly by your employer, boss, or supervisors because you are gay?
7 How many times were you denied a raise, a promotion, tenure, a good assignment, a job, or other such thing at work that you deserved because you are gay?
1 How many times have you been treated unfairly by teachers or professors because you are gay?
3 How may times have you been treated unfairly by your co-workers, fellow students, or colleagues because you are gay?

Factor 3 Other discrimination
4 How many times have you been treated unfairly by people in service jobs (by store clerks, waiters, bartenders, waitresses, bank tellers, mechanics, and others) because you are gay?
5 How many times have you been treated unfairly by strangers because you are gay?
6 How many times have you been treated unfairly by people in helping jobs (by doctors, nurses, psychiatrists, caseworkers, dentists, school counselors, therapists, pediatricians, school principals, gynecologists, and others) because you are gay?

SCALE 2. Jeffries 2012[4]

Scale Instructions: Indicate the number of times you experienced the event in the past 12 months. Scale Response Options: Never, Once, 2-3 times, 4 or more times

1. Hit or beaten up because people thought you were homosexual or not manly enough
2. Treated rudely or unfairly because people thought you were homosexual or not manly enough
3. Made fun of or called names because people thought you were homosexual or not manly enough
4. Had to act more manly than usual in order to be accepted
5. Felt uncomfortable in a crowd of heterosexual blacks in your city because people thought you were homosexual or not manly enough

SCALE 3. Baral (validation to be published)

Scale Instructions: In your lifetime (the past 12 months), have any of the following things happened <u>because someone you knew, or assumed, you were attracted to men?</u>
Scale Response Options: Choose from the following options: No, Yes, Does not apply, Don't know

1. I felt excluded from family gatherings.
2. I felt that family members made discriminatory remarks or gossiped.
3. I felt rejected by friends.
4. I lost employment or was dismissed from a job.
5. I was denied educational opportunities, like access to school.

V. INTERNALIZED MSM STIGMA

SCALE 1. Herek 1997[5]

Scale Instructions: Please answer the following questions with how often you feel each applies to you
Scale Response Options: Disagree strongly, Disagree, Agree, Agree strongly

1. I often feel it best to avoid personal or social involvement with other gay/bisexual men.
2. I have tried to stop being attracted to men in general.
3. If someone offered me the chance to be completely heterosexual, I would accept the chance.
4. I wish I weren't gay/bisexual.
5. I feel alienated from myself because of being gay/bisexual.
6. I wish that I could develop more erotic feelings about women.
7. I feel that being gay/bisexual is a personal shortcoming for me.
8. I would like to get professional help in order to change my sexual orientation fromgay/bisexual to straight.
9. I have tried to become more sexually attracted to women

SCALE 2: Smolenski 2010[6], adapted from Ross and Rosser 1996[7]

Scale Instructions: Please answer the following questions with how often you feel each applies to you
Scale Response Options: A 7-point Likert scale ranging from 1 (strongly disagree) to 7 (strongly agree)

Personal Comfort With a Gay Identity

1. Even if I could change my sexual orientation, I wouldn't.
2. I feel comfortable being a homosexual man.
3. Homosexuality is as natural as heterosexuality.

Social Comfort With Gay Men

1. I feel comfortable in gay bars.
2. Social situations with gay men make me feel uncomfortable (R).

Public Identification as Gay

1. I feel comfortable discussing homosexuality in a public situation.
2. I feel comfortable being seen in public with an obviously gay person.

SCALE 3: Ross and Rosser 1996[7]

Scale Instructions: Please answer the following questions with how often you feel each applies to you
Scale Response Options: A 7-point Likert scale ranging from 1 (strongly disagree) to 7 (strongly agree)

Factor 1: Public Identification as Gay

1. I am not worried about anyone finding out that I am gay.
2. I feel comfortable discussing homosexuality in a public setting.
3. Even if I could change my homosexuality, I wouldn't.
4. It is important to me to control who knows about my homosexuality.
5. I feel comfortable about being homosexual.
6. I feel comfortable about being seen in public with an obviously gay man.
7. I would prefer to be more heterosexual.
8. I don't like thinking about my homosexuality.
9. Obviously effeminate homosexual men make me feel uncomfortable.
10. It would not be easier in life to be heterosexual.

Factor 2: Perception of Stigma Associated with Being Gay

1. I worry about becoming old and gay.
2. I worry about becoming unattractive.
3. Society still punishes people for being gay.
4. Most people have negative reactions to homosexuality.
5. Discrimination against gay people is still common.
6. Most people don't discriminate against homosexuals.

Factor 3: Social Comfort with Gay Men

1. I feel comfortable in gay bars.
2. Most of my friends are homosexual.
3. I do not feel confident about making an advance to another man.
4. When I think about other homosexual men, I think of negative situations.
5. Social situations with gay men make me feel uncomfortable.
6. I prefer to have anonymous sexual partners.

Factor 4: Moral and Religious Acceptability of Being Gay

1. Homosexuality is not against the will of God.
2. Homosexuality is morally acceptable.
3. Homosexuality is as natural as heterosexuality.
4. I object if an anti-gay joke is told in my presence.

VI. HEALTHCARE MSM STIGMA

SCALE 1. Baral (validation to be published)

Scale Instructions: In the past 12 months, have any of the following things happened because someone you knew, or assumed, you were attracted to men?
Scale Response Options: Choose from the following options: No, Yes, Does not apply, Don't know

Experience of healthcare stigma

1. In the last 12 months, have any of the following things happened due to your sexual orientation?
2. I felt afraid to go to health care services.
3. I avoided going to health care services.
4. I was denied health care services.
5. I was not treated well when receiving healthcare services.
6. I heard healthcare providers gossiping about me.
7. I was tested for HIV without my permission.

Response options: No, Yes, Does not apply, Don't know

VII. ANTICIPATED HIV STIGMA

SCALE 1. Bunn 2007[8], adapted from Berger 2001[9]

Scale Instructions: To which degree is each question applicable to you?
Scale Response Options: Strongly disagree, Disagree, Agree, Strongly agree

Concern with public attitudes
14. Most people think that a person who is HIV-positive is disgusting.
16. Most HIV-positive people are rejected when others learn that they have HIV/AIDS.
5. Most HIV-positive people lose their jobs when employers learn about it.
10. Most people believe a HIV-positive person is dirty.
20. Most people are uncomfortable around someone who is HIV-positive.
40. Knowing that you are HIV-positive makes others look for flaws in your character.a
9. People who are HIV-positive are treated like outcasts.

SCALE 2. Bunn 2007[8], adapted from Berger 2001[9]

Scale Instructions: To which degree is each question applicable to you?
Scale Response Options: Strongly disagree, Disagree, Agree, Strongly agree

Disclosure concerns
26. I regret having told some people that I am (gay or HIV-positive).
21. I never felt that I have to hide the fact that I am (gay or HIV-positive).b
25. I worry people who know I am (gay or HIV-positive) will tell others.
17. I am very careful who I tell that I am (gay or HIV-positive).
6. I work hard to keep that I am (gay or HIV-positive) a secret.
37. I told people close to me to keep that I am (gay or HIV-positive) a secret.
1. In many areas of my life, no one knows that I am (gay or HIV-positive).
4. Telling someone I am (gay or HIV-positive) is risky.
22. I worry that people may judge me when they learn that I am (gay or HIV-positive).

SCALE 3. Sayles 2008 [10]

Scale Instructions: To which degree is each question applicable to you?
Scale Response Options: None of the time, A little bit of the time, Some of the time, Most of the time, All of the time

Factor 2 = Disclosure concerns

13. I am concerned if I go to the HIV clinic someone I know might see me
14. I am concerned if I have physical changes from the HIV medicines people will know I have HIV
15. I am concerned if I go to an AIDS organization someone I know might see me
16. I am concerned people will find out I have HIV by looking at my medical paperwork
17. I am concerned that if I am sick people I know will find out about my HIV

VIII. ENACTED HIV STIGMA

SCALE 1. Bunn 2007[8], adapted from Berger 2001[9]

Scale Instructions: To which degree is each question applicable to you?
Scale Response Options: Strongly disagree, Disagree, Agree, Strongly agree

Enacted stigma

36. I have lost friends by telling them that I am HIV-positive.
24. I am hurt by how people reacted to learning I am HIV-positive.
28. People avoid touching me if they know I am HIV-positive.
35. I have stopped socializing with some people due to their reactions to me being HIV-positive.
29. People I care about stopped calling me after learning that I am HIV-positive.
39. People seem afraid of me because I am HIV-positive.
33. People have physically backed away from me because I am HIV-positive.
18. Some people who know that I am HIV-positive have grown more distant.
38. People who know that I am HIV-positive ignore my good points.
32. People don't want me around their children once they know that I am HIV-positive.

SCALE 2. Holzemer (2007)[11]

Scale Instructions: In the past three months, how often did the following events happen because of your HIV status?
Scale Response Options: Never, Once or twice, Several times, Most of the time

Verbal abuse

Someone scolded me.
Someone insulted me.
I was blamed for my HIV status.
I was told that I have no future.
was told that God is punishing me.
I was called bad names.
Someone mocked me when I passed by.
People sang offensive songs when I passed by.

Negative self-perception

I felt completely worthless.
I felt ashamed of having this disease.
I felt that I am no longer a person.
I felt that I brought a lot of trouble to my family.
I felt that I did not deserve to live.

Healthcare Neglect

I was discharged from the hospital while still needing care.
I was shuttled around instead of being helped by a nurse.
In the hospital or clinic, my pain was ignored.
I was refused treatment because I was told I was going to die anyway.
At the hospital, I was left in a soiled bed.
I was denied healthcare.
At the hospital/clinic, I was made to wait until last.

Social Isolation

People cut down visiting me.
People ended their relationships with me.
A friend would not chat with me.
Someone stopped being my friend.
People avoided me.

Fear of contagion

I was told to use my own eating utensils.
I was made to drink last from the cup.
I stopped eating with other people.
I was asked to leave because I was coughing.
I was made to eat alone.
I was asked not to touch someone's child.

Workplace Stigma

Someone tried to get me fired from my job.
My employer denied me opportunities.

IX. INTERNALIZED HIV STIGMA

SCALE 1. Bunn 2007[8], adapted from Berger 2001[9]

Scale Instructions: To which degree is each question applicable to you?
Scale Response Options: Strongly disagree, Disagree, Agree, Strongly agree

Negative self-image

15. Being HIV-positive makes me feel that I'm a bad person.
7. I feel I'm not as good as others because I am HIV-positive.
12. Being HIV-positive makes me feel unclean.
23. Being HIV-positive is disgusting to me.
3. People's attitudes about people who are HIV-positive make me feel worse about myself.
2. I feel guilty because I am HIV-positive.
8. I never feel ashamed of being HIV-positive.b

SCALE 2. Visser 2008[12]

Scale Instructions: To which degree is each question applicable to you?
Scale Response Options: Strongly agree, Agree, Disagree, Strongly disagree

Internalized stigma of PLWHA

1. Getting HIV is a punishment for bad ehavior
2. If I was in public or private transport and someone knew I had HIV they would not sit next to me
3. I think my getting HIV was just a matter of bad luck

4. I think less of myself because I have HIV
5. My neighbours would not like me living next door if they knew I had HIV
6. I would understand if people rejected my friendship because I am HIV+
7. I feel it is completely safe for me to handle other people's children (reverse)
8. I have a lot to teach people about life through having HIV (reverse)
9. Because of my HIV people would not date me
10. People are right to be afraid of me because I have HIV
11. I feel that it is my fault that I got HIV
12. Although I have HIV I am a person who deserves as much respect as anyone else
13. Most employers would not employ me because I am HIV+
14. If I drank from a tap and people knew I had HIV they would not drink from the same tap
15. I must have done something wrong to deserve getting HIV
16. I feel ashamed that I have HIV
17. When people know I have HIV I feel uncomfortable around them

X. HEALTHCARE HIV STIGMA

SCALE 1. Holzemer 2007[11]

Scale Instructions: In the past three months, how often did the following events happen because of your HIV status?
Scale Response Options: Never, Once or twice, Several times, Most of the time

1. I was discharged from the hospital while still needing care.
2. I was shuttled around instead of being helped by a nurse.
3. In the hospital or clinic, my pain was ignored.
4. I was refused treatment because I was told I was going to die anyway.
5. At the hospital, I was left in a soiled bed.
6. I was denied healthcare.
7.

1. Pinel EC. Stigma consciousness: the psychological legacy of social stereotypes. *Journal of personality and social psychology.* Jan 1999;76(1):114-128.

2. Liu H, Feng T, Rhodes AG, Liu H. Assessment of the Chinese version of HIV and homosexuality related stigma scales. *Sex Transm Infect.* Feb 2009;85(1):65-69.

3. Szymanski D. Does Internalized Heterosexism Moderate the Link Between Heterosexist Events and Lesbians' Psychological Distress? *Sex Roles.* 2006/02/01 2006;54(3-4):227-234.

4. Jeffries WLt, Marks G, Lauby J, Murrill CS, Millett GA. Homophobia is Associated with Sexual Behavior that Increases Risk of Acquiring and Transmitting HIV Infection Among Black Men Who Have Sex with Men. *AIDS Behav.* May 9 2012.

5. Herek GM, Cogan JC, Gillis JR, Glunt EK. Correlates of Internalized Homophobia in a Community Sample of Lesbians and Gay Men. *Journal of the Gay and Lesbian Medical Association.* 1997;2:17-25.

6. Smolenski DJ, Diamond PM, Ross MW, Rosser BR. Revision, criterion validity, and multigroup assessment of the reactions to homosexuality scale. *Journal of personality assessment.* Nov 2010;92(6):568-576.

7. Ross MW, Rosser BR. Measurement and correlates of internalized homophobia: a factor analytic study. *Journal of clinical psychology.* Jan 1996;52(1):15-21.

8. Bunn JY, Solomon SE, Miller C, Forehand R. Measurement of stigma in people with HIV: a reexamination of the HIV Stigma Scale. *AIDS Educ Prev.* Jun 2007;19(3):198-208.

9. Berger BE, Ferrans CE, Lashley FR. Measuring stigma in people with HIV: psychometric assessment of the HIV stigma scale. *Research in nursing & health.* Dec 2001;24(6):518-529.

10. Sayles JN, Hays RD, Sarkisian CA, Mahajan AP, Spritzer KL, Cunningham WE. Development and psychometric assessment of a multidimensional measure of internalized HIV stigma in a sample of HIV-positive adults. *AIDS Behav.* Sep 2008;12(5):748-758.

11. Holzemer WL, Uys LR, Chirwa ML, et al. Validation of the HIV/AIDS Stigma Instrument - PLWA (HASI-P). *AIDS Care.* Sep 2007;19(8):1002-1012.

12. Visser MJ, Kershaw T, Makin JD, Forsyth BW. Development of parallel scales to measure HIV-related stigma. *AIDS Behav.* Sep 2008;12(5):759-771.
