# Supplementary material for: Sibanye Methods for Prevention Packages Program Project Protocol: Pilot Study of HIV Prevention Interventions for Men Who Have Sex With Men in South Africa
Source: JMIR Res Protoc. 2014 Oct 16;3(4):e55. doi: 10.2196/resprot.3737 (PMC4210958; doi:10.2196/resprot.3737)
Supplement: Supplementary file 6 [file resprot_v3i4e55_app6.pdf]

Methods for Prevention Packages Program (MP3):

MSM Cognitive Interview Question Guide: *Stigma*

1. Interviewer name
2. Note taker name
3. Date of cognitive interview (dd/mm/yyyy) / /
4. Time of cognitive interview
5. City of cognitive interview
6 Study ID of participant

Introduction:

Hi, my name is _____________ and I want to thank you for joining us today. You have been invited to participate in a study on the health and HIV prevention strategies of local men who have sex with men here in Port Elizabeth, South Africa. We are conducting this research to understand your views about stigma against men who have sex with men and stigma against people living with HIV.

Today, we are trying to improve a set of survey questions that will be answered by hundreds of men in a future health research survey. Our goal in the discussion with you is to be able to understand where there are problems with each question, and how to address any problems. For this process, we will first ask you the survey question, next you will give your answer, and then we will discuss with you how you arrived at your answer, and how we can improve the question. We will also read some instruction sections to you, and ask you questions like whether the instructions were clear.

I did not design the instructions or the questions, so feel free to be critical in any way. There are no wrong answers. I would like to tape record the whole session. Please do not be concerned about this: all measures will be taken by the researchers to maintain confidentiality of the interview. As we are tape recording the interview, we would like you to not use your last name, or the last names of any other people we discuss, so that we cannot identify you or other people from the recording. Someone else will be in the room with us to take notes so I can focus on our conversation. The discussion will take about one hour.

If at any time during the interview you feel uncomfortable you can ask for a break, refuse to answer any question, and are always free to leave. Do you have any questions before we start? Great. Let's begin.

QUESTION GUIDE:

"Now I am going to ask you some questions about your experience with being a man who has sex with men (if applicable: "and a person living with HIV")

Interviewer instructions – For the questions below, you will let the participant answer questions on the page, marking their answers for five questions at a time (as many as are displayed on the survey page). After their answers are marked, have the participant discuss the questions. Ask one of the two following probes, or use the list below as needed:

1. "Can you tell me in your own words what this question was asking?"
2. "Can you tell me how you decided on this answer."

Other probes the interviewer can use, as appropriate:

"How did this question make you feel?"
"What does the term 'x' mean to you?"
"How sure are you of your answer to this question?"
"Why do you believe this?"
"How did you arrive at that answer?"
"Was that easy or hard to answer?"
"I noticed that you hesitated – can you tell me what you were thinking?"
"How much have you thought about x?"
"How would you change this question to make it clearer?"
"What other words could be used in this question to help you understand it better?"
"What other response options should be provided, so that all possible responses are available?"

READ ALOUD:

There are different ways of referring to guys we know who have sex with men. We want to respect your preferences. For these next questions, how would you like us to refer to you as a man who has sex with other men? Please pick one:

a) Gay

b) Bisexual

c) Man-loving

d) MSM (man who has sex with men)

e) Queer

f) Probe on other possible categories

We would like to ask you some questions about stigma related to being a <a MSM>.

If people are aware or become aware that you are <a MSM>, how likely is it that they will treat you in the following ways in the <u>future</u> because you are <a MSM>?

	Very unlikely	Unlikely	Neither likely nor unlikely	Likely	Very likely
An employer will look down on me	( )	( )	( )	( )	( )
Family members will have negative attitudes towards me	( )	( )	( )	( )	( )
Friends will avoid me	( )	( )	( )	( )	( )
Family members will not invite me to	( )	( )	( )	( )	( )

social gatherings					
My community will discriminate against me	( )	( )	( )	( )	( )
People at work will assume I am promiscuous	( )	( )	( )	( )	( )
My community will treat me with less respect	( )	( )	( )	( )	( )
Someone will hit, beat up or sexually assault me	( )	( )	( )	( )	( )
I will be called hurtful words when I go outside my home	( )	( )	( )	( )	( )

In the <u>past</u> 12 months, how often did the following happen to you because you were <a MSM>?

	**Never**	**Once**	**2-3 times**	**4 or more times**	**Does not apply**
Hit or beaten up?	( )	( )	( )	( )	( )
Treated rudely or unfairly?	( )	( )	( )	( )	( )
Made fun of or called names?	( )	( )	( )	( )	( )
(replaced below)	( )	( )	( )	( )	( )
Felt uncomfortable in a crowd of <MSM> in your city?	( )	( )	( )	( )	( )
Lost employment or dismissed from job?	( )	( )	( )	( )	( )
Were rejected by family members?	( )	( )	( )	( )	( )
Excluded from activities traditionally reserved for men, like drinking traditional beer or going to pubs?	( )	( )	( )	( )	( )

In the <u>past</u> 12 months, how often did you do the following due to stigma against you as <a MSM>?

	**Never**	**Rarely**	**Sometimes**	**Often**	**Does not**

					apply
I stayed inside to avoid facing <MSM> stigma	( )	( )	( )	( )	( )
I had relationships with girls to hide that I am <a MSM>	( )	( )	( )	( )	( )
I avoided holding hands or being affectionate with a male partner in public environments	( )	( )	( )	( )	( )
I acted more manly than usual in order to be accepted	( )	( )	( )	( )	( )
I acted differently at work to avoid people understanding my identity	( )	( )	( )	( )	( )
I avoided going out at night, such as going dancing	( )	( )	( )	( )	( )

How do you feel about being <a MSM>? Please rate how much you agree with the following statements.

	Strongly Disagree	Disagree	Neutral	Agree	Strongly Agree
If I could change being <a MSM> to be a man who has sex only with women, I would do it.	( )	( )	( )	( )	( )
If people call me names, I am good at ignoring it	( )	( )	( )	( )	( )
(delete)	( )	( )	( )	( )	( )
I feel ashamed of being <a MSM>	( )	( )	( )	( )	( )
Social involvement with other <MSM> makes me feel	( )	( )	( )	( )	( )

uncomfortable.					
I feel I am not as good as others because I am <a MSM>	( )	( )	( )	( )	( )
I think less of myself when I am in public with a person who is obviously <a MSM>	( )	( )	( )	( )	( )
Being <a MSM> is morally acceptable.	( )	( )	( )	( )	( )
Being <a MSM> is against the will of God.	( )	( )	( )	( )	( )
I perceive myself as physically or emotionally weak because I am <a MSM>	( )	( )	( )	( )	( )

In the past 12 months, how often have the following happened to you because someone knew or assumed you were a man who has sex with men?

	**Never**	**Once**	**2-3 times**	**4 or more times**	**Does not apply**
I felt afraid to go to health care services.	( )	( )	( )	( )	( )
I avoided going to health care services.	( )	( )	( )	( )	( )
I was denied health care services.	( )	( )	( )	( )	( )
I was not treated well when receiving healthcare services.	( )	( )	( )	( )	( )
I felt healthcare providers gossiped about me.	( )	( )	( )	( )	( )

Page entry logic: Shown only for HIV-positive men

HIV Stigma scale for HIV+ participants

We would also like to ask you some questions about stigma related to living with HIV.

If people are aware or become aware that you are HIV-positive, how likely is it that they will treat you in the following ways in the future because you are HIV-positive?

	Very unlikely	Unlikely	Neither likely nor unlikely	Likely	Very likely
(delete item)	( )	( )	( )	( )	( )
People will discriminate against me	( )	( )	( )	( )	( )
People will judge me	( )	( )	( )	( )	( )
People will think I am disgusting	( )	( )	( )	( )	( )
People will reject me	( )	( )	( )	( )	( )
People will be uncomfortable around me	( )	( )	( )	( )	( )
People will look for flaws in my character	( )	( )	( )	( )	( )

Page entry logic: Shown only for HIV-positive men

HIV Stigma scale for HIV+ participants

In the past 12 months, how often did the following happen to you because you are HIV-positive?

	Never	Once	2-3 times	4 or more times	Does not apply
I lost friends by telling them I am HIV-positive.	( )	( )	( )	( )	( )
Family members looked down on me.	( )	( )	( )	( )	( )
People treated me with less respect	( )	( )	( )	( )	( )
People didn't want me around their children once they knew that I am HIV-	( )	( )	( )	( )	( )

positive.					
People cut down visiting me once they knew that I am HIV-positive.	( )	( )	( )	( )	( )

Page entry logic: Shown only for HIV-positive men

HIV Stigma scale for HIV+ participants

Please rate how much you agree with the following statements.

	Strongly Disagree	Disagree	Neutral	Agree	Strongly Agree
Being HIV-positive makes me feel that I'm a bad person.	( )	( )	( )	( )	( )
I feel I'm not as good as others because I am HIV-positive.	( )	( )	( )	( )	( )
Being HIV-positive makes me feel unclean.	( )	( )	( )	( )	( )
I never feel ashamed of being HIV-positive.	( )	( )	( )	( )	( )

Page entry logic: Shown only for HIV-positive men

HIV Stigma scale for HIV+ participants

In the past 12 months, how often have the following happened to you because someone knew or assumed you are HIV-positive?

	Never	Once	2-3 times	4 or more times	Does not apply
I was discharged from the hospital or clinic while still needing care.	( )	( )	( )	( )	( )
I was shuttled around instead of being helped by a nurse.	( )	( )	( )	( )	( )
In the hospital or clinic, my pain was ignored.	( )	( )	( )	( )	( )
I was refused treatment because I was told I was going to die anyway.	( )	( )	( )	( )	( )
At the hospital or clinic, I was left in a soiled bed.	( )	( )	( )	( )	( )
I was denied healthcare.	( )	( )	( )	( )	( )

HIV Stigma Scale for HIV-negative participants

Please rate how much you agree with the following statements.

	Strongly Disagree	Disagree	Neutral	Agree	Strongly Agree
<MSM> with HIV should be treated with respect	( )	( )	( )	( )	( )
I am uncomfortable around <MSM> with HIV	( )	( )	( )	( )	( )
I would not want a person with HIV to be around children in my family.	( )	( )	( )	( )	( )

I visited a friend or family member less once I knew they had HIV.	( )	( )	( )	( )	( )
I treat <MSM> with HIV the same as I treat other <MSM>	( )	( )	( )	( )	( )
