# Supplementary material for: Sibanye Methods for Prevention Packages Program Project Protocol: Pilot Study of HIV Prevention Interventions for Men Who Have Sex With Men in South Africa
Source: JMIR Res Protoc. 2014 Oct 16;3(4):e55. doi: 10.2196/resprot.3737 (PMC4210958; doi:10.2196/resprot.3737)
Supplement: Supplementary file 7 [file resprot_v3i4e55_app7.pdf]

CONDOM SCORECARD

For Staff Use

#: ____________ Initials: ________

Date: ____________________

Use this card to rate how much you like each type of condom. Fill out the card soon after using each condom. Bring this card next time you come to the clinic.

	**1 Condom Use:** (tick **all boxes** that apply)			**2 Rating:** (tick **1 box** per condom)				
	"I wore this condom *before* the study."	"I wore this condom *since I started* the study."	"I gave this condom *to my partner* to wear."					
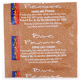 **Impulse** Bare Pleasure	☐	☐	☐	☐	☐	☐	☐	☐
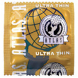 **Atlas** Ultra-Thin	☐	☐	☐	☐	☐	☐	☐	☐
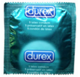 **Durex** Enhanced Pleasure	☐	☐	☐	☐	☐	☐	☐	☐
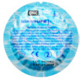 **One** Condom Pleasure Plus	☐	☐	☐	☐	☐	☐	☐	☐
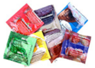 **Trustex** Assorted Flavors	☐	☐	☐	☐	☐	☐	☐	☐
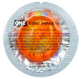 **One** Color Sensations	☐	☐	☐	☐	☐	☐	☐	☐
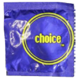 **Choice**	☐	☐	☐	☐	☐	☐	☐	☐
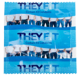 **TheyFit**	☐	☐	☐	☐	☐	☐	☐	☐
