# Supplementary material for: Sibanye Methods for Prevention Packages Program Project Protocol: Pilot Study of HIV Prevention Interventions for Men Who Have Sex With Men in South Africa
Source: JMIR Res Protoc. 2014 Oct 16;3(4):e55. doi: 10.2196/resprot.3737 (PMC4210958; doi:10.2196/resprot.3737)
Supplement: Supplementary file 8 [file resprot_v3i4e55_app8.pdf]

DataFax # 027 Plate # 001 Visit # 000

The Sibanye Health Project

# Screening and Enrollment Form



ⓘ This form is to be completed by Study Staff during the screening and consenting of potential Sibanye study participants. Complete this form for all recruited men who attend a baseline visit.

*Fill in the participant's recruitment ID here:*
**Study ID:** ☐☐ - ☐☐☐☐

*Fill in event ID here:*
**Event ID:** ☐ - ☐☐

**Staff initials:** ☐☐☐ (*F M S*)

ID Verification Code: ☐☐☐☐

**Date completed:** ☐☐ ☐☐ 20☐☐
*day month year*

**Section 1: Dates and Demographics**

1. Date of screening / baseline visit: ☐☐ ☐☐ 20☐☐
*day month year*

2. Participant's date of birth: ☐☐ ☐☐ 19☐☐
*day month year*

*(please tick only one)*
3. Race ☐ Black ☐ Coloured ☐ Indian/Asian ☐ White ☐ Other ______________

4. Has participant previously been enrolled in iPrEx and/or iPrEx OLE? ☐ Yes ☐ No

a) If yes, which were they enrolled in? (*Tick all that apply*) ☐ iPrEx ☐ iPrEx OLE

**Section 2: Criteria**

1. Study eligibility criteria:

| | **Participant Eligible** | |
|---|---|---|
| a. Age at least 18 years old | ☐ Yes | ☐ No |
| b. Currently a resident of study city | ☐ Yes | ☐ No |
| c. Plans to remain in study city for the next year | ☐ Yes | ☐ No |
| d. Able to read and answer survey questions in English, Afrikaans, or Xhosa | ☐ Yes | ☐ No |
| e. Male sex at birth | ☐ Yes | ☐ No |
| f. Had anal sex with a man in the last 12 months | ☐ Yes | ☐ No |
| g. Willing to provide 2 pieces of contact information, including a phone number | ☐ Yes | ☐ No |
| h. Willing to attend study visits at 3, 6, and 12 months | ☐ Yes | ☐ No |
| i. Has a phone | ☐ Yes | ☐ No |

ⓘ *If items a-i under Criteria are marked 'yes', the participant is eligible to be enrolled into the study.*

2. Is the participant eligible to be enrolled into the study?

☐ Yes → *Start consent process & continue to the next page.*

☐ No → *Skip to Section 4 and mark "no" for item a.*

ver: 24/Apr/14

Reviewer's Initials ☐☐☐ (*F M S*) Date sent __/__/__ (*dd mm yy*)

DRAFT

DataFax # 027 Plate # 002 Visit # 000

The Sibanye Health Project

# Screening and Enrollment Form



ⓘ This form is to be completed by study staff during the screening and consenting of potential Sibanye study participants. Complete this form for all recruited men who attend a baseline visit.

*Fill in the participant's recruitment ID here:*
**Study ID:** ☐☐ - ☐☐☐☐

ID Verification Code: ☐☐☐☐

ⓘ Before proceeding, take the participant through the consent form.
If consent is obtained make sure the participant signs and dates both copies of the form. Study staff should sign and date both copies of the consent form as well.
Provide a copy of the consent form to the participant.
Please record the outcome of the consent process in Section 3 below.

**Section 3: Informed Consent**

1. Has written consent been obtained from the participant?

☐ Yes ····➤ a. Date consented: ☐☐ (day) ☐☐ (month) 2 0 ☐☐ (year)

☐ No ····➤ If refused to participate, why? *(tick all that apply)*

| | | |
|---|---|---|
| ☐ Won't be available | ☐ Unwilling to participate in research | ☐ Confidentiality |
| ☐ Doesn't like blood draws | ☐ Too many visits | ☐ Other: ____________________ |
| ☐ Not enough incentive | ☐ No reason given | |

2. Can we contact participant about future research opportunities? ☐ Yes ☐ No

ⓘ *If written consent for Sibanye is not provided, __**the participant cannot be enrolled**__.*
*Mark "No" for item 1 under Section 4 below.*
*If written consent is provided, proceed to* **Contact Information and Preferences Sheet.**
*If* **Contact Information and Preferences Sheet** *completed, mark "Yes" for item 1 under Section 4 below.*
*If* **Contact Information and Preferences Sheet** *not completed, mark "No" for item 1 under Section 4 below.*

DRAFT

**Section 4: Enrollment**

***NOTE:*** *If the participant is eligible, consented and has provided two pieces of contact information, recorded on the* **Contact Information and Preferences Sheet**, *then the participant is successfully enrolled into the study.*

1. Has the participant been enrolled? ☐ Yes ☐ No

Yes ↓ *Fill out study ID and ID verification code on participant ID card, and give to participant. Escort participant to take baseline survey.* ↓

No ↓ *Thank participant for their time and instruct to leave.*

2. Did participant receive ID card? ☐ Yes ☐ No (*Explain why*): ______________

______________________________________

______________________________________

Reviewer's Initials ☐☐☐ (F M S) Date sent ___/___/___ (dd mm yy)

The Sibanye Health Project

# XXXXXX HIV Counseling, Testing & Results Form



ⓘ To be completed by the Counselor. Please complete this form **before** the participant proceeds to the clinical assessment and specimen collection.

Study ID: ☐☐ - ☐☐☐☐

Counselor initials: ☐☐☐ (F M S)

ID Verification Code: ☐☐☐☐

Date completed: ☐☐ (day) ☐☐ (month) 2 0 ☐☐ (year)

**Section 1: Pre-test counseling and HIV testing history**

1. Was pre-test counseling provided? ☐ Yes ☐ No
2. Has the participant ever been tested for HIV? ☐ Yes ☐ No → *skip to question 4*
   - a. Date of last test? ☐☐ (month) ☐☐☐☐ (year)
3. Has participant been previously diagnosed with HIV? ☐ Yes ☐ No → *proceed to question 4*
   - a. Date of 1st HIV diagnosis ☐☐ (month) ☐☐☐☐ (year)
   - b. Is the participant currently on anti-retroviral therapy? ☐ Yes ☐ No
     - 1) When was therapy started? ☐☐ (month) ☐☐☐☐ (year)

ⓘ *If participant is currently on anti-retroviral therapy, please complete the* **XXXXXX ART Self-Report Adherence Form** *and provide Wisepill dispenser.*

4. Has participant ever taken Truvada (PrEP) for the prevention of HIV? ☐ Yes ☐ No → *proceed to question 5*
   - a. Date last on Truvada (PrEP): ☐☐ (month) ☐☐☐☐ (year)
   - b. Currently on Truvada (PrEP): ☐ Yes ☐ No
   - c. Was Truvada (PrEP) prescribed as part of iPrex or iPrex OLE participation? ☐ Yes ☐ No

**During the counseling session, ask participant if he has experienced any social harms as a result of being in the study.**

5. Did participant report experiencing social harms? ☐ Yes ☐ No

ⓘ *If participant reports experiencing social harms, complete the* **Social Harms Reporting Form***.*

Reviewer's Initials ☐☐☐ (F M S) Date sent __/__/__ (dd mm yy)



DRAFT

The Sibanye Health Project

# XXXXXX HIV Counseling, Testing & Results Form



Study ID: ☐☐ - ☐☐☐☐

ID Verification Code: ☐☐☐☐

ⓘ *Administer HIV rapid test at this time. Please do rapid tests based on the local algorithm.*

6. Did the participant mention any of the following risk behaviors during the counseling session? *Tick all that apply.*
   - ☐ a. History of STIs
   - ☐ b. Female sex partners
   - ☐ c. Unprotected intercourse with females
   - ☐ d. Unprotected anal intercourse with males
   - ☐ e. Suspects he might be HIV positive
   - ☐ f. History of injection drug use

7. *Please write any additional notes on information disclosed by the participant during the pre-test counseling session.*

Notes: ______________________________________________
______________________________________________
______________________________________________

**Section 2: HIV Rapid Test Results**

1. What was the result of the HIV rapid test(s)?

**Rapid Test 1:**

a. Test result: *(mark one option)* ☐ Non-reactive ☐ Reactive ☐ Invalid
☐ Not Run (*Explain why*): ______________________________________________
______________________________________________

**Rapid Test 2, if necessary:**

*DTHF always runs 2nd rapid test; HSRC runs if 1st rapid test is invalid or reactive*

b. Retest result: *(mark one option)* ☐ Non-reactive ☐ Reactive ☐ Invalid ☐ Not run

c. Was Rapid Test 2 administered at the same time or after Rapid Test 1?

☐ Rapid Test 2 done at same time ☐ Rapid Test 2 done after ☐ Not run

*DTHF performs ELISA when either rapid test is positive or invalid; HSRC runs ELISA if results of 2 rapid tests are discordant.*

d. Is ELISA test necessary? ☐ Yes ☐ No

2. Based on rapid test results, is participant diagnosed HIV negative, positive, or preliminary positive?

☐ Negative ☐ Positive ☐ Preliminary Positive

Reviewer's Initials ☐☐☐ (F M S) Date sent ___ / ___ / ___ (dd mm yy)



DRAFT

The Sibanye Health Project

# XXXXXX HIV Counseling, Testing & Results Form



Study ID: ☐☐ - ☐☐☐☐

ID Verification Code: ☐☐☐☐

**Section 3: Delivery of HIV Rapid Test Results**

1. Were rapid test results delivered to participant? ☐ Yes ☐ No

*If no, why not?*

☐ Client refused ☐ Client left prior to explanation

☐ Other (please explain) ______________________

*If rapid test results were non-reactive, please complete Sections 5 and 6; if rapid test results were reactive or invalid, please complete Sections 4 and 5.*

**Section 4: Reactive or Invalid Test Result Only**

1. Were details about confirmatory test explained? ☐ Yes ☐ No ☐ Not Applicable → *skip to Section 5*

*If no, why not?*

☐ Client refused ☐ Client left prior to explanation

☐ Other (please explain) ______________________

2. Were instructions provided as to when and how client will receive confirmatory test results? ☐ Yes ☐ No

*If no, why not?*

☐ Client refused ☐ Client left prior to instruction

☐ Other (please explain) ______________________

**Section 5: Risk Reduction Counseling**

1. Was risk reduction counseling completed? ☐ Yes ☐ No

*If no, why not?*

☐ Client refused ☐ Client left prior to receiving counseling

☐ Other (please explain) ______________________

**Section 6: PrEP Information**

*If participant tests negative, please introduce and describe PrEP to participant. The Provider will assess eligibility.*

1. Was PrEP introduced and described to the participant? ☐ Yes ☐ No

Reviewer's Initials ☐☐☐ (F M S) Date sent ___/___/___ (dd mm yy)



DRAFT

The Sibanye Health Project

# XXXXXX ART Self-Reported Adherence Form



ⓘ This form should be completed by the Counselor for all participants that are HIV-positive and on antiretroviral therapy.

Study ID: ☐☐ - ☐☐☐☐

Staff initials: ☐☐☐ (F M S)

ID Verification Code: ☐☐☐☐

Date completed: ☐☐ (day) ☐☐ (month) 2 0 ☐☐ (year)

1. Ask the participant the following statement and record their response:
   *Considering the last 4 weeks, rate your ability to take all your medications as prescribed.*

   - ☐ Very poor
   - ☐ Poor
   - ☐ Fair
   - ☐ Good
   - ☐ Very good
   - ☐ Excellent

ⓘ *Complete adherence assessment and counseling at this time.*

2. Was adherence counseling completed? ☐ Yes ☐ No

DRAFT

Reviewer's Initials ☐☐☐ (F M S) Date sent __/__/__ (dd mm yy)

The Sibanye Health Project

# Baseline Clinical Assessment Form



This form should be completed by the Provider. Review the **Baseline HIV Counseling, Testing and Results Form** for HIV testing and diagnosis history, and HIV rapid test result.

Study ID: ☐☐ - ☐☐☐☐

Provider initials: ☐☐☐ (F M S)

ID Verification Code: ☐☐☐☐

Date completed: ☐☐ (day) ☐☐ (month) 2 0 ☐☐ (year)

Date of assessment: ☐☐ (day) ☐☐ (month) 2 0 ☐☐ (year)

**Section 1: Medical history**

1. Has the participant ever been diagnosed with a STI?

☐ Yes ☐ No ☐ Doesn't know

a. Please tick all STIs that participant has ever been diagnosed with:

☐ Chlamydia *(please specify site)* 1) ☐ Urethral ☐ Rectal ☐ Doesn't know

☐ Genital ulcers

☐ Gonorrhea *(please specify site)* 1) ☐ Urethral ☐ Rectal ☐ Doesn't know

☐ Genital warts (Human papilloma virus)

☐ Syphilis

☐ Trichomoniasis

☐ Other *(please explain)* ----------------------------------------

b. Additional Notes: ----------------------------------------

----------------------------------------

2. Has the participant ever been diagnosed with diabetes ("sugar")?

☐ Yes ☐ No ☐ Doesn't know / Never been tested

**Section 2: Vital signs**

1. Weight: ☐☐☐.☐ kg

2. Height: ☐☐☐ cm

3. Has a healthcare provider ever told the participant he has high blood pressure?

☐ Yes ☐ No ☐ Doesn't know / Never been tested

4. Blood pressure: ☐☐☐ (systolic) / ☐☐☐ (diastolic) mmHg

Reviewer's Initials ☐☐☐ (F M S) Date sent __/__/__ (dd mm yy)



DRAFT

DataFax # 027 Plate # 012 Visit # 001

The Sibanye Health Project

# Baseline Clinical Assessment Form



Study ID: ☐☐ - ☐☐☐☐

ID Verification Code: ☐☐☐☐

**Section 3: Physical signs suggestive of liver disease**

1. Does the participant display any signs suggestive of liver disease?

☐ Yes ☐ No

↓

a. Please specify which signs. *Tick all that apply*:

☐ Spider telangiectasias
☐ Gynecomastia
☐ Parotid hyperplasia
☐ Palmar erythema
☐ Testicular atrophy
☐ Ascites
☐ Liver tenderness
☐ Liver nodularity
☐ Hepatomegaly
☐ Other, specify ______________________

**Section 4: Self-reported circumcision assessment**

1. Does the participant report being circumcised?

☐ Yes ☐ No ☐ Participant not sure

↓

a. If yes, what type of circumcision was done? ☐ Medical provider ☐ Traditional ☐ Participant not sure

**Section 5: Provider assessment of circumcision**

1. Did you complete circumcision assessment? ☐ Yes ☐ No → If no, why not?

*If Yes, please complete a & b below*

☐ Client refused
☐ Other, specify ______________________

a. Please use the grading system below to describe the participant's circumcision status. Only tick one box.

| Grade | Description |
|---|---|
| 1 ☐ | Foreskin covers one-half of the glans; completely uncircumcised |
| 2 ☐ | Foreskin is past the sulcus, but covers less than one-half of the glans |
| 3 ☐ | Foreskin is not past, but can be extended past the sulcus to cover one-half of the glans |
| 4 ☐ | Foreskin is completely absent; completely circumcised |

b. Based on provider assessment of circumcision, should participant be referred for circumcision?

***If Grades 1-3 recorded for Section 5: Question 2, then tick "yes." Otherwise, tick "no."*** ☐ Yes ☐ No

Reviewer's Initials: ☐☐☐ (F M S) Date sent: __/__/__ (dd mm yy)



DRAFT

The *Sibanye* Health Project

# Baseline Clinical Assessment Form



Study ID: ☐☐ - ☐☐☐☐

ID Verification Code: ☐☐☐☐

DRAFT

**Section 6: Assessment of STI symptoms and Rectal Swab Collection**

1. Assessment of penile skin, scrotum and groin area (including foreskin if present)

☐ Normal → *If normal, go to item 2.*
☐ Abnormal
☐ Not done → Why not? ☐ Client refused ☐ Other, specify ____________

a. Please specify type of finding. *Tick all that apply*:

☐ Vesicles ☐ Genital warts
☐ Urethral discharge ☐ Erythema
☐ Genital ulcers ☐ Pain during sexual intercourse
☐ Testicular pain ☐ Painful/burning sensation during urination
☐ Crusts ☐ Other, specify ____________

2. Assessment of perianal and buttocks area } ⓘ ***Collect rectal swab here***

☐ Normal → *If normal, go to item 3.*
☐ Abnormal
☐ Not done → Why not? ☐ Client refused ☐ Other, specify ____________

a. Please specify type of finding. *Tick all that apply*:

☐ Vesicles ☐ Anal itching
☐ Rectal discharge ☐ Erythema
☐ Ulcers ☐ Anal warts
☐ Anal fissures ☐ Painful bowel movements
☐ Crusts ☐ Other, specify ____________

3. Did you collect rectal swab? ☐ Yes ☐ No → If no, why not?

☐ Client refused
☐ Other, specify ____________

Reviewer's Initials ☐☐☐ (F M S) Date sent __/__/__ (dd mm yy)

The Sibanye Health Project

# Baseline Clinical Assessment Form



Study ID: ☐☐ - ☐☐☐☐

ID Verification Code: ☐☐☐☐

4. Did you make a diagnosis of any of the STIs listed in [part a] below?

☐ Yes ☐ No → *If no, quit form.*

*If Yes, please complete a-c below*

a. Which diagnoses did you make? *Tick all that apply:*

☐ Genital ulcers ☐ Genital warts ☐ Chlamydia
☐ Proctitis ☐ Inguinal bubo syndrome ☐ Gonorrhea
☐ Urethritis ☐ Other, specify ______________________________

b. Was treatment prescribed?

☐ Yes ☐ No

1) Which treatment(s) did you prescribe? *Tick all that apply:*

☐ Azithromycin ☐ Ceftriaxone
☐ Cefixime ☐ Valacyclovir
☐ Doxycycline ☐ Benzathine penicillin
☐ Ciprofloxacin ☐ Erythromycin
☐ Podofilox ☐ Imiquimod
☐ Sinecatechins ☐ Acyclovir
☐ Famciclovir
☐ Other, specify ______________________________

c. Is referral needed?

☐ Yes ☐ No

Reviewer's Initials ☐☐☐ F M S Date sent __/__/__ dd mm yy



DRAFT

The Sibanye Health Project

# XXXXXX PrEP Interest & Risk Criteria Form



Study ID: ☐☐ - ☐☐☐☐

Provider initials: ☐☐☐ (F M S)

ID Verification Code: ☐☐☐☐

Date completed: ☐☐ (day) ☐☐ (month) 2 0 ☐☐ (year)

This form should be completed by the Provider during the clinical assessment for all men with a negative HIV rapid test result. This form will be used to assess PrEP interest and behavioral eligibility.

*Provider should discuss PrEP with the participant and answer any questions he might have at this time.*

**Section 1: PrEP Interest**

1. Is the participant interested in taking PrEP and screening for PrEP at this visit? ☐ Yes ☐ No → *If no, quit form*

**Section 2: PrEP Risk Eligibility Criteria**

1. Which of the following did the participant report? *Tick all that apply.*
   - ☐ a. Has multiple partners
   - ☐ b. Engages in transactional sex, including sex workers
   - ☐ c. Uses or abuses drugs and/or drinks alcohol heavily
   - ☐ d. Had more than 1 episode of a STI in the last year
   - ☐ e. Has a HIV-positive partner, especially if the HIV-positive partner is not on antiretroviral therapy
   - ☐ f. Is in a non-monogamous or open relationship with a HIV-negative partner
   - ☐ g. Is unable or unwilling to achieve consistent use of male condoms

2. Based on the above answers, does this participant meet the behavioral eligibility for PrEP? ☐ Yes ☐ No → *If no, quit form*

**Section 3: Participant Willingness**

1. Is participant willing to adhere to daily oral dosing? ☐ Yes ☐ No → *If no, quit form*

2. Is participant is willing to attend maintenance visits every 3 months? ☐ Yes ☐ No → *If no, quit form*

**Section 4: Medical Eligibility**

1. Is the participant preliminary eligible to initiate PrEP based on their clinical assessment? ☐ Yes ☐ No → *If no, quit form*

2. Participant has no know contraindications to FTC/TDF. ☐ Yes ☐ No → *If no, quit form*

*Note: Participants will not be fully eligible until lab results are received and HIV test at 1 month is negative.*

**Section 5: Final Interest & Behavioral Eligibility**

1. If yes is marked for all questions on this page, the participant is considered interested and behaviorally eligible for PrEP. Is yes marked for all questions on this page? ☐ Yes ☐ No

Reviewer's Initials ☐☐☐ (F M S) Date sent __ / __ / __ (dd mm yy)



DRAFT

DataFax # 027 Plate # 020 Visit # 001

The Sibanye Health Project

# Baseline Specimen Collection Form



ⓘ This form should be completed by the Provider or phlebotomist after the clinical assessment for all men.

Study ID: ☐☐ - ☐☐☐☐

Staff initials: ☐☐☐ (F M S)

ID Verification Code: ☐☐☐☐

Date completed: ☐☐ (day) ☐☐ (month) 2 0 ☐☐ (year)

**Section 1: Blood Collection**

*(All participants must complete a blood draw.)*

1. Has the blood draw been completed?

☐ Yes ☐ No → If no, why not?

☐ Client refused ☐ Venipuncture unsuccessful

☐ Other, specify ------------------------------

*If yes:* ↓

ⓘ If no, skip to page 2. Complete ***Specimen Collection Redraw Form*** at a later visit.

2. For all participants, please collect all of the 4 blood vials listed below.

a. **1 purple top EDTA tube**: DBS *[NICD]* → Collected? ☐ Yes ☐ No

b. **1 clotting yellow top tube with serum separator gel**: Syphilis TPPA* *[NICD]* → Collected? ☐ Yes ☐ No

c. **1 purple top EDTA tube**: Hepatitis B tests *[NHLS]* → Collected? ☐ Yes ☐ No

d. **1 clotting yellow top tube with serum separator gel**: Syphilis RPR & PrEP safeties *[NHLS]* → Collected? ☐ Yes ☐ No

1) Tick all required tests (*see Section 2 of* ***Baseline HIV Counseling, Testing and Results Form***):

☐ Syphilis RPR (all participants)

☐ Creatinine (if rapid test algorithm is negative)

☐ Inorganic phosphate (if rapid test algorithm is negative)

☐ ALT/AST (if rapid test algorithm is negative)

*Collect for all participants; NICD will run TPPA test if NHLS local labs indicate RPR positive

3. If participant had reactive or invalid rapid test(s), even if not confirmed HIV-positive, collect the following 3 additional blood vials (*see Section 2 of* ***Baseline HIV Counseling, Testing and Results Form***):

a. **1 white top plasma preparation tube:** HIV viral load *[NHLS]* → Collected? ☐ Yes ☐ No

b. **1 purple top EDTA tube:** HIV CD4 count *[NHLS]* → Collected? ☐ Yes ☐ No

c. **1 clotting yellow top tube with serum separator gel:** HIV ELISA *[NHLS]* *(if indicated by rapid test algorithm)* → Collected? ☐ Yes ☐ No

Reviewer's Initials ☐☐☐ (F M S) Date sent ___/___/___ (dd mm yy)



DRAFT

DataFax # 027 Plate # 021 Visit # 001

The Sibanye Health Project

# Baseline Specimen Collection Form



Study ID: ☐☐ - ☐☐☐☐

ID Verification Code: ☐☐☐☐

**Section 2: Urine Collection**

*(All participants must provide a urine specimen.)*

1. Was a 20 ml urine specimen collected?

   ☐ Yes ☐ No → If no, why not?

   ☐ Client refused ☐ Unable to provide specimen

   ☐ Other, specify ____________________

   *If yes:*

   ⓘ If no, quit form. Complete ***Specimen Collection Redraw Form*** at a later visit.

2. Please complete 1 aliquot of 2 ml for chlamydia and gonorrhea testing at NICD in the Aptima Urine Collection tube. Was this aliquot completed?

   ☐ Yes ☐ No

3. Drug screening dipstick will be completed by study staff using the remaining urine with the Rapid Response Multi-drug One Step Screening Test Panel. Was drug testing completed?

   ☐ Yes ☐ No

   a. If yes, indicate below all positive results (tick all that apply):

   ☐ Methamphetamines (MAMP) ☐ Dagga/cannabis (THC)

   ☐ Opiates (OPI) ☐ Crack/cocaine (COC)

   ☐ None of the above

   b. Provide lot number of drug testing kit:

   ☐☐☐☐

   c. Provide expiration date of drug testing kit:

   ☐☐ ☐☐ 2 0 ☐☐

   *day* *month* *year*

4. If participant tested HIV-negative, the remaining urine should be sent to NHLS for glycosuria and proteinuria testing. Was participant's urine prepared to be sent to NHLS?

   ☐ Yes ☐ No ☐ Not needed

Reviewer's Initials ☐☐☐ *F M S* Date sent __/__/__ *dd mm yy*

DRAFT

DataFax # 027 Plate # 027 Visit # XXX

The Sibanye Health Project

# X Month STI Medication Dispensing Form



ⓘ This form should be completed each time a participant is prescribed medication for STI treatment. This form should be completed by Study Staff.

Study ID: ☐☐ - ☐☐☐☐

Provider initials: ☐☐☐ (F M S)

ID Verification Code: ☐☐☐☐

Date dispensed: ☐☐ (day) ☐☐ (month) 2 0 ☐☐ (year)

**Section 1: Pill Dispensing**

1. Was medication prescribed to the participant during this visit?

   ☐ Yes ☐ No → *Quit form*

2. Was **benzathine penicillin** dispensed at this visit?

   ☐ Yes ☐ No → *If no, proceed to question 3*

   *If yes:* ↓

| |
|---|
| a. Indication |
| b. Dose/Units |
| c. Comments: |

3. Was **oral doxycycline** dispensed at this visit?

   ☐ Yes ☐ No → *If no, proceed to question 4*

   *If yes:* ↓

| |
|---|
| a. Indication |
| b. Dose/Units |
| c. Comments: |

4. Was **cefiaxime** dispensed at this visit?

   ☐ Yes ☐ No → *If no, proceed to question 5*

   *If yes:* ↓

| |
|---|
| a. Indication |
| b. Dose/Units |
| c. Comments: |

Reviewer's Initials ☐☐☐ (F M S) Date faxed __/__/__ (dd mm yy)



ⓘ *Proceed to the next page of this form.*

DRAFT

The Sibanye Health Project

# X Month STI Medication Dispensing Form



Study ID: ☐☐ - ☐☐☐☐

ID Verification Code: ☐☐☐☐

5. Was **ceftriaxone** dispensed at this visit?

☐ Yes ☐ No → *If no, proceed to question 6*

*If yes:*

| |
|---|
| a. Indication |
| b. Dose/Units |
| c. Comments: |

6. Was **any other medication** dispensed at this visit?

☐ Yes ☐ No → *If no, quit form.*

*If yes:*

List each medication dispensed:

a. Other medication 1: ____________________

| |
|---|
| 1) Indication |
| 2) Dose/Units |
| 3) Comments: |

b. Other medication 2: ____________________

| |
|---|
| 1) Indication |
| 2) Dose/Units |
| 3) Comments: |

c. Other medication 3: ____________________

| |
|---|
| 1) Indication |
| 2) Dose/Units |
| 3) Comments: |

Reviewer's Initials ☐☐☐ (F M S) Date faxed __/__/__ (dd mm yy)

DRAFT

DataFax # 027 Plate # 030 Visit # 001

The Sibanye Health Project

# Baseline Referrals Form



ⓘ To be completed by the Counselor. Complete this form **after** the participant has completed the clinical assessment and specimen collection. Check enrollment counts by HIV status prior to beginning session.

Study ID: ☐☐ - ☐☐☐☐

Counselor initials: ☐☐☐ (F M S)

ID Verification Code: ☐☐☐☐

Date completed: ☐☐ (day) ☐☐ (month) 2 0 ☐☐ (year)

**Section 1: Referrals**

| Does the participant need to be referred for the following? | | If Yes, was referral provided? |
|---|---|---|
| 1. HIV treatment and care *(see page 2, section 2 of Baseline HIV Counseling, Testing & Results Form)* | a. ☐ Yes ☐ No | b. ☐ Yes ☐ No |
| 2. Circumcision *(see page 2, section 5: question 3 of Baseline Clinical Assessment Form)* | a. ☐ Yes ☐ No | b. ☐ Yes ☐ No |
| 3. Sexually transmitted infection (STI) treatment *(see page 4, section 6: question 4c of Baseline Clinical Assessment Form)* | a. ☐ Yes ☐ No | b. ☐ Yes ☐ No |
| 4. Alcohol or drug abuse counseling | a. ☐ Yes ☐ No | b. ☐ Yes ☐ No |
| 5. Domestic or couples violence counseling | a. ☐ Yes ☐ No | b. ☐ Yes ☐ No |
| 6. Mental health counseling | a. ☐ Yes ☐ No | b. ☐ Yes ☐ No |
| 7. Other, describe: ____________________ | a. ☐ Yes ☐ No | b. ☐ Yes ☐ No |

**Section 2: Interventions**

1. Was participant interested in couples voluntary counseling and testing (CVCT)?
   ☐ Yes ☐ No → *If no, proceed to question 9*
   a. If Yes, was a coupon provided for transportation? ☐ Yes ☐ No
2. Was the Condom and Lubricant Package provided to the participant? ☐ Yes ☐ No

**Section 3: Prospectively Followed Participants**

1. Will participant be followed prospectively/attend follow-up visits?
   ☐ Yes ☐ No → *If no, proceed to Section 4*
   a. Was the condom fit tool provided and explained to the participant? ☐ Yes ☐ No
   b. Was the condom score card provided and explained to the participant? ☐ Yes ☐ No
   c. Was referral coupon provided to the participant? ☐ Yes ☐ No

ⓘ If participant is on ART and followed prospectively, explain Wisepill and complete **Wisepill Initiation Worksheet** and **Wisepill Distribution Form** at this time.

1) If yes, fill in coupon ID. *(Last 4 digits should match last 4 digits of participant ID.)*
☐ 2 - ☐☐☐☐

**Section 4: Incentive**

1. Was incentive provided to participant and Incentive Log signed? ☐ Yes ☐ No

Reviewer's Initials ☐☐☐ (F M S) Date sent ___/___/___ (dd mm yy)



DRAFT

The Sibanye Health Project

# Baseline Checkout Form



ⓘ This form is to be completed by Study Staff at the end of the participant's visit.

Study ID: ☐☐ - ☐☐☐☐

Study Staff initials: ☐☐☐ (F M S)

ID Verification Code: ☐☐☐☐

Date completed: ☐☐ (day) ☐☐ (month) 2 0 ☐☐ (year)

| | Yes | No | → If No, provide reason why |
|---|---|---|---|
| 1. Was the participant enrolled (see Section 4: Question 1 on **Screening and Enrollment Form**)? | ☐ | ☐ | |
| 2. Did participant and study staff sign the **Study Consent Form**? | ☐ | ☐ | |
| 3. Was the participant's contact information collected on the **Contact Information and Preferences Sheet?** | ☐ | ☐ | |
| 4. Did the participant complete the behavioral questionnaire? (see **Visit Attendee Log**) | ☐ | ☐ | |
| 5. Was the **Baseline HIV Counseling, Testing & Results Form** completed? | ☐ | ☐ | |
| 6. Was the HIV rapid test administered and results delivered? (see Sections 2 and 3 on **Baseline HIV Counseling, Testing & Results Form**) | ☐ | ☐ | |
| ***Items 7-12 relate to the*** **Baseline Clinical Assessment Form:** | | | |
| 7. Was history of STI diagnosis assessed? (Section 1: Question 1) | ☐ | ☐ | |
| 8. Was history of diabetes diagnosis assessed? (Section 1: Question 2) | ☐ | ☐ | |
| 9. Were physical signs of liver disease assessed? (Section 3: Question 1) | ☐ | ☐ | |
| 10. Was circumcision assessed? (Section 5) | ☐ | ☐ | |
| 11. Were STI symptoms assessed? (Section 6: Questions 1, 2 & 4) | ☐ | ☐ | |
| 12. Was rectal swab collected? (Section 6: Question 3) | ☐ | ☐ | |
| 13. Was PrEP eligibility and interest assessed? (See **Baseline PrEP Interest and Risk Criteria Form**) | ☐ | ☐ | |
| ***Items 14-15 relate to the*** **Baseline Specimen Collection Form:** | | | |
| 14. Were all blood aliquots collected? (Section 1) | ☐ | ☐ | |
| 15. Was urine specimen collected and dipsticks completed? (Section 2) | ☐ | ☐ | |
| 16. If needed, was STI treatment distributed? (see Section 6: Question 4 of the **Baseline Clinical Assessment Form** for prescription information, and Section 1 of the **Baseline STI Medication Dispensing Form**? | ☐ | ☐ | |
| 17. Was referral coupon, condom/lubricant package, condom scorecard, and TheyFit instructions given to participant (see **Baseline Referrals Form)**? | ☐ | ☐ | |
| 18. Was the incentive given to the participant and *Incentive Log* signed? | ☐ | ☐ | |

Reviewer's Initials ☐☐☐ (F M S) Date faxed ___/___/___ (dd mm yy)



DRAFT

The *Sibanye* Health Project

# Baseline Laboratory Results Form



ⓘ This form should be completed by the Study Coordinator after all results have been received from the lab.

Study ID: ☐☐ - ☐☐☐☐

Coordinator initials: ☐☐☐ (F M S)

ID Verification Code: ☐☐☐☐

Date completed: ☐☐ (day) ☐☐ (month) 2 0 ☐☐ (year)

**Section 1: CD4 Count and Viral Load**

*This section will be completed only for participants who tested positive for HIV at their baseline visit.*

1. Were CD4+ cell counts obtained? ☐ Yes ☐ No → a. Why not? ______________________________

   b. Date test conducted: ☐☐ (day) ☐☐ (month) 2 0 ☐☐ (year)

   c. CD4+ cell count: ☐☐☐☐ cells/μL

2. Was viral load obtained? ☐ Yes ☐ No → a. Why not? ______________________________

   b. Date test conducted: ☐☐ (day) ☐☐ (month) 2 0 ☐☐ (year)

   c. Viral load detectable? ☐ Yes ☐ No

   d. ☐☐☐☐☐☐☐ copies/mL

**Section 2: Syphilis Results**

*This section will be completed for all participants.*

1. Syphilis RPR: ☐ Positive ☐ Negative ☐ Indeterminate ☐ Not Run, why not? ______________________________

   *If positive:*

   a. Titer: 1 : ☐☐☐

   b. Is this a NEW syphilis infection? ☐ Yes ☐ No

   *If yes:*

   1) Indicate stage: ☐ Primary ☐ Secondary ☐ Early/Latent ☐ Unknown

   c. If RPR positive, syphilis TPPA should have been run. Indicate results below:

   ☐ Positive ☐ Negative ☐ Indeterminate

   ☐ Not Run, why not? ______________________________

Reviewer's Initials ☐☐☐ (F M S) Date sent ___/___/___ (dd mm yy)



DRAFT

The Sibanye Health Project

# Baseline Laboratory Results Form



Study ID: ☐☐ - ☐☐☐☐

ID Verification Code: ☐☐☐☐

**Section 3: Urine and Rectal STI Results**

*This section will be completed for all participants.*

**Urine Results:**

1. Chlamydia: ☐ Positive ☐ Negative ☐ Indeterminate ☐ Not Run, why not? ____________
2. Gonorrhea: ☐ Positive ☐ Negative ☐ Indeterminate ☐ Not Run, why not? ____________

**Rectal Results:**

3. Chlamydia: ☐ Positive ☐ Negative ☐ Indeterminate ☐ Not Run, why not? ____________
4. Gonorrhea: ☐ Positive ☐ Negative ☐ Indeterminate ☐ Not Run, why not? ____________

**Section 4: Creatinine Results and Clearance Rate Calculation**

1. Mean creatinine level: ☐☐☐ μmol/L

Use the formula below to calculate the creatinine clearance rate and record this rate in a below.

$$\text{Creatinine clearance rate} = \frac{(140 - \underbrace{\square\square}_{\text{Age}} \text{ yrs}) \times (\underbrace{\square\square\square.\square}_{\text{Weight}} \text{ kg})}{0.82 \times (\underbrace{\square\square\square}_{\text{Mean creatinine level}} \text{ μmol/L})}$$

a. Creatinine clearance rate = ☐☐☐ mL/min

2. Is the creatinine clearance rate less than 60 mL/min? ☐ Yes ☐ No

**Section 5: Other PrEP Lab Results**

| | | To Assess PrEP Eligibility: | |
|---|---|---|---|
| 1. Mean AST level: | ☐☐☐ IU/L → | a. Less than 2 x ULN? | ☐ Yes ☐ No |
| 2. Mean ALT level: | ☐☐☐ IU/L → | a. Less than 2 x ULN? | ☐ Yes ☐ No |
| 3. Mean phosphorus level: | ☐☐.☐ mg/dL → | a. Hypophosphatemia? (less than 2.5 mg/dL) | ☐ Yes ☐ No |
| 4. Proteinuria: | ☐ Negative ☐ 1+ ☐ 3+ ☐ Trace ☐ 2+ ☐ 4+ → | a. Less than 2+? | ☐ Yes ☐ No |
| 5. Glycosuria | ☐ Negative ☐ 1+ ☐ 3+ ☐ Trace ☐ 2+ ☐ 4+ → | a. Less than 2+? | ☐ Yes ☐ No |

Reviewer's Initials: ☐☐☐ (F M S) Date sent: __/__/__ (dd mm yy)



DRAFT

The Sibanye Health Project

# Baseline Laboratory Results Form



Study ID: ☐☐ - ☐☐☐☐

ID Verification Code: ☐☐☐☐

**Section 6: Hepatitis B Test Results**

*This section will be completed for all participants.*

1. HBsAg test result: ☐ Negative ☐ Positive ☐ Not run
2. HBsAb test result: ☐ Negative ☐ Positive ☐ Not run
3. HBcAb test result: ☐ Negative ☐ Positive ☐ Not run
4. Anti-HBc IgM result: ☐ Negative ☐ Positive ☐ Not run
5. Interpretation (tick only one):
   - ☐ NOT IMMUNE/SUSCEPTIBLE: If HBsAg negative, HBsAb negative, HBcAb negative
   - ☐ IMMUNE from infection: If HBsAg negative, HBsAb positive, HBcAb positive
   - ☐ IMMUNE from vaccine: If HBsAg negative, HBsAb positive, HBcAb negative
   - ☐ CHRONIC INFECTION: If HBsAg positive, HBsAb negative, HBcAb positive, Anti-HBc IgM negative
   - ☐ ACUTE INFECTION: If HBsAg positive, HBsAb negative, HBcAb positive, Anti-HBc IgM positive
   - ☐ UNCLEAR: If HBsAg negative, HBsAb negative, HBcAb positive

ⓘ If participant is not IMMUNE/SUSCEPTIBLE, they should be offered Hepatitis B vaccination.

**Section 7: Participant Follow-up**

1. Did participant have positive STI results or ELISA confirmatory test that require participant to return to receive results?

   ☐ Yes ☐ No

DRAFT

Reviewer's Initials ☐☐☐ (F M S) Date sent __/__/__ (dd mm yy)

DataFax # 027 Plate # 064 Visit # XX0

The Sibanye Health Project

# X Month Counseling and Linkage to Care Form



ⓘ To be completed by the Counselor for **all participants who tested positive for HIV at a prior study visit**. Please complete this form **before** the participant proceeds to the clinical assessment and specimen collection.

Study ID: ☐☐ - ☐☐☐☐

Counselor initials: ☐☐☐ (F M S)

ID Verification Code: ☐☐☐☐

Date completed: ☐☐ (day) ☐☐ (month) 2 0 ☐☐ (year)

DRAFT

**Section 1: HIV Treatment History**

1. Is participant currently on anti-retroviral therapy? ☐ Yes ☐ No → *proceed to Section 2*

a. When was therapy started? ☐☐ (month) ☐☐☐☐ (year)

ⓘ *If participant is currently on anti-retroviral therapy, please complete the* **X Month ART Self-Report Adherence Form**.

**Section 2: Social Harms Reporting**

*During the counseling session, ask participant if he has experienced any social harms as a result of being in the study.*

1. Did participant report experiencing social harms? ☐ Yes ☐ No

ⓘ *If participant reports experiencing social harms, complete the* **Social Harms Reporting Form**.

**Section 3: Risk Reduction Counseling**

1. Did the participant mention any of the following risk behaviors during the counseling session? *Tick all that apply.*

☐ a. History of STIs

☐ b. Female sex partners

☐ c. Unprotected intercourse with females

☐ d. Unprotected anal intercourse with males

☐ e. Suspects he might be HIV positive

☐ f. History of injection drug use

2. Was risk reduction counseling completed? ☐ Yes ☐ No

*If no, why not?*

☐ Client refused ☐ Client left prior to receiving counseling

☐ Other (please explain)______________________

Reviewer's Initials ☐☐☐ (F M S) Date sent ___/___/___ (dd mm yy)

ver: 24/Apr/14

The Sibanye Health Project

# Wisepill Distribution Form



To be completed by Study Staff when a Wisepill canister is distributed to a participant on PrEP or ARVs. If the canister is replaced, update Section 2 and, if necessary, Section 3 of this form, and resend it to DataFax.

Study ID: ☐☐ - ☐☐☐☐

Staff initials: ☐☐☐ (F M S)

ID Verification Code: ☐☐☐☐

Date completed: ☐☐ (day) ☐☐ (month) 20☐☐ (year)

**Section 1: Wisepill Distribution**

1. Visit Type

☐ Baseline ☐ Month 1 ☐ Month 2 ☐ Month 3 ☐ Month 4 ☐ Month 5 ☐ Month 6

☐ Month 9 ☐ Month 12 ☐ Other (please specify) ____________________

a. If other visit, indicate visit date: ☐☐ (day) ☐☐ (month) 20☐☐ (year)

2. Was Wisepill canister distributed to participant?

☐ Yes ☐ No

a. Record Wisepill canister ID: ☐☐☐ - ☐☐☐☐

**Section 2: First Replacement**

Complete this section if the Wisepill canister is replaced at any point in the study.

1. Indicate why the Wisepill canister was replaced: ____________________

____________________

2. Record new Wisepill canister ID: ☐☐☐ - ☐☐☐☐

**Section 3: Second Replacement**

Complete this section if the Wisepill canister is replaced for a second time at any point in the study.

1. Indicate why the Wisepill canister was replaced: ____________________

____________________

2. Record new Wisepill canister ID: ☐☐☐ - ☐☐☐☐

Reviewer's Initials ☐☐☐ (F M S) Date sent __/__/__ (dd mm yy)



DRAFT

DataFax # 027 Plate # 100 Visit # 002

The Sibanye Health Project

# Baseline PrEP Lab Eligibility Form



ⓘ This form should be completed by study staff after all results have been received from the lab for all participant who test HIV negative at baseline.

Study ID: ☐☐ - ☐☐☐☐

Coordinator initials: ☐☐☐ (F M S)

ID Verification Code: ☐☐☐☐

Date completed: ☐☐ (day) ☐☐ (month) 2 0 ☐☐ (year)

DRAFT

**Section 1: PrEP Lab Eligibility**

**PrEP Eligible**

**From Baseline HIV Counseling, Testing & Results Form:**

1. Is the participant HIV positive? ☐ Yes ☐ No
   *(see section 2 on page 2 of Baseline HIV Counseling, Testing & Results Form)*

**From Baseline Clinical Assessment Form:**

2. Has the participant ever been diagnosed with diabetes? ☐ Yes ☐ No
   *(see section 1: question 2 on page 1 of Baseline Clinical Assessment Form)*
3. Does the participant display any signs suggestive of liver disease? ☐ Yes ☐ No
   *(see section 3: question 1 on page 2 of Baseline Clinical Assessment Form)*

**From Baseline Specimen Collection Form:**

4. Does the participant have a proteinuria result of 2+ or greater? ☐ Yes ☐ No
   *(see section 2: question 5 on page 2 of Baseline Specimen Collection Form for HIV Negatives)*
5. Does the participant have a glycosuria result of 2+ or greater? ☐ Yes ☐ No
   *(see section 2: question 6 on page 2 of Baseline Specimen Collection Form for HIV Negatives)*

**From Baseline Laboratory Results Form:**

6. Does the participant have a creatinine clearance rate of less than 60 mL/min ☐ Yes ☐ No
   *(see section 5 on page 2 of Baseline Laboratory Results Form)*
7. Does participant have acute or chronic Hepatitis B infection? ☐ Yes ☐ No
   *(see section 4: question 3 on page 2 of Baseline Laboratory Results Form)*
8. Is participant's AST level ≥ 2 x ULN? ☐ Yes ☐ No
   *(see section 6: question 1 on page 2 of Baseline Laboratory Results Form)*
9. Is participant's ALT level ≥ 2 x ULN? ☐ Yes ☐ No
   *(see section 6: question 2 on page 2 of Baseline Laboratory Results Form)*

***Eligible***

ⓘ If the response to items 1-9 above is "No" then the participant is lab eligible for PrEP based on their baseline visit. At the 1 month visit, if the participant's rapid test is HIV negative, they are willing to take hepatitis B vaccine (if susceptible) and complete informed consent, participant can start PrEP at the 1 month visit.

10. Is the participant lab eligible to receive PrEP? ☐ Yes ☐ No

Reviewer's Initials ☐☐☐ (F M S) Date faxed __ / __ / __ (dd mm yy)

The Sibanye Health Project

# X Month PrEP Enrollment Form



This form should be completed for all participants with a non-reactive rapid test(s). It should be completed by the Provider after a participant has completed HIV counseling and testing.

Study ID: ☐☐ - ☐☐☐☐

Provider initials: ☐☐☐ (F M S)

ID Verification Code: ☐☐☐☐

Date completed: ☐☐ (day) ☐☐ (month) 2 0 ☐☐ (year)

**Section 1: PrEP Risk Re-screening**

1. Which of the following did the participant report? *Tick all that apply.*
   - ☐ a. Has multiple partners
   - ☐ b. Engages in transactional sex, including sex workers
   - ☐ c. Uses or abuses drugs and/or drinks alcohol heavily
   - ☐ d. Had more than 1 episode of a STI in the last year
   - ☐ e. Has a HIV-positive partner, especially if the HIV-positive partner is not on antiretroviral therapy
   - ☐ f. Is in a non-monogamous concordant relationship with a HIV-negative partner
   - ☐ g. Is unable or unwilling to achieve consistent use of male condoms
2. Based on the above answers, does this participant meet the behavioral eligibility for PrEP? ☐ Yes ☐ No → *Quit form*

**Section 2: Clinical Screening**

1. Was rapid test result(s) at this visit negative? ☐ Yes ☐ No → *Quit form*
2. Participant shows no signs of acute HIV infection. ☐ Yes ☐ No → *Quit form*
3. Was the participant lab eligible for PrEP based on their X Month lab results? ☐ Yes ☐ No → *Quit form*
4. Was participant treated for any STIs diagnosed at their baseline visit? ☐ Yes ☐ No → *Quit form*
5. Is participant susceptible to Hepatitis B infection and initiating vaccine series or previously immune to Hepatitis B from a vaccine or infection? ☐ Yes ☐ No → *Quit form*

***Eligible for PrEP***

6. If yes is marked for question 1 through 5 above, participant is eligible to begin the PrEP informed consent process. Is participant eligible? ☐ Yes ☐ No → *Quit form*

**Continue to the next page**

Reviewer's Initials ☐☐☐ (F M S) Date sent __/__/__ (dd mm yy)



DRAFT

The Sibanye Health Project

# X Month PrEP Enrollment Form



Study ID: ☐☐ - ☐☐☐☐

ID Verification Code: ☐☐☐☐

> ⓘ Before proceeding, take the participant through the PrEP consent form.
> If consent is obtained make sure the participant signs and dates both copies of the form. The Provider should sign and date both copies of the consent form as well.
> Provide a copy of the consent form to the participant.
> Please record the outcome of the consent process in Section 2 below.

**Section 3: Informed Consent and PrEP Enrollment**

1. Has written consent for PrEP been obtained from the participant?

☐ Yes ····➤ a. Date consented: ☐☐ (*day*) ☐☐ (*month*) 2 0 ☐☐ (*year*)

☐ No ····➤ b. If refused to participate, why? *(tick all that apply)*

- ☐ Too many visits
- ☐ Does not want side effects
- ☐ Not enough incentive
- ☐ Does not believe he is at risk
- ☐ No reason given
- ☐ Other: ___________________________

**NOTE:** *If the participant is eligible and consented, then the participant is successfully enrolled in PrEP.*

2. Has the participant been enrolled in PrEP? ☐ Yes ☐ No ⟶ *Quit form*

**Section 4: Adherence Counseling**

1. Was adherence counseling completed? ☐ Yes ☐ No

> ⓘ If participant is enrolled in PrEP, study staff must complete the **X Month PrEP Medication Dispensing Form**.
>
> Staff must also distribute and explain the Wisepill containers, and complete the **Wisepill Distribution Form** and **Wisepill Initiation Worksheet**.

DRAFT



Reviewer's Initials: ☐☐☐ (*F M S*) Date sent: ___ / ___ / ___ (*dd mm yy*)

DataFax # 027 Plate #109 Visit # XX0

The Sibanye Health Project

# X Month PrEP Medication Dispensing Form



ⓘ This form should be completed at the PrEP initiation visit after all eligibility criteria is assessed. It should be completed by Study Staff when FTC/TDF pills are dispensed.

Study ID: ☐☐ - ☐☐☐☐

Provider initials: ☐☐☐ (F M S)

ID Verification Code: ☐☐☐☐

Date dispensed: ☐☐ (day) ☐☐ (month) 2 0 ☐☐ (year)

**Section 1: Verify PrEP Eligibility Criteria**

1. Check Section 3 of the **X Month PrEP Enrollment Form** and verify that the participant is consented and enrolled in PrEP. Please mark here whether the participant is enrolled in PrEP. ☐ Yes ☐ No

ⓘ If yes, the participant can be dispensed pills.

**Section 2: Pill Dispensing**

1. Were bottles dispensed at this visit?

☐ Yes ☐ No → a. Why not? ____________________
____________________

b. How many bottles were dispensed? ☐☐

2. Bottles dispensed at this visit:

| | Kit Number | Bottle Number | # of Tablets Dispensed |
|---|---|---|---|
| a. | 1) ☐☐☐☐ | 2) ☐☐ | 3) ☐☐ |
| b. | 1) ☐☐☐☐ | 2) ☐☐ | 3) ☐☐ |
| c. | 1) ☐☐☐☐ | 2) ☐☐ | 3) ☐☐ |

3. Are there any special circumstances related to this visit? If yes, please describe.

____________________
____________________
____________________

Reviewer's Initials ☐☐☐ (F M S) Date faxed ___/___/___ (dd mm yy)



DRAFT

DataFax # 027 Plate # 112 Visit # XX0

The Sibanye Health Project

# X Month PrEP Return Visit and Adherence Form



ⓘ This form should be completed during each follow-up visit for participants who have initiated PrEP. It should be completed by Study Staff.

Study ID: ☐☐ - ☐☐☐☐

Study Staff initials: ☐☐☐ (F M S)

ID Verification Code: ☐☐☐☐

Date completed: ☐☐ (day) ☐☐ (month) 2 0 ☐☐ (year)

**Section 1: PrEP Pill Bottle Return**

1. Date of last pill dispensation: ☐☐ (day) ☐☐ (month) 2 0 ☐☐ (year)

2. Please fill out the following fields using the bottles and pills returned by the participant:

| | Kit Number* | Bottle Number* | # of Tablets Dispensed* | # of Tablets Returned | # of Tablets Used 3) minus 4) | Check if bottle lost |
|---|---|---|---|---|---|---|
| a. | 1) ☐☐☐☐ | 2) ☐☐ | 3) ☐☐ | 4) ☐☐ | 5) ☐☐ | e. ☐ |
| b. | 1) ☐☐☐☐ | 2) ☐☐ | 3) ☐☐ | 4) ☐☐ | 5) ☐☐ | j. ☐ |
| c. | 1) ☐☐☐☐ | 2) ☐☐ | 3) ☐☐ | 4) ☐☐ | 5) ☐☐ | o. ☐ |

*These fields should be filled out based on prior visit's dispensing form.

**Section 2: Calculate Raw Adherence Score**

*If all bottles dispensed were returned, calculate the raw adherence score as follows using values from question 2. You can also calculate using the spreadsheet "Raw Adherence Score Calculation"*

1. # of Pills Used: a5 ___ + b5 ___ + c5 ___ = _________

2. # of Days Since Last Refill: Today's date _________ - 1. Date of last pill dispensation _________ = _________

Raw Adherence Score = 1: # of Pills Used _________ % 2: # of Days Since Last Refill _________

= 3. ____________________

**Section 3: Self-Reported Adherence**

1. Ask the participant the following statement and record their response:
   *Considering the last 4 weeks, rate your ability to take all your medications as prescribed.*

☐ Very poor ☐ Fair ☐ Very good
☐ Poor ☐ Good ☐ Excellent

**Section 4: Adherence Counseling**

1. Was adherence counseling provided? ☐ Yes ☐ No

Reviewer's Initials ☐☐☐ (F M S) Date faxed __/__/__ (dd mm yy)



DRAFT

The Sibanye Health Project

# PrEP Medication Hold or Discontinuation Form #X



To be used to document temporary clinical holds and clinical permanent discontinuations of PrEP use as instructed by study site staff.

Study ID: ☐☐ - ☐☐☐☐

Staff initials: ☐☐☐ (F M S)

ID Verification Code: ☐☐☐☐

Date completed: ☐☐ ☐☐ 2 0 ☐☐ (day month year)

1. When was the PrEP hold initiated? ☐☐ ☐☐ 2 0 ☐☐ (day month year)

2. Visit when the PrEP hold was initiated: (*Tick only one*)

☐ Month 2 ☐ Month 3 ☐ Month 5 ☐ Month 6

☐ Month 9 ☐ Month 12 ☐ Other (please specify) ________________

a. If other visit, indicate visit date: ☐☐ ☐☐ 2 0 ☐☐ (day month year)

3. Why is PrEP being held? (*Tick all that apply*)

☐ a. Positive HIV test result

☐ b. Adverse event → Complete **Adverse Event Form**

☐ c. Hepatitis B infection

☐ d. Allergic reaction → Complete **Adverse Event Form**

☐ e. Initiated PEP for HIV exposure

☐ f. Missed PrEP visit/PrEP not dispensed

☐ g. Other, please specify: ________________

☐ h. Participant initiated discontinuation

1) Indicate why: *(Tick all that apply)*

☐ a. Too many visits ☐ d. No longer at risk

☐ b. Side effects ☐ e. No reason given

☐ c. Not enough incentive ☐ f. Other: ________________

4. Date of last PrEP use: ☐☐ ☐☐ 2 0 ☐☐ (day month year)

5. Was the participant instructed to resume PrEP use?

☐ Yes → *Record scheduled date of reinitiation* a. ☐☐ ☐☐ 2 0 ☐☐ (day month year)

☐ No, PrEP was permanently discontinued
*i.e. positive HIV test result, serious adverse event*

ver: 27/Jan/14

Reviewer's Initials ☐☐☐ (F M S) Date sent __/__/__ (dd mm yy)

DRAFT

DataFax # 027 Plate # 150 Visit # 46X

The Sibanye Health Project

# CVCT Session Form #X



ⓘ This form should be completed by the Counselor when a participant attends a couples voluntary counseling and testing (CVCT) session with a partner. Complete this form after the session if partner(s) are not aware of the participant's enrollment in the study. If more than one person in the session is a participant in the study, complete a separate CVCT session form for EACH participant.

Study ID: ☐☐ - ☐☐☐☐

Staff initials: ☐☐☐ (F M S)

ID Verification Code: ☐☐☐☐

Date completed: ☐☐ (day) ☐☐ (month) 2 0 ☐☐ (year)

**Section 1: Basic Information & Counseling**

1. Date of CVCT session: ☐☐ (day) ☐☐ (month) 2 0 ☐☐ (year)

2. How many people are receiving CVCT today? ☐ 2 ☐ 3 ☐ 4 ☐ More than 4

3. Is the partner or partners enrolled in Sibanye as well? ☐ Yes ☐ No

   a. If yes, please record the Study IDs of partners enrolled in Sibanye:

   1) Partner 1 ☐☐ - ☐☐☐☐ 2) Partner 2 ☐☐ - ☐☐☐☐

   3) Partner 3 ☐☐ - ☐☐☐☐ 4) Partner 4 ☐☐ - ☐☐☐☐

4. Which of the following elements of CVCT were used in counseling? *Tick all that apply.*

   - ☐ a. Description of rapid test, results, meanings
   - ☐ b. Discussion of HIV discordance
   - ☐ c. Discussion of couple's relationship
   - ☐ d. Discussion of how couple decided to test together, reasons for testing
   - ☐ e. Discussion of couple's HIV risk concerns
   - ☐ f. Discussion of couple's agreement
   - ☐ g. Discussion of communication plan if agreement is broken
   - ☐ h. Creation of couples-based risk reduction plan
   - ☐ i. Discussion of couples-based disclosure plan
   - ☐ j. None of these

DRAFT

Reviewer's Initials ☐☐☐ (F M S) Date sent ___/___/___ (dd mm yy)

The *Sibanye* Health Project

# CVCT Session Form #X



Study ID: ☐☐ - ☐☐☐☐

ID Verification Code: ☐☐☐☐

**Section 2: HIV Rapid Test**

1. Was a HIV rapid test performed on this participant?

☐ Yes ☐ No → a. Why was HIV test not performed?

☐ Participant prevalent positive ☐ Participant refused

☐ Other (please explain) ________________

*Continue to Section 3*

ⓘ *Administer HIV rapid tests at this time. Please do rapid tests based on the local algorithm.*

**Section 3: HIV Results**

1. What was the result of the this participant's rapid HIV test?

☐ Non-reactive ☐ Reactive ☐ Invalid *(Any invalid result should be re-tested)*

a. Re-test result: *(mark one option)* ☐ Non-reactive ☐ Reactive ☐ Invalid

2. Did this participant have a newly diagnosed HIV infection during the session? ☐ Yes ☐ No

3. Were rapid test results delivered to this participant?

☐ Yes ☐ No → *a. If no, why not?*

☐ Client refused ☐ Client left prior to explanation

☐ Other (please explain) ________________

4. Was post-test counseling provided? ☐ Yes ☐ No

5. Did this participant disclose his status to his partner? ☐ Yes ☐ No ☐ Not Applicable

6. Did his partner(s) disclose his status to him? ☐ Yes ☐ No ☐ Not Applicable

**Section 4: Reactive or Invalid Test Result Only**

1. Were details about confirmatory test explained?

☐ Yes ☐ No → *a. If no, why not?*

☐ Client refused ☐ Client left prior to explanation

☐ Other (please explain) ________________

2. Were instructions provided as to when and how client will receive confirmatory test results?

☐ Yes ☐ No → *a. If no, why not?*

☐ Client refused ☐ Client left prior to explanation

☐ Other (please explain) ________________

Reviewer's Initials ☐☐☐ F M S — Date sent __/__/__ dd mm yy



DRAFT

The Sibanye Health Project

# CVCT Session Form #X



Study ID: ☐☐ - ☐☐☐☐

ID Verification Code: ☐☐☐☐

DRAFT

**Section 5: Prevention Plan Discussion**

1. Was a Prevention Plan developed with the participant? ☐ Yes ☐ No → *Quit form*

2. Was the Prevention Plan developed by the participant alone or the couple?

   ☐ Participant alone ☐ The couple

3. Please describe the Prevention Plan elements agreed to by the participant/couple:

   a. The couple will have anal sex with each other ☐ Yes ☐ No ☐ Undecided ☐ Did not discuss

      1) The couple will use condoms when they have anal sex with each other:

      ☐ Never ☐ Sometimes ☐ Always ☐ Undecided

   b. The couple will have oral sex with each other ☐ Yes ☐ No ☐ Undecided ☐ Did not discuss

      1) The couple will use condoms when they have oral sex with each other:

      ☐ Never ☐ Sometimes ☐ Always ☐ Undecided

   c. The couple will have anal sex with outside partners ☐ Yes ☐ No ☐ Undecided ☐ Did not discuss

      1) The couple will use condoms when they have anal sex with outside partners:

      ☐ Never ☐ Sometimes ☐ Always ☐ Undecided

   d. The couple will have oral sex with outside partners ☐ Yes ☐ No ☐ Undecided ☐ Did not discuss

      1) The couple will use condoms when they have oral sex with outside partners:

      ☐ Never ☐ Sometimes ☐ Always ☐ Undecided

Reviewer's Initials ☐☐☐ F M S Date sent __/__/__ dd mm yy

DataFax # 027 Plate # 160 Visit # 40X

The Sibanye Health Project

# Non-Routine or Drop-in Visit Form #X



ⓘ This form should be completed by Study Staff to record instances of participants coming in for non-routine or unscheduled visits. The form does not need to be completed every time a participant comes to the office, only if he is receiving HIV or STI results, STI treatment, or accessing study services or information like those listed in section 1, question 2 below.

Study ID: ☐☐ - ☐☐☐☐

Staff initials: ☐☐☐ (*F M S*)

ID Verification Code: ☐☐☐☐

Date completed: ☐☐ ☐☐ 2 0 ☐☐ (*day month year*)

**Section 1: Services Provided**

1. Date of Visit: ☐☐ ☐☐ 2 0 ☐☐ (*day month year*)

2. Please indicate the services the participant is using or picking up today. *Tick all that apply.*

a. Return of HIV results ☐ → 1) Was referral to treatment and care provided? ☐ Yes ☐ No

b. Return of STI results ☐ → *Indicate below if STI medications dispensed.*

c. Dispensing of STI medication ☐ → *Complete* **STI Medication Dispensing Form**

d. Hepatitis B vaccination ☐ → *Complete* **Hepatitis B Vaccination Form**

e. Condoms ☐ → *Complete Section 2 on next page*

f. Lubricant ☐ → *Complete Section 3 on next page*

g. Individual HIV Counseling and Testing (VCT) ☐ → *Complete* **Drop-in HIV Testing, Counseling & Results Form**

h. PrEP refill ☐ → *Complete* **PrEP Return Visit & Adherence Form** *and* **PrEP Medication Dispensing Form**

i. PEP provision ☐ → *Complete* **PEP Provision Form**

j. Study-related information ☐ → *specify* ------------------------------------------------
*e.g. Participant asked about where to access STI treatment or mental health services.*

k. Other reason ☐ → *specify* ------------------------------------------------

ⓘ **If 2e or 2f ticked, please complete next page.**

Reviewer's Initials ☐☐☐ (*F M S*) Date sent __/__/__ (*dd mm yy*)



DRAFT

The Sibanye Health Project

# Non-Routine or Drop-in Visit Form #X



Study ID: ☐☐ - ☐☐☐☐

ID Verification Code: ☐☐☐☐

DRAFT

**Section 2: Condoms Distributed**

1. Please indicate which condom packages and how many were distributed to the participant. *Tick all that apply.*

| | Distributed? | Number of packages distributed |
|---|---|---|
| 1. Impulse Bare Pleasure | a. ☐ | b. ☐☐ |
| 2. Atlas Ultra-Thin | a. ☐ | b. ☐☐ |
| 3. Durex Enhanced Pleasure | a. ☐ | b. ☐☐ |
| 4. One Condom Pleasure Plus | a. ☐ | b. ☐☐ |
| 5. Trustex Assorted Flavors | a. ☐ | b. ☐☐ |
| 6. One Color Sensations | a. ☐ | b. ☐☐ |
| 7. Choice | a. ☐ | b. ☐☐ |
| 8. TheyFit | a. ☐ | b. ☐☐ |
| 9. Mixed Condom Package | a. ☐ | b. ☐☐ |
| 10. Other, describe: ------------------------------ | a. ☐ | b. ☐☐ |

**Section 3: Lubricant Distributed**

1. Please indicate which lubricant and how many were distributed to the participant. *Tick all that apply.*

| | Distributed? | Number of packages distributed |
|---|---|---|
| 1. Lifestyle Personal Lubricant | a. ☐ | b. ☐☐ |
| 2. ONE Move Lubricant | a. ☐ | b. ☐☐ |
| 3. Other, describe: ------------------------------ | a. ☐ | b. ☐☐ |

Reviewer's Initials ☐☐☐ (F M S) Date sent __/__/__ (dd mm yy)

The Sibanye Health Project

# Post-Exposure Prophylaxis (PEP) Provision Form #X



ⓘ This form should be completed by a provider every time a participant reports a potential PEP-eligible exposure. Please complete the **HIV Testing, Counseling and Results Form** before beginning this form.

Study ID: ☐☐ - ☐☐☐☐

Provider initials: ☐☐☐ (F M S)

ID Verification Code: ☐☐☐☐

Date completed: ☐☐ ☐☐ 2 0 ☐☐ (day month year)

**Section 1: Visit Information**

1. Current Visit:

☐ Baseline ☐ Month 1 ☐ Month 2 ☐ Month 3 ☐ Month 4 ☐ Month 5 ☐ Month 6
☐ Month 9 ☐ Month 12 ☐ Other (please specify) ------------------------

a. If other visit, indicate visit date: ☐☐ ☐☐ 2 0 ☐☐ (day month year)

**Section 2: PEP Eligibility Criteria**

1. PEP eligibility criteria:

| | Participant Eligible | |
|---|---|---|
| a. Exposure occured within the last 72 hours | ☐ Yes | ☐ No |
| b. Rapid HIV test(s) result at this visit is negative *(Reference Section 2 of the* **HIV Testing, Counseling and Results Form***)* | ☐ Yes | ☐ No |
| c. Exposure is identified as high-risk transmission by the provider | ☐ Yes | ☐ No |
| d. The exposure source is HIV positive or of unknown HIV status | ☐ Yes | ☐ No |
| e. Participant is willing to adhere to daily oral dosing | ☐ Yes | ☐ No |
| f. Participant has no contra-indications to PEP | ☐ Yes | ☐ No |
| g. Participant tested for Hepatitis B at baseline visit and received vaccine if susceptible. | ☐ Yes | ☐ No |

ⓘ *If items a-g under PEP Eligibility Criteria are marked 'yes' , the participant is eligible to initiate PEP.*

2. Is the participant eligible to initiate PEP? ☐ Yes ☐ No

Yes → *Continue to the next page.*
No → *Quit form*

Reviewer's Initials ☐☐☐ (F M S) Date sent __/__/__ (dd mm yy)



DRAFT

DataFax # 027 Plate # 171 Visit # 32X

The Sibanye Health Project

# Post-Exposure Prophylaxis (PEP) Provision Form #X



Study ID: ☐☐ - ☐☐☐☐

ID Verification Code: ☐☐☐☐

**Section 3: PEP Discussion and Acceptance**

ⓘ Before proceeding, discuss the PEP risks, side effects, and subsequent visits and contacts with the participant.

1. Was complete PEP information discussed with the participant? ☐ Yes ☐ No

2. Was PEP accepted by the participant?

☐ Yes ☐ No → a. Why not? (Tick all that apply)

- ☐ Too many visits
- ☐ Does not want side effects
- ☐ Not enough incentive
- ☐ Does not believe he is at risk
- ☐ No reason given
- ☐ Other: ______________________

3. Were referral procedures for the partner/source individual discussed with the participant, if applicable?

☐ Yes ☐ No

**Section 4: Required Specimen Collection**

1. All participants who initiate PEP must be tested for Hepatitis C. Was blood drawn for Hepatitis C testing?

☐ Yes ☐ No

2. All participants initiating PEP must have confirmatory testing (even if both rapid test results are negative, either ELISA or a tie-breaker rapid test should be completed). Was confirmatory testing completed?

☐ Yes ☐ No

**Section 5: Medication Dispensing and Adherence**

1. Was adherence counseling completed? ☐ Yes ☐ No

2. Were PEP bottles dispensed at this visit?

☐ Yes ☐ No → a. Why? ________________________________________________
________________________________________________

↓

b. How many bottles were dispensed? ☐☐

3. Bottles dispensed at this visit:

| | Kit Number | Bottle Number | Name of Medication | # of Tablets Dispensed |
|---|---|---|---|---|
| a. | 1) ☐☐☐☐ | 2) ☐☐ | 3) ________________________ | 4) ☐☐ |
| b. | 1) ☐☐☐☐ | 2) ☐☐ | 3) ________________________ | 4) ☐☐ |
| c. | 1) ☐☐☐☐ | 2) ☐☐ | 3) ________________________ | 4) ☐☐ |

4. Was first medication dose observed? ☐ Yes ☐ No

Reviewer's Initials ☐☐☐ (F M S) Date sent ___/___/___ (dd mm yy)



DRAFT

The Sibanye Health Project

# Condom Scorecard Form #X



ⓘ This form should be completed by Study Staff every time a participant completes and returns a Condom Scorecard. Transcribe information from the Condom Scorecard.

Study ID: ☐☐ - ☐☐☐☐

Staff initials: ☐☐☐ (F M S)

ID Verification Code: ☐☐☐☐

Date completed: ☐☐ (day) ☐☐ (month) 2 0 ☐☐ (year)

**Section 1: Scorecard Completion**

1. Visit Type (*tick only one*):

☐ Month 1 ☐ Month 2 ☐ Month 3 ☐ Month 4 ☐ Month 5 ☐ Month 6
☐ Month 9 ☐ Month 12 ☐ Other (please specify) ____________________

a. If other visit, indicate visit date: ☐☐ (day) ☐☐ (month) 2 0 ☐☐ (year)

2. Tick the visit number written on the ScoreCard, indicating when participant received Scorecard.

☐ Baseline ☐ Month 3 ☐ Other: ____________________

3. Tick the scenario that best explains when the participant completed Condom Scorecard.

☐ Participant completed card prior to study visit
☐ Participant completed card during the study visit
☐ Participant partially completed card prior to study visit; completed card at visit
☐ Other, specify____________________

**Section 2: Condom Use**

*Tick the boxes as indicated from* ***Section 1: Condom Use*** *on the Condom Scorecard.*

| | "I wore this condom *before* the study." | "I wore this condom *since I started* the study." | "I gave this condom to *my partner to wear*." |
|---|---|---|---|
| 1. Impulse Bare Pleasure | a. ☐ | b. ☐ | c. ☐ |
| 2. Atlas Ultra-Thin | a. ☐ | b. ☐ | c. ☐ |
| 3. Durex Enhanced Pleasure | a. ☐ | b. ☐ | c. ☐ |
| 4. One Condom Pleasure Plus | a. ☐ | b. ☐ | c. ☐ |
| 5. Trustex Assorted Flavors | a. ☐ | b. ☐ | c. ☐ |
| 6. One Color Sensations | a. ☐ | b. ☐ | c. ☐ |
| 7. Choice | a. ☐ | b. ☐ | c. ☐ |
| 8. TheyFit | a. ☐ | b. ☐ | c. ☐ |

Reviewer's Initials ☐☐☐ (F M S) Date sent __/__/__ (dd mm yy)



DRAFT

DataFax # 027 Plate # 201 Visit # 25X

The Sibanye Health Project

# Condom Scorecard Form #X



Study ID: ☐☐ - ☐☐☐☐

ID Verification Code: ☐☐☐☐

**Section 3: Condom Rating**

*Tick the boxes as indicated from **Section 2: Rating** on the Condom Scorecard.*

| | (very sad face) | (sad face) | (neutral face) | (smiling face) | (very happy face) | None selected |
|---|---|---|---|---|---|---|
| 1. Impulse Bare Pleasure | a. ☐ | b. ☐ | c. ☐ | d. ☐ | e. ☐ | f. ☐ |
| 2. Atlas Ultra-Thin | a. ☐ | b. ☐ | c. ☐ | d. ☐ | e. ☐ | f. ☐ |
| 3. Durex Enhanced Pleasure | a. ☐ | b. ☐ | c. ☐ | d. ☐ | e. ☐ | f. ☐ |
| 4. One Condom Pleasure Plus | a. ☐ | b. ☐ | c. ☐ | d. ☐ | e. ☐ | f. ☐ |
| 5. Trustex Assorted Flavors | a. ☐ | b. ☐ | c. ☐ | d. ☐ | e. ☐ | f. ☐ |
| 6. One Color Sensations | a. ☐ | b. ☐ | c. ☐ | d. ☐ | e. ☐ | f. ☐ |
| 7. Choice | a. ☐ | b. ☐ | c. ☐ | d. ☐ | e. ☐ | f. ☐ |
| 8. TheyFit | a. ☐ | b. ☐ | c. ☐ | d. ☐ | e. ☐ | f. ☐ |

DRAFT

Reviewer's Initials ☐☐☐ F M S Date sent ___/___/___ dd mm yy

The Sibanye Health Project

# Hepatitis B Vaccination Form: Dose X



To be completed by Study Staff each time a participant receives a injection as part of the Hepatitis B vaccination series, whether as part of a study visit or a drop-in visit.

Study ID: ☐☐ - ☐☐☐☐

Counselor initials: ☐☐☐ (F M S)

ID Verification Code: ☐☐☐☐

Date completed: ☐☐ (day) ☐☐ (month) 2 0 ☐☐ (year)

1. Visit Type (*tick only one*):

☐ Month 1 ☐ Month 2 ☐ Month 3 ☐ Month 4 ☐ Month 5 ☐ Month 6

☐ Month 9 ☐ Month 12 ☐ Other (please specify) ____________________

a. If other visit, indicate visit date: ☐☐ (day) ☐☐ (month) 2 0 ☐☐ (year)

2. Was injection succesfully administered?

☐ Yes ☐ No

a. If no, why not?

☐ Client refused ☐ Other, specify ____________________

DRAFT

Reviewer's Initials ☐☐☐ (F M S) Date sent ___/___/___ (dd mm yy)

The Sibanye Health Project

# Social Harms Reporting Form #X



ⓘ This form should be completed by the Counselor when a participant reports a social harm. Please complete a separate form for each social harm experienced by a participant.

Study ID: ☐☐ - ☐☐☐☐

Staff initials: ☐☐☐ (F M S)

ID Verification Code: ☐☐☐☐

Date completed: ☐☐ ☐☐ 2 0 ☐☐ (day month year)

1. Visit when the social harm was reported for the first time:
   - ☐ Baseline ☐ Month 1 ☐ Month 2 ☐ Month 3 ☐ Month 4 ☐ Month 5 ☐ Month 6
   - ☐ Month 9 ☐ Month 12 ☐ Other (please specify) ____________________

   a. If other visit, indicate visit date: ☐☐ ☐☐ 2 0 ☐☐ (day month year)

2. What type of social harm is this event? *(Tick all that apply)*
   - ☐ a. Family relationships *(negative experiences with family, excluding partner)*
   - ☐ b. Partner relationships *(negative experiences with significant other, spouse, or sex partner)*
   - ☐ c. Other personal relationships *(negative experiences with friends, neighbors, community members)*
   - ☐ d. Travel/Immigration *(problems obtaining permission to travel or enter another country, or problem with immigration/naturalization)*
   - ☐ e. Employment *(turned down for a job, lost a job, study visits interfering with work, other work problems)*
   - ☐ f. Education *(turned down by educational program, told to leave program, study visits interfering with school, other school problems)*
   - ☐ g. Medical/Dental *(refused medical or dental treatment, treated negatively by a provider)*
   - ☐ h. Housing *(trouble getting or keep housing, negative experiences with landlord, other housing problems)*
   - ☐ i. Other, specify: ____________________

3. Describe the social harm event: → a. Tick if participant declined to describe. ☐

   ____________________

   ____________________

   ____________________

4. Did this event involve physical harm to the participant? ☐ Yes ☐ No

5. Did this event involve physical or other harm to participant's child(ren)? ☐ Yes ☐ No

6. Date of social harm onset: ☐☐ ☐☐ 2 0 ☐☐ (day month year)

Reviewer's Initials ☐☐☐ (F M S) Date sent __/__/__ (dd mm yy)



DRAFT

The Sibanye Health Project

## Social Harms Reporting Form #X



Study ID: ☐☐ - ☐☐☐☐

ID Verification Code: ☐☐☐☐

7. Did this event include unwanted disclosure of study participation? Tick only one.
   - ☐ Yes, specify to who: ------------------------
   - ☐ Unknown/information not provided
   - ☐ No
   - ☐ Other, specify: ------------------------------

8. What impact did this situation have on the participant's quality of life? Tick only one.
   - ☐ No disturbance
   - ☐ A minimal disturbance that had no significant impact
   - ☐ A moderately upsetting disturbance, but did not have a significant impact
   - ☐ A major disturbance that had a significant impact
   - ☐ Other, specify: ------------------------------
   - ☐ Unknown/declined to answer

9. Describe what was done by staff and participant to address social harm.

   a. Participant: ------------------------------------------------------------

   b. Staff: ------------------------------------------------------------

10. Based on your discussion with the participant, what is the current status of the situation?
    - ☐ Resolved
    - ☐ Unable to resolve; no further action taken
    - ☐ Unresolved

    a. If either is marked, enter closure/resolution date: ☐☐ day ☐☐ month 20☐☐ year

    b. If unresolved, skip the date field above and scan this form to Emory. If and when the social harm is subsequently resolved, mark the box to the right and record the date resolved. Re-scan this page of the form.

    ☐ Mark if/when social harm subsequently resolved.

| Reviewer's Initials | Date sent |
|---|---|
| ☐☐☐ F M S | __/__/__ dd mm yy |



DRAFT

The *Sibanye* Health Project

# Adverse Event Form #X



ⓘ This form is to be completed by Study Staff each time an adverse event is experienced.

Study ID: ☐☐ - ☐☐☐☐

Study staff initials: ☐☐☐ (F M S)

ID Verification Code: ☐☐☐☐

Date completed: ☐☐ (day) ☐☐ (month) 2 0 ☐☐ (year)

**Section 1: Adverse Event Information**

1. Visit when the adverse event was reported for the first time:
   ☐ Baseline ☐ Month 1 ☐ Month 2 ☐ Month 3 ☐ Month 4 ☐ Month 5 ☐ Month 6
   ☐ Month 9 ☐ Month 12 ☐ Other (please specify) ____________________
   a. If other visit, indicate visit date: ☐☐ (day) ☐☐ (month) 2 0 ☐☐ (year)

2. Type of adverse event:
   Tick ('X') <u>ONLY</u> one of the following; if more than one adverse event was indicated at a visit, **complete a separate AE form for each adverse event**.
   ☐ a. nausea
   ☐ b. vomiting
   ☐ c. flatulence
   ☐ d. headache
   ☐ e. abdominal pain
   ☐ f. decreased weight/unintentional weight loss
   ☐ g. lactic acidosis
   ☐ h. hepatic steatosis
   ☐ i. steatohepatitis
   ☐ j. hypersensitivity reaction
   ☐ k. flares of hepatitis B
   ☐ l. renal toxicity
   ☐ m. metabolic complications
   ☐ n. other, *please describe:* ____________________

3. What is the severity of the adverse event?
   ☐ Mild ☐ Moderate ☐ Severe

4. Is the adverse event related to a study intervention? Tick 'X' <u>ONLY</u> one option
   ☐ Not related ☐ Probably not related
   ☐ Possibly related ☐ Probably related
   a. Which <u>study intervention</u> is the adverse event possibly/probably related to? *(Tick all that apply)*
   ☐ a. PrEP ☐ b. PEP ☐ c. Hepatitis B vaccination
   ☐ d. STI treatment ☐ e. Blood draw ☐ f. Condoms/lubricant
   ☐ g. Other, *please describe* ____________________

5. Is this a serious adverse event (SAE)? ☐ Yes ☐ No
   a. Which SAE criteria does it satisfy? *(Tick all that apply)*
   ☐ Results in death ☐ Is life-threatening
   ☐ Results in persistent of significant disability/incapacity
   ☐ Is a congenital anomaly/birth defect
   ☐ Requires inpatient hospitalization or prolongation of existing hospitalization

Reviewer's Initials ☐☐☐ (F M S) Date faxed __/__/__ (dd mm yy)



DRAFT

DataFax # 027 Plate # 216 Visit # 30X

The Sibanye Health Project

# Adverse Event Form #X



Study ID: ☐☐ - ☐☐☐☐

ID Verification Code: ☐☐☐☐

**Section 3: Adverse event resolution**

1. Has the adverse event been resolved?

☐ No ☐ Yes → *If yes, indicate date resolved.* ☐☐ ☐☐ 20☐☐
*day* *month* *year*

a. If no, skip the date field above and fax this form to Emory.

b. If and when the AE is subsequently resolved, mark the box to the right and record the date resolved in the box above. Amend the narrative in Section 4 indicating any treatment and the resolution of the AE. Re-fax this page of the form. → ☐ Mark if/when AE is subsequently resolved.

**Section 4: Narrative**

1. Please provide a brief, legible narrative of the AE, including any treatments provided and resolution, as applicable.

---

---

---

---

---

---

---

**Section 5: Reporting Requirements**

1. Does this AE need to be reported to DAIDS? ☐ No ☐ Yes → a. If yes, indicate date notified
☐☐ ☐☐ 20☐☐
*day* *month* *year*

2. Does this AE need to be reported to IRB? ☐ No ☐ Yes → a. If yes, indicate date notified
☐☐ ☐☐ 20☐☐
*day* *month* *year*

Reviewer's Initials ☐☐☐ *F M S* Date faxed ___/___/___ *dd mm yy*

DRAFT

DataFax # 027 Plate # 250 Visit # 42X

The Sibanye Health Project

# Early Study Stop Form #X



ⓘ This form is to be completed by Study Staff when a participant permanently discontinues from the study.

Study ID: ☐☐ - ☐☐☐☐

Provider initials: ☐☐☐ (F M S)

ID Verification Code: ☐☐☐☐

Date completed: ☐☐ ☐☐ 20☐☐ (day month year)

**Section 1: Reason for Study Stop**

1. Please indicate the one (primary) reason that participant is no longer in the study and answer any relevant questions.

☐ **HIV-Positive at baseline, not followed prospectively**
*(HIV+ men enrolled over 20% threshold)*
1. Enter date participant notified of study discontinuation into **Section 2, Q1.**
2. Tick Baseline under **Section 2, Q2.**
3. Enter date of Baseline visit into **Section 2, Q3.**

☐ **Moved out of study city**
1. Enter date staff learned of move into **Section 2, Q1**.
2. Tick box indicating participant's last visit attended under **Section 2, Q2.**
3. Enter date of participant's last attended visit into **Section 2, Q3**.
4. Document circumstances in **Section 3.**

☐ **Admitted to health care facility**
1. Enter date staff learned of admission in **Section 2, Q1**.
2. Tick box indicating participant's last visit attended under **Section 2, Q2.**
3. Enter date of participant's last attended visit into **Section 2, Q3**.
4. Document circumstances in **Section 3.**

☐ **Lost to follow-up**
1. Enter date participant declared lost to follow-up into **Section 2, Q1**.
2. Tick box indicating participant's last visit attended under **Section 2, Q2.**
3. Enter date of participant's last attended visit into **Section 2, Q3**.
4. Document circumstances & retention efforts in **Section 3**.

☐ **Voluntary withdrawal**
1. Please indicate primary reason for voluntary withdrawal:
   - ☐ Time conflict
   - ☐ Time commitment
   - ☐ Inconvenient venues
   - ☐ Discomfort with study procedures
   - ☐ Transportation
   - ☐ Had bad experience
   - ☐ Other, describe in **Section 3.**
2. Enter date participant withdrew (notified staff of withdrawal) into **Section 2, Q1**.
3. Tick box indicating participant's last visit attended under **Section 2, Q2.**
4. Enter date of participant's last attended visit into **Section 2, Q3**.
5. Document circumstances in **Section 3.**

☐ **Inappropriate enrollment**
1. Enter date participant notified of study discontinuation into **Section 2, Q1**.
2. Tick box indicating participant's last visit attended under **Section 2, Q2.**
3. Enter date of participant's last attended visit into **Section 2, Q3**.
4. Document circumstances in **Section 3.**

☐ **Administrative discontinuation**
1. Enter date participant notified of study discontinuation into **Section 2, Q1**.
2. Tick box indicating participant's last attended visit under **Section 2, Q2.**
3. Enter date of participant's last attended visit into **Section 2, Q3.**
4. Document circumstances in **Section 3**.

☐ **Incarceration**
1. Information source (part., relative, newspaper, etc.): ____________________
2. Enter date staff learned of incarceration into **Section 2, Q1**.
3. Tick box indicating participant's last attended visit under **Section 2, Q2.**
4. Enter date of participant's last attended visit into **Section 2, Q3.**

☐ **Deceased**
1. Suspected cause of death: ____________________
2. Information source (part., relative, newspaper, etc.): ____________________
3. Enter date of death into **Section 2, Q1**.
4. Tick box indicating participant's last attended visit under **Section 2, Q2.**
5. Enter date of participant's last attended visit into **Section 2, Q3**.

Reviewer's Initials ☐☐☐ (F M S) Date faxed __/__/__ (dd mm yy)



DRAFT

The Sibanye Health Project

# Early Study Stop Form #X



Study ID: ☐☐ - ☐☐☐☐

ID Verification Code: ☐☐☐☐

## Section 2: Timing of Study Stop

1. Study stop date: ☐☐ ☐☐ 20☐☐
   day month year

2. Last visit attended:
   ☐ Baseline ☐ Month 1 ☐ Month 2 ☐ Month 3 ☐ Month 4 ☐ Month 5
   ☐ Month 6 ☐ Month 9 ☐ Month 12 ☐ Other: ____________________________

3. Date of last visit attended: ☐☐ ☐☐ 20☐☐
   day month year

## Section 3: Additional Information

*If indicated in Section 1, briefly explain the circumstances of early study stop below.*

_______________________________________________

_______________________________________________

_______________________________________________

_______________________________________________

_______________________________________________

_______________________________________________

_______________________________________________

_______________________________________________

DRAFT



Reviewer's Initials ☐☐☐ (F M S) Date faxed ___/___/___ (dd mm yy)

The Sibanye Health Project

# Restart Form #X



ⓘ This form should be completed by Study Staff for participants who return to the study after a study stop.

Study ID: ☐☐ - ☐☐☐☐

Staff initials: ☐☐☐ (F M S)

ID Verification Code: ☐☐☐☐

Date completed: ☐☐ ☐☐ 2 0 ☐☐ (day month year)

1. What was the date of participant's return to study? ☐☐ ☐☐ 2 0 ☐☐ (day month year)

2. Please indicate the reasons for return to the study: (tick all that apply)
   - ☐ Recontacted
   - ☐ Relocated
   - ☐ Can travel
   - ☐ Time conflict resolved
   - ☐ Transportation issues resolved
   - ☐ Released from incarceration
   - ☐ Unknown
   - ☐ Other, specify ____________________

3. Explain circumstances: ____________________

ⓘ If participant has returned to study, make sure all contact information has been updated.

4. Comments: ____________________

Reviewer's Initials ☐☐☐ (F M S) Date sent __/__/__ (dd mm yy)



DRAFT

The Sibanye Health Project

# Missed Visit Form - X Month



ⓘ Complete this form each time a participant misses a required visit according to the visit window outlined in the Study-specific Procedures (SSP) manual.

Study ID: ☐☐ - ☐☐☐☐

Staff initials: ☐☐☐ (*F M S*)

ID Verification Code: ☐☐☐☐

Date completed: ☐☐ ☐☐ 2 0 ☐☐ (*day month year*)

1. Target Visit Date : ☐☐ ☐☐ 2 0 ☐☐ (*day month year*)

2. Reason visit was missed. (*Tick only one)*

- ☐ Unable to contact participant
- ☐ Unable to schedule appointment(s) within allowable window period
- ☐ Participant had transportation difficulties
- ☐ Participant refused visit
- ☐ Participant admitted to a health care facility → complete **Study Stop Form** if lasting for duration of the study
- ☐ Participant incarcerated → complete **Study Stop Form** if lasting for duration of the study
- ☐ Participant moved from study city → complete **Study Stop Form** if lasting for duration of the study
- ☐ Participant withdrew from the study → complete **Study Stop Form**
- ☐ Participant deceased → complete **Study Stop Form**
- ☐ Don't know
- ☐ Other, specify ----------------------------------------

3. Steps taken to address continued participation and the importance of attending subsequent study visits, for example updating contact information or discussing transportation options:

----------------------------------------

----------------------------------------

----------------------------------------

----------------------------------------

ⓘ If participant misses a PrEP monitoring/follow-up visit, you may need to complete the **PrEP Medication Discontinuation Form**.

Reviewer's Initials ☐☐☐ (*F M S*) Date sent ___/___/___ (*dd mm yy*)



DRAFT

The Sibanye Health Project

# Participant Transfer Form #X



This is an administrative only form. Emory staff will complete this form when a participant moves from one study city to the other and wants to continue their study participation in the new city.

Study ID: ☐☐ - ☐☐☐☐

Staff initials: ☐☐☐ (F M S)

ID Verification Code: ☐☐☐☐

Date completed: ☐☐ (day) ☐☐ (month) 2 0 ☐☐ (year)

1. Origin site #: ☐
   1 = Cape Town
   2 = Port Elizabeth

2. Transfer site #: ☐
   1 = Cape Town
   2 = Port Elizabeth

3. Effective date of transfer: ☐☐ (day) ☐☐ (month) 2 0 ☐☐ (year)

DRAFT

Reviewer's Initials ☐☐☐ (F M S) Date sent __/__/__ (dd mm yy)

DataFax # 027 Plate # 275 Visit # 371

The Sibanye Health Project

# Participant-Specific Protocol Deviation Form #X



ⓘ To be used to document protocol deviations each time they occur related to one participant. Complete this form within one week of the event occurring. Complete a separate form for each protocol deviation.

Study ID: ☐☐ - ☐☐☐☐

Staff initials: ☐☐☐ (F M S)

ID Verification Code: ☐☐☐☐

Date completed: ☐☐ ☐☐ 20☐☐ (day month year)

**Section 1: Description of Protocol Deviation**

1. Deviation Category: *(Tick only one)*

☐ Safety ☐ Eligibility ☐ Other, specify: ______________________

☐ Informed Consent ☐ Protocol implementation ______________________

2. Deviation Code: *(Tick only one that corresponds with the deviation category)*

***Safety Category:***

☐ Not reporting a SAE within 24 hours ☐ Laboratory tests not done

☐ AE/SAE not reported to IRB ☐ Other, specify: ______________________

***Informed Consent Category:***

☐ Failure to obtain informed consent

☐ Consent form used was not current IRB-approved version

☐ Consent form does not include updates/information required by IRB

☐ Consent form not signed & dated by participant

☐ Consent form does not contain all required signatures

☐ Other, specify: ______________________

***Eligibility Category:***

☐ Participant did not meet eligibility criteria ☐ Treatment of participant prior to IRB approval of protocol

☐ Other, specify: ______________________

***Protocol Implementation Category:***

☐ Incorrect lab tests done ☐ Use of unallowable concomitant treatments

☐ Participant receives wrong treatment/dosage ☐ Missed assessment

☐ Other, specify: ______________________

3. Date deviation first occurred: ☐☐ ☐☐ 20☐☐ (day month year)

4. Date deviation ended, applicable if occurring over a period of time: ☐☐ ☐☐ 20☐☐ (day month year)

5. Provide a description of the protocol deviation:

______________________

______________________

______________________

Reviewer's Initials ☐☐☐ (F M S) Date sent ___/___/___ (dd mm yy)



DRAFT

The Sibanye Health Project

# Participant-Specific Protocol Deviation Form #X



Study ID: [ ][ ] - [ ][ ][ ][ ]

ID Verification Code: [ ][ ][ ][ ]

**Section 2: Steps to Correct Protocol Deviation**

1. Describe plans and/or actions taken to address the deviation:

------------------------------------------------------------

------------------------------------------------------------

------------------------------------------------------------

------------------------------------------------------------

2. Describe plans and/or actions taken to prevent future occurrences of the deviation:

------------------------------------------------------------

------------------------------------------------------------

------------------------------------------------------------

------------------------------------------------------------

**Section 3: Site Reporting of Protocol Deviation**

1. Does site IRB need to be notified of the deviation? ☐ Yes ☐ No

a. Date IRB notified: [ ][ ] [ ][ ] [2][0][ ][ ]
*day* *month* *year*

DRAFT

Reviewer's Initials Date sent
[ ][ ][ ] __/__/__
*F M S* *dd mm yy*

The Sibanye Health Project

# Participant-Specific Protocol Deviation Form #X



This page must be completed by Emory University staff each time the sites complete the first two pages of this form.

Study ID: ☐☐ - ☐☐☐☐

Emory staff initials: ☐☐☐ (F M S)

ID Verification Code: ☐☐☐☐

Date completed: ☐☐ (day) ☐☐ (month) 20☐☐ (year)

**Section 4: Emory Reporting of Protocol Deviation**

*In order for a protocol deviation to be reportable to the IRB, it must be a substantive change from the protocol and adverse effect at least one of the following:*

- *The rights, welfare, or safety of the subjects;*
- *The integrity of the research data;*
- *The subjects' willingness to continue participation.*

1. Does IRB need to be notified of the deviation? ☐ Yes ☐ No

   a. Date IRB notified: ☐☐ (day) ☐☐ (month) 20☐☐ (year)

2. Was DAIDS notified of the deviation? ☐ Yes ☐ No

   a. Date DAIDS notified: ☐☐ (day) ☐☐ (month) 20☐☐ (year)

3. Does the deviation require a revision of the protocol and/or consent forms? ☐ Yes ☐ No

4. Do subjects need to be notified of this protocol deviation? ☐ Yes ☐ No

DRAFT

Reviewer's Initials ☐☐☐ (F M S) Date sent __/__/__ (dd mm yy)

The Sibanye Health Project

# Generic Protocol Deviation Form #X



ⓘ To be used to document non participant-specific protocol deviations each time they occur. Generic protocol deviations do not relate to one participant, but instead impact overall implementation of the study. Complete this form within one week of the event occurring. Complete a separate form for each protocol deviation.

Generic ID: XX - XXXX

Staff initials: ☐☐☐ (F M S)

Date completed: ☐☐ ☐☐ 20☐☐ (day month year)

**Section 1: Description of Protocol Deviation**

1. Deviation Type: *(Tick only one)*
   - ☐ Possible breach of participant information (ex. computer not properly password-protected, files not locked)
   - ☐ Equipment failure
   - ☐ Shortage of medication, specimen collection or lab materials
   - ☐ Logs not properly updated (ex. referral list out of date, pharmacy logs not updated)
   - ☐ Change in protocol without IRB/PSRC approval
   - ☐ Site or staff operating without appropriate certification/regulartory approval
   - ☐ Other, specify: ______________________________

2. Date deviation first occurred:

   ☐☐ ☐☐ 20☐☐ (day month year)

3. Date deviation ended, applicable if occurring over a period of time:

   ☐☐ ☐☐ 20☐☐ (day month year)

4. Provide a description of the protocol deviation:

______________________________

______________________________

______________________________

______________________________

______________________________

______________________________

______________________________

Reviewer's Initials ☐☐☐ (F M S) Date sent __/__/__ (dd mm yy)



DRAFT

# Generic Protocol Deviation Form #X



Generic ID: X X - X X X X

**Section 2: Steps to Correct Protocol Deviation**

1. Describe plans and/or actions taken to address the deviation:

----------------------------------------------------------------

----------------------------------------------------------------

----------------------------------------------------------------

----------------------------------------------------------------

2. Describe plans and/or actions taken to prevent future occurrences of the deviation:

----------------------------------------------------------------

----------------------------------------------------------------

----------------------------------------------------------------

----------------------------------------------------------------

**Section 3: Site Reporting of Protocol Deviation**

1. Does site IRB need to be notified of the deviation? ☐ Yes ☐ No

   a. Date IRB notified: ☐☐ ☐☐ 2 0 ☐☐

   *day* *month* *year*

DRAFT



Reviewer's Initials Date sent

☐☐☐ __/__/__

*F M S* *dd mm yy*

The Sibanye Health Project

# Generic Protocol Deviation Form #X



This page must be completed by Emory University staff each time the sites complete the first two pages of this form.

Generic ID: XX - XXXX

Emory staff initials: ☐☐☐ (F M S)

Date completed: ☐☐ ☐☐ 20☐☐ (day month year)

DRAFT

**Section 4: Emory Reporting of Protocol Deviation**

*In order for a protocol deviation to be reportable to the IRB, it must be a substantive change from the protocol and adverse effect at least one of the following:*

- *The rights, welfare, or safety of the subjects;*
- *The integrity of the research data;*
- *The subjects' willingness to continue participation.*

1. Does IRB need to be notified of the deviation? ☐ Yes ☐ No

   a. Date IRB notified: ☐☐ ☐☐ 20☐☐ (day month year)

2. Was DAIDS notified of the deviation? ☐ Yes ☐ No

   a. Date DAIDS notified: ☐☐ ☐☐ 20☐☐ (day month year)

3. Does the deviation require a revision of the protocol and/or consent forms? ☐ Yes ☐ No

4. Do subjects need to be notified of this protocol deviation? ☐ Yes ☐ No

Reviewer's Initials ☐☐☐ (F M S) Date sent __/__/__ (dd mm yy)
