# Supplementary material for: Sibanye Methods for Prevention Packages Program Project Protocol: Pilot Study of HIV Prevention Interventions for Men Who Have Sex With Men in South Africa
Source: JMIR Res Protoc. 2014 Oct 16;3(4):e55. doi: 10.2196/resprot.3737 (PMC4210958; doi:10.2196/resprot.3737)
Supplement: Supplementary file 9 [file resprot_v3i4e55_app9.pdf]

Sibanye Health Project Baseline Survey

Select Language

At this time, please select your preferred language using the language bar at the top of the page.

You will not be able to change languages later in the survey.

Current Language: **English**

Introduction

Thank you for taking our survey today.

Helpful tips:

- Questions marked with a red asterisk (*) are required questions that you must answer to move forward.
- This is a **forward-only** survey. When you finish a page, proceed to the next page by clicking the "Next" button. You may not go backwards to pages you already complete. Please don't use the back button on your browser.

Your confidentiality is important to us! All information you provide today in this survey will be held confidentially. Your answers will not be shared with the counselors or providers. Your answers will be used only for research purposes and we do not collect information to identify you and link you to your answers. Researchers will only see data that is anonymous.

Population Group/Language

Please tell us a little bit about yourself.

How would you describe yourself?

( ) Black African
( ) Coloured
( ) Indian or Asian
( ) White
( ) Other, please specify: ____

Which language do you speak most often?

( ) Afrikaans
( ) English
( ) IsiNdebele

( ) IsiXhosa
( ) IsiZulu
( ) Sepedi
( ) Sesotho
( ) Setswana
( ) Sign language
( ) SiSwati
( ) Tshivenda
( ) Xitsonga
( ) Other, please specify: ____

Are you a South African citizen?
( ) Yes
( ) No

Sexual Orientation

Do you think of yourself as:
( ) Heterosexual or Straight
( ) Homosexual or Gay
( ) Bisexual
( ) Other, please specify: ____

How do you currently identify yourself?
( ) Male
( ) Female
( ) Transgender
( ) Other: ____

Are you married?
( ) Yes, to a man
( ) Yes, to a woman
( ) Not married

Logic: Dynamically shown if "Are you married?" = Yes, to a man or "Are you married?" = Yes, to a woman

Are you currently living with your spouse?
( ) Yes
( ) No

Employment/Student

What is the highest level in school that you completed?
( ) No formal education
( ) Some primary school, never went to high school
( ) Some high school, but didn't pass Grade 12 or Matric
( ) Matric / Passed Grade 12
( ) Diploma - National Qualifications Framework (NQF)
( ) University - undergraduate
( ) University - postgraduate

How would you describe your current work situation?
( ) Full time paid job (40 hours a week or more)
( ) Part time paid job (less than 40 hours a week)
( ) Unemployed

Are you a student?
( ) Full time student
( ) Part time student
( ) Not a student

What was your annual household income last year from all sources before taxes?
( ) No income
( ) R1 – R4,800
( ) R4,801 – R9,600
( ) R9,601 – R19,200
( ) R19,201 – R38,400
( ) R38,401 – R76,800
( ) R76,801 – R153,600
( ) R153,601 – R307,200
( ) R307,201 or more

Including yourself, how many people depended on this income?
Drop-down: 1-99

Living Situation

Which of the following best describes the type of dwelling or housing structure you currently live in?
( ) House or brick structure on a separate stand or yard
( ) Traditional dwelling or hut made of traditional materials
( ) Flat in a block of flats

( ) Town or semi-detached house (simplex, duplex or triplex)
( ) House or room, in backyard
( ) Informal dwelling or shack, in backyard
( ) Informal dwelling or shack, NOT in backyard for example in an informal or squatter settlement
( ) Room/flatlet on a property or a larger dwelling/servants' quarters/granny flat
( ) Caravan or tent
( ) Living in the streets, homeless
( ) Other, please specify: ____

How long have you lived or stayed at the place you currently live in?
( ) Less than one year
( ) 1-3 years
( ) 4-6 years
( ) 7-10 years
( ) More than 10 years

What is the postal code for the place you currently live?
____

[ ] Don't know the postal code

**Logic: If homeless not selected above**

In the past 12 months (since [question("value"), id="1778"]), have you been homeless at any time? By homeless, I mean you were living on the street, in a shelter, temporarily staying with friends or relatives, or living in a car.
( ) Yes
( ) No

Medical Aid or Benefit Scheme

Are you covered by a Medical Aid or Medical Benefit Scheme or any scheme that helps you pay for health-care or drug services?
( ) Yes
( ) No

LGBT Interactions

In the past 12 months (since [question("value"), id="1778"]), have you had any interactions with organizations that support gay or other men who have sex with men?

( ) Yes
( ) No

Logic: Dynamically shown if "In the past 12 months (since [question("value"), id="1778"]), have you had any interactions with organizations that support gay or other men who have sex with men?" = Yes

What kind of interactions did you have with organizations that support gay or other men who have sex with men? (Tick all that apply)

[ ] I attended events
[ ] I got condoms
[ ] I got lube
[ ] I attended support groups
[ ] I am a volunteer or ambassador
[ ] I received HIV testing and counseling
[ ] Other, please specify:: ____

HIV Knowledge

The next set of questions asks about your HIV knowledge. For each statement, click "True", "False" or "Don't know". If you do not know, please do not guess. Instead, please click the button "Don't know".

	True	False	Don't know
Coughing and sneezing DO NOT spread HIV.	( )	( )	( )
A person can get HIV by sharing a glass of water with someone who has HIV.	( )	( )	( )
Pulling out the penis before a man climaxes/cums keeps his partner from getting HIV during sex.	( )	( )	( )
A woman can get HIV if she has anal sex with a man.	( )	( )	( )
Showering or washing one’s genitals / private parts after sex keeps a person from getting HIV.	( )	( )	( )
All pregnant women infected with HIV will have babies born with AIDS.	( )	( )	( )

People who have been infected with HIV quickly show serious signs of being infected.	( )	( )	( )
There is a vaccine that can stop adults from getting HIV.	( )	( )	( )
People are likely to get HIV by deep kissing (putting their tongue in their partner's mouth), if their partner has HIV.	( )	( )	( )
A woman cannot get HIV if she has sex during her period.	( )	( )	( )
There is a female condom that can help decrease a woman’s chance of getting HIV.	( )	( )	( )
A person will NOT get HIV if they are taking antibiotics.	( )	( )	( )
Having sex with more than one partner can increase a person’s chance of becoming infected with HIV.	( )	( )	( )
Taking a test for HIV one week after having sex will tell a person if she or he has HIV.	( )	( )	( )
A person can get HIV by sitting in a hot tub or a swimming pool with a person who has HIV.	( )	( )	( )
A person can get HIV from oral sex.	( )	( )	( )
Using Vaseline or baby oil with condoms lowers the chance of getting HIV.	( )	( )	( )
A person is more likely to get HIV from vaginal sex than from oral sex.	( )	( )	( )
A person is more likely to get HIV from anal sex than from vaginal sex.	( )	( )	( )
Nearly all HIV transmission comes from having lots of boyfriends or hook-ups.	( )	( )	( )
A person is more likely to get HIV from receptive sex (bottom) than insertive sex (top)	( )	( )	( )

Medical Care Utilization

When was the last time you went to a doctor or nurse for a medical issue? This does NOT include visits to traditional healers such as Sangomas or Faith healers.

[ ] I've never been to a doctor or nurse for a medical issue

Month
Drop-down: Don't know, January-December

Year
Drop-down: Don't know, 2014-2000, Before 2000

LGBT sensitization

In the past 12 months, which reasons did you have for visiting a doctor or nurse for a medical issue? (Tick all that apply)
[ ] HIV care
[ ] HIV testing
[ ] Routine care
[ ] Treatment for an injury
[ ] Treatment for an illness
[ ] Mental health
[ ] Other, please specify: ____

In the past 12 months, have you told any health care providers that you have sex with men?
( ) No, they didn't ask
( ) No, they asked but I didn't tell them
( ) Yes, they asked
( ) Yes, they didn't ask but I told them

Logic: Dynamically shown if "In the past 12 months, have you told any health care providers that you have sex with men?" = No, they didn't ask or "In the past 12 months, have you told any health care providers that you have sex with men?" = No, they asked but I didn't tell them

Why didn't you tell them? (Tick all that apply)
[ ] I thought the health care provider would make fun of me or treat me differently
[ ] I thought the health care provider would refuse to provide appropriate care for me
[ ] I was uncomfortable talking about having sex with men with my health care provider
[ ] I was uncomfortable talking about sex at all with my health care provider
[ ] I thought friends, family or other people in the community would find out

[ ] I thought it was not important to tell my health care provider

Logic: Dynamically shown if "In the past 12 months, have you told any health care providers that you have sex with men?" = Yes, they asked or "In the past 12 months, have you told any health care providers that you have sex with men?" = Yes, they didn't ask but I told them

After confirming or revealing that you have sex with other men, did the following happen? (Tick all that apply)

[ ] The health care provider offered counseling on safer practices while having sex with men
[ ] The health care provider avoided you
[ ] The health care provider made fun of you or treated you differently
[ ] The health care provider refused to help you
[ ] The health care provider offered counseling on how to prevent HIV
[ ] The health care provider asked you about anal itching, anal sores
[ ] The health care provider avoided performing certain screening tests (such as chlamydia or HIV screening) because they felt uncomfortable
[ ] The health care provider offered testing for HIV and STIs
[ ] The health care provider suggested I get care at another place with better services for gay or other men who have sex with men

STI Testing History

Have you ever been tested for any sexually transmitted infections (STIs)? These are infections that you might have gotten from having sex with someone, such as gonorrhea or chlamydia.

( ) Yes
( ) No
( ) Don't know

Logic: Dynamically shown if "Have you ever been tested for any sexually transmitted infections (STIs)? These are infections that you might have gotten from having sex with someone, such as gonorrhea or chlamydia." = Yes

In what month and year did you have your most recent STI test?

Month

Drop-down: Don't know, January-December

Year

Drop-down: Don't know, 2014-2000, Before 2000

Logic: Dynamically shown if "Have you ever been tested for any sexually transmitted infections (STIs)? These are infections that you might have gotten from having sex with someone, such as gonorrhea or chlamydia." = Yes

In the past 12 months, has a doctor or nurse told you that you have any of the following sexually transmitted infections? (Tick all that apply)

[ ] Gonorrhea
[ ] Chlamydia
[ ] Herpes
[ ] Syphilis
[ ] None of these

Logic: Dynamically shown if "Have you ever been tested for any sexually transmitted infections (STIs)? These are infections that you might have gotten from having sex with someone, such as gonorrhea or chlamydia." = No or "Have you ever been tested for any sexually transmitted infections (STIs)? These are infections that you might have gotten from having sex with someone, such as gonorrhea or chlamydia." = Don't know

Even though you've never been tested for STIs, in the past 12 months, has a doctor or nurse told you that you have any of the following sexually transmitted infections? (Tick all that apply)

[ ] Gonorrhea
[ ] Chlamydia
[ ] Herpes
[ ] Syphilis
[ ] None of these

Have you ever had rectal STI testing (a swab from your anus)?

( ) Yes
( ) No
( ) Don't know

HIV Testing History

Have you ever been tested for HIV?*

( ) Yes
( ) No
( ) Don't know

Logic: Dynamically shown if "Have you ever been tested for HIV?" = Yes

How many HIV tests have you had in your lifetime?
( ) Don't know
( ) 1
( ) 2
( ) 3
( ) 4
( ) 5
( ) 6 or more

Logic: Dynamically shown if "Have you ever been tested for HIV?" = No

What are the reasons you have not been tested for HIV? (Tick all that apply)
[ ] You haven't done anything to get HIV
[ ] You were afraid of finding out that you have HIV
[ ] You don't know where to go to get tested
[ ] You couldn't get transportation to a testing place
[ ] You don't like needles
[ ] You were worried your name would be reported to the government if you tested positive
[ ] You were worried someone would find out about your test results
[ ] You were afraid of losing your job, housing, family or friends if people found out you tested positive
[ ] You didn't have time
[ ] You didn't have the money to pay for the test
[ ] Other, describe:: ____

No HIV Testing - most important reason

You selected the following reasons you have not been tested for HIV. Which reason is the most important?

Page entry logic: This page will show when: Question #37 contains any ("Yes")

HIV Testing

Logic: Dynamically shown if "Have you ever been tested for HIV?" = Yes

In what month and year did you have your ***most recent*** HIV test?

Month
Drop-down: Don't know, January-December

Year
Drop-down: Don't know, 2014-2000, Before 2000

Logic: Dynamically shown if "Have you ever been tested for HIV?" = Yes

What are the reasons you had your *most recent* HIV test? (Tick all that apply)
[ ] Experienced symptoms that could indicate HIV infection
[ ] As part of regular testing (such as every 6 months or every year)
[ ] To share my results with my partner(s) (so we could have sex without condoms)
[ ] As part of a committed relationship with an HIV positive partner
[ ] After sex with a partner I now suspect to be HIV positive
[ ] After sex with a partner of unknown HIV status
[ ] As part of a circumcision process, which required an HIV test
[ ] I was afraid after I had sex (vaginal or anal) for the first time
[ ] My doctor recommended it
[ ] Some other reason, please specify:: ____

Where did you get your *most recent* HIV test?
( ) Community health center or clinic
( ) Stand-alone HIV counseling and testing center (VCT)
( ) Mobile unit (like Tutu Tester)/event
( ) Private doctor's office
( ) Hospital or emergency room
( ) At home
( ) Other, please specify:: ____

HIV Testing

In what month and year did you have your ***second most recent*** HIV test?

Month
Drop-down: Don't know, January-December

Year
Drop-down: Don't know, 2014-2000, Before 2000

What are the reasons you had your *second most recent* HIV test? (Tick all that apply)
[ ] Experienced symptoms that could indicate HIV infection

[ ] As part of regular testing (such as every 6 months or every year)
[ ] To share my results with my partner(s) (so we could have sex without condoms)
[ ] As part of a committed relationship with an HIV positive partner
[ ] After sex with a partner I now suspect to be HIV positive
[ ] After sex with a partner of unknown HIV status
[ ] As part of a circumcision process, which required an HIV test
[ ] I was afraid after I had sex (vaginal or anal) for the first time
[ ] My doctor recommended it
[ ] Some other reason, please specify:: ____

Where did you get your *second most recent* HIV test?
( ) Community health center or clinic
( ) Stand-alone HIV counseling and testing center (VCT)
( ) Mobile unit (like Tutu Tester)/event
( ) Private doctor's office
( ) Hospital or emergency room
( ) At home
( ) Other, please specify:: ____

HIV Testing

In what month and year did you have your ***third most recent*** HIV test?

Month
Drop-down: Don't know, January-December

Year
Drop-down: Don't know, 2014-2000, Before 2000

What are the reasons you had your *third most recent* HIV test? (Tick all that apply)
[ ] Experienced symptoms that could indicate HIV infection
[ ] As part of regular testing (such as every 6 months or every year)
[ ] To share my results with my partner(s) (so we could have sex without condoms)
[ ] As part of a committed relationship with an HIV positive partner
[ ] After sex with a partner I now suspect to be HIV positive
[ ] After sex with a partner of unknown HIV status
[ ] As part of a circumcision process, which required an HIV test
[ ] I was afraid after I had sex (vaginal or anal) for the first time
[ ] My doctor recommended it
[ ] Some other reason, please specify:: ____

Where did you get your *third most recent* HIV test?
( ) Community health center or clinic
( ) Stand-alone HIV counseling and testing center (VCT)
( ) Mobile unit (like Tutu Tester)/event
( ) Private doctor's office
( ) Hospital or emergency room
( ) At home
( ) Other, please specify:: ____

**Page entry logic:** If ever tested

HIV Diagnosis History

What was the result of your most recent HIV test?*
( ) Negative
( ) Positive
( ) Indeterminant or Inconclusive
( ) Didn't get the results of my last HIV test

**Logic: If tested positive**

Was this your first HIV-positive diagnosis?
( ) Yes
( ) No

**Logic: If did not test positive**

Have you ever gotten a positive test result for HIV?*
( ) Yes
( ) No

**Logic: If ever tested positive**

When was the month and year of your ***first*** HIV diagnosis?
Month
Drop-down: Don't know, January-December

Year
Drop-down: Don't know, 2014-2000, Before 2000

Page entry logic: If HIV-positive

HIV Care: General

Have you ever gone to a doctor's office or clinic for HIV care (like CD4 tests, viral load tests, or prescriptions for HIV medicines)?

( ) Yes
( ) No

Logic: If no

What are the reasons you haven't visited a doctor, nurse, or other health care worker for HIV medical care since you have been diagnosed? (Tick all that apply)

[ ] I just got diagnosed and haven't had time to get care
[ ] I don't have health insurance
[ ] I don't know where to get HIV care
[ ] There are no HIV doctors in my area
[ ] I am afraid doctors will treat me differently because I have sex with men
[ ] I am afraid doctors will treat me differently because I have HIV
[ ] I don't think it's necessary
[ ] I thought it was necessary, but never tried to get care
[ ] It would be difficult to take time off work to get HIV care
[ ] I don't want to think about my HIV status
[ ] I don't want anyone to know about my HIV status
[ ] I don't feel sick
[ ] I didn’t have needs other than CD4 and viral load monitoring
[ ] Other, please specify: ____

Logic: If yes

How long after your first diagnosis did you go to receive HIV care?

( ) Within 3 months
( ) 3 months or more, but less than 6 months
( ) 6 months or more, but less than 12 months
( ) 12 months or more

Logic: If yes

In the past 12 months (since [question("value"), id="1778"]), have you gone to a doctor's office or clinic for HIV care (like CD4 tests, viral load tests, or prescriptions for HIV medicines)?

( ) Yes
( ) No

Reasons no HIV doctor

You selected the following reasons you have not visited a doctor, nurse of other health care worker for HIV medical care. Which reason is the most important?

**Page entry logic:** If HIV-positive

HIV Care Location

In the past 12 months (since [question("value"), id="1778"]), in which of the following cities have you gotten HIV care? (Tick all that apply)

[ ] Cape Town
[ ] Port Elizabeth
[ ] East London
[ ] Other, please specify: ____

In the past 12 months (since [question("value"), id="1778"]), has there been one place where you go for most of your HIV care?

( ) Yes
( ) No

**Logic: If no**

What was the main reason you didn't have a usual place to get HIV care during the past 12 months (since [question("value"), id="1778"])?

( ) Couldn't afford a usual source of HIV care
( ) Didn't know to find a usual source of HIV care
( ) Couldn't get regular appointments anywhere
( ) Not all the services I needed were offered in one place
( ) It wasn't available in the area
( ) Didn't think it was necessary

( ) I experienced insults or shaming from medical staff
( ) Other, please specify: ____

Page entry logic: If HIV-positive

HIV Care: Transportation and Cost

How long did you travel to get to your *most recent HIV care visit*?
( ) Less than 15 minutes
( ) 15 to 29 minutes
( ) 30 minutes to 59 minutes
( ) 1 to 2 hours
( ) More than 2 hours

How did you get to your *most recent HIV care visit*? (Tick all that apply)
[ ] By foot
[ ] By bicycle
[ ] By personal car (mine, my family's or my friend's)
[ ] By taxi
[ ] By public bus or train
[ ] Other, please specify: ____

How much did it cost to go to your *most recent HIV care visit*?
( ) Between R0 and R10
( ) Between R11 and R50
( ) Between R51 and R100
( ) Over R100

Page entry logic: If HIV-positive

HIV Care: CD4 Tests

Have you ever had a CD4 test?
( ) Yes
( ) No
( ) Don't know

Logic: If yes

In what month and year did you have your ***most recent*** CD4 test?

Month
Drop-down: Don't know, January-December

Year
Drop-down: Don't know, 2014-2000, Before 2000

What was the result of your *most recent* CD4 test?
( ) 0-49
( ) 50-99
( ) 100-199
( ) 200-349
( ) 350-499
( ) 500 or more
( ) Don't know

How many CD4 tests have you had in the past 12 months (since [question("value"), id="1778"])?
( ) 0
( ) 1
( ) 2
( ) 3
( ) 4
( ) 5
( ) More than 5

**Page entry logic:** If HIV-positive

HIV Care: Viral Load Tests

Have you ever had an HIV viral load test?
( ) Yes
( ) No
( ) Don't know

**Logic: If yes**

In what month and year did you have your ***most recent*** HIV viral load test?
Month
Drop-down: Don't know, January-December

Year
Drop-down: Don't know, 2014-2000, Before 2000

What was the result of your *most recent* HIV viral load test?
( ) Detectable
( ) Undetectable
( ) Don't know

How many HIV viral load tests have you had in the past 12 months (since [question("value"), id="1778"])?
( ) 0
( ) 1
( ) 2
( ) 3
( ) 4
( ) 5
( ) More than 5

**Page entry logic:** If HIV-positive

HIV Care: ART

Are you currently taking any antiretroviral medicines to treat your HIV?
( ) Yes
( ) No

**Logic: If no**

What are the reasons you aren't currently taking any antiretroviral medicines? (Tick all that apply)
[ ] Doctor advised to delay treatment
[ ] Drug shortage
[ ] Have not been in care long enough
[ ] CD4 count and/or viral load are good
[ ] Feel good, don't need them
[ ] Worried about side effects
[ ] Drinking or using drugs
[ ] Don't want to think about being HIV positive
[ ] No money

[ ] No insurance
[ ] Don't know where to get antiretroviral medications
[ ] Worried about remembering to take pills
[ ] Taking alternative or traditional medicines
[ ] Don't want people to know I am HIV positive
[ ] Doctor never proposed or spoke to me about antiretroviral medications
[ ] Other, please specify: ____

Main reason no ART

You selected the following reasons you are not currently taking any antiretroviral medicines. Which reason is the most important?

Arrest and Incarceration

Now we are going to ask you some questions about things you have done recently. Remember, all of your answers will be held in confidence and will not be shared with study counselors or providers.

In the past 12 months (since [question("value"), id="1778"]), have you been arrested?
( ) Yes
( ) No

Alcohol Use

One drink is equivalent to a 330ml beer, a 140ml glass of wine or a drink with one shot of spirits or liquor, and includes beer, wine, spirits or sorghum beer.

During the past 30 days, how many days did you have at least one drink containing alcohol?
Drop-down: None, 1-30

**Logic: If >0**

During the past 30 days, on the days that you drank, about how many drinks did you drink on average?
Drop-down: 1-10, more than 10

During the past 30 days, how many days did you have five or more drinks on one occasion?
Drop-down: 0-30

Drug use

In the past 6 months (since [question("value"), id="2331"]), have you used any drugs, other than those prescribed for you?

( ) Yes
( ) No

Logic: If yes

In the past 6 months (since [question("value"), id="2331"]), how often did you use the following drugs, without a prescription?

	**Didn't use**	**Once a month**	**Once a week**	**More than once a week**
Crystal meth (Tik or ice)	( )	( )	( )	( )
Crack cocaine (rocks)	( )	( )	( )	( )
Powdered cocaine	( )	( )	( )	( )
Mandrax	( )	( )	( )	( )
Ecstasy (XTC or MDMA)	( )	( )	( )	( )
Marijuana / Weed / Dagga	( )	( )	( )	( )
Heroin	( )	( )	( )	( )
CAT / khat	( )	( )	( )	( )
GHB / Liquid E	( )	( )	( )	( )
LSD or mushrooms	( )	( )	( )	( )
Poppers (amyl nitrate)	( )	( )	( )	( )
Special K (Ketamine)	( )	( )	( )	( )
Speed	( )	( )	( )	( )
Sugars	( )	( )	( )	( )
Prescription pain killers such as Codeine or Wellconal	( )	( )	( )	( )
Ritalin	( )	( )	( )	( )
Seroquel	( )	( )	( )	( )
Inhalants (Glue, others)	( )	( )	( )	( )
Other	( )	( )	( )	( )

What other drugs/injection drugs

Specify the drug or drugs you used, other than those listed on the previous screen:

____

In the past 6 months (since [question("value"), id="2331"]), did you inject any drugs, other than those prescribed for you?

( ) Yes
( ) No

**Logic: If yes**

In the past 6 months (since [question("value"), id="2331"]), did you share needles or other injection materials with anyone?

( ) Yes
( ) No

HIV Care: Individual Counseling Services

HIV prevention counseling is a service where you can talk to a counselor about your HIV-related risk behaviors, and develop a plan to maintain or improve your health behaviors and access appropriate services based on your HIV test results

Are you aware of an HIV testing service that includes HIV counseling?

( ) Yes
( ) No

**Logic: If yes**

Have you ever *received* an HIV testing service that includes counseling?

( ) Yes
( ) No

HIV Care: Couples' Counseling Services

Couples voluntary counseling and testing is a service where you and your partner can receive your test results and HIV prevention counseling together. HIV prevention counseling is a service where you can talk to a counselor about how to stay negative, cope with an HIV-positive test result, or how to prevent spreading HIV to others.

Have you ever heard of couples voluntary counseling and testing?

( ) Yes
( ) No

Logic: If yes

Have you ever *received* this service with a partner?
( ) Yes
( ) No

Do you think you would use this service if it was available to you?
( ) Yes
( ) No

PrEP Knowledge & Acceptability

If you would like to view this video in Xhosa or Afrikaans instead, click the corresponding link below.

Afrikaans Video

Xhosa Video

Logic: Show/hide trigger exists.

Before today, have you heard about taking a pill every day to prevent HIV infection?
( ) Yes
( ) No

Logic: Dynamically shown if "Before today, have you heard about taking a pill every day to prevent HIV infection?" = Yes

Are you currently taking a pill every day to prevent HIV infection?
( ) Yes
( ) No

Page entry logic: If not HIV-positive

PrEP Knowledge & Acceptability

How likely would you be to take a pill every day to prevent HIV infection if it were offered free of cost?

( ) Very likely
( ) Likely
( ) Neutral
( ) Unlikely
( ) Very unlikely

Why would you consider taking a pill every day to prevent HIV infection? (Tick all that apply)

[ ] I have had unprotected sex in the past, and may do so in the future
[ ] I have a sex partner(s) who is HIV positive
[ ] I have a sex partner(s) whose HIV status I don't know
[ ] I'm interested in extra ways to protect myself from HIV
[ ] My doctor has recommended it
[ ] My partner has recommended it
[ ] A close friend or family member recommended it
[ ] I have taken it before
[ ] Some other reason, please specify: ____

Why would you not consider taking a pill every day to prevent HIV infection? (Tick all that apply)

[ ] I am not at risk of being infected with HIV
[ ] I don't want to have to take medication every day
[ ] I don't want to have to see my doctor every 3 months
[ ] I don't want to get a HIV test every 3 months
[ ] I don't think it works well at preventing HIV infection
[ ] I don't want to have nausea or weight loss
[ ] Some other reason, please specify: ____

**Page entry logic:** If not HIV-positive

PrEP Cost

Currently some medical aid plans do not cover the cost of this HIV prevention strategy. How much would you be willing to pay each month to take a pill every day to reduce your risk of HIV infection?

( ) Nothing, I am not willing to take it even if it was free
( ) I would only take it if it was free
( ) I would only take it if it cost me less than R100 per month
( ) I would only take it if it cost me less than R250 per month

( ) I would only take it if it cost me less than R350 per month
( ) I would only take it if it cost me less than R450 per month
( ) I would take it regardless of the cost

Page entry logic: If not HIV-positive

PrEP Dosing Regimens

Now imagine you were told that instead of taking a pill every day to prevent HIV infection, there were other options for how often you would need to take pills.

To prevent HIV infection, how likely would you be to take a pill twice per week, and an extra pill within two hours after you have sex?

( ) Very likely
( ) Likely
( ) Neutral
( ) Unlikely
( ) Very unlikely

To prevent HIV infection, how likely would you be to take one pill 24-48 hours before sex, and an extra pill within two hours after you have sex?

( ) Very likely
( ) Likely
( ) Neutral
( ) Unlikely
( ) Very unlikely

PEP Knowledge & Acceptability

Please watch this video to learn more about a method of taking pills for 28 days after a high risk exposure to prevent getting HIV. The information in this video will be important for the next set of questions.

If you would like to view this video in Xhosa or Afrikaans instead, click the corresponding link below.

Afrikaans Video

Xhosa Video

Have you previously heard about taking pills used to treat HIV for 28 days after an exposure in order to prevent HIV infection?

( ) Yes
( ) No

Page entry logic: If not HIV-positive

PEP Knowledge & Acceptability

If you had a high risk exposure, such as a condom breaking with a HIV-positive partner, how likely would you be to take pills used to treat HIV for 28 days after the exposure in order to prevent HIV infection, if it were offered free of cost?

( ) Very likely
( ) Likely
( ) Neutral
( ) Unlikely
( ) Very unlikely

Why would you consider taking pills used to treat HIV for 28 days after a high risk exposure in order to prevent HIV infection? (Tick all that apply)

[ ] I have had unprotected sex in the past, and may do so in the future
[ ] I have a sex partner(s) who is living with HIV
[ ] I have a sex partner(s) whose HIV status I don't know their HIV status
[ ] I'm interested in extra ways to protect myself from HIV
[ ] My doctor has recommended it
[ ] My partner has recommended it
[ ] A close friend or family member recommended it
[ ] Some other reason, please specify: ____

Why would you not consider taking pills used to treat HIV for 28 days after a high risk exposure in order to prevent HIV infection? (Tick all that apply)

[ ] I am not at risk of being infected with HIV
[ ] I don't want to have to take medication every day
[ ] I don't want to have to have to go see my doctor
[ ] I don't think it works well at preventing HIV infection
[ ] I don't want to have nausea or weight loss
[ ] Some other reasons, please specify: ____

MSM term for stigma scales

There are different ways of referring to guys we know who have sex with men. We want to respect your preferences. For these next questions, how would you like us to refer to you as a man who has sex with other men? Please pick one:

( ) Gay

( ) Bisexual

( ) Man-loving

( ) MSM

( ) Transgender

( ) Homosexual

( ) Straight

MSM Stigma Enacted

We would like to ask you some questions about stigma related to being a [question("option title"), id="3275"] man.

In the <u>past</u> 12 months, how often have the following happen to you because you were a [question("option title"), id="3275"] man?

	Never	Once	2-3 times	4 or more times	Does not apply
Hit or beaten up?	( )	( )	( )	( )	( )
Treated rudely or unfairly?	( )	( )	( )	( )	( )
Made fun of or called names?	( )	( )	( )	( )	( )
Felt uncomfortable in a crowd of [question("option title"), id="3275"] men in your city?	( )	( )	( )	( )	( )
Lost employment or dismissed from job?	( )	( )	( )	( )	( )
Were rejected by family members?	( )	( )	( )	( )	( )
Excluded from activities traditionally reserved for men?	( )	( )	( )	( )	( )

MSM Stigma Coping

Stigma is generally defined as when a group of people are discredited by their society, often because others dislike their particular characteristic, occupation, or behavior.

In the <u>past</u> 12 months, how often did you do the following due to stigma against you as a [question("option title"), id="3275"] man?

	Never	Rarely	Sometimes	Often	Does not apply
I stayed inside to avoid facing stigma	( )	( )	( )	( )	( )

against [question("option title"), id="3275"] men.					
I had sexual relationships with girls to hide that I am a [question("option title"), id="3275"] man.	( )	( )	( )	( )	( )
I flirted with girls to hide that I am a [question("option title"), id="3275"] man.	( )	( )	( )	( )	( )
I avoided holding hands or being affectionate with a male partner in public environments.	( )	( )	( )	( )	( )
I acted more manly than usual in order to be accepted.	( )	( )	( )	( )	( )
I acted differently at work so people would not think that I am a [question("option title"), id="3275"] man.	( )	( )	( )	( )	( )
I avoided going out at night, such as going dancing.	( )	( )	( )	( )	( )

MSM Stigma Anticipated

If people are aware or become aware that you are a [question("option title"), id="3275"] man, how likely is it that they will treat you in the following ways in the <u>future</u> because you are a [question("option title"), id="3275"] man?

	Very unlikely	Unlikely	Neutral	Likely	Very Likely
An employer will look down on me	( )	( )	( )	( )	( )
Family members will have negative attitudes towards me	( )	( )	( )	( )	( )
Friends will avoid me	( )	( )	( )	( )	( )
Family members will not invite me to social gatherings	( )	( )	( )	( )	( )
My neighbors will discriminate against me	( )	( )	( )	( )	( )
People at work will assume I have many sex partners	( )	( )	( )	( )	( )
My community will treat me with less respect	( )	( )	( )	( )	( )
Someone will hit or beat me up	( )	( )	( )	( )	( )
Someone will sexually assault me	( )	( )	( )	( )	( )
I will be called hurtful words when I go outside my home	( )	( )	( )	( )	( )

MSM Stigma Internalized

How do you feel about being a [question("option title"), id="3275"] man? Please rate how much you agree with the following statements.

	Strongly Disagree	Disagree	Neutral	Agree	Strongly Agree
If I could change being a [question("option title"), id="3275"] man to be a man who has sex only with women, I would do it.	( )	( )	( )	( )	( )
If people call me names, I am good at ignoring it.	( )	( )	( )	( )	( )
I feel ashamed of being a [question("option title"), id="3275"] man.	( )	( )	( )	( )	( )
Social involvement with other [question("option title"), id="3275"] men makes me feel uncomfortable.	( )	( )	( )	( )	( )
I feel I am not as good as others because I am a [question("option title"), id="3275"] man.	( )	( )	( )	( )	( )
I think less of myself when I am in public with a person who is obviously a [question("option title"), id="3275"] man.	( )	( )	( )	( )	( )
I think being a [question("option title"), id="3275"] man is against the will of God.	( )	( )	( )	( )	( )
I perceive myself as physically or emotionally weak because I am a [question("option title"), id="3275"] man.	( )	( )	( )	( )	( )

MSM Stigma Healthcare Provider

In the past 12 months, how often have the following happened to you because someone knew or assumed you were a [question("option title"), id="3275"] man?

	Never	Once	2-3 times	4 or more times	Does not apply
I felt afraid to go to health care services at public clinics.	( )	( )	( )	( )	( )
I avoided going to health care services at public clinics.	( )	( )	( )	( )	( )
I was denied health care services at public clinics.	( )	( )	( )	( )	( )

I was not treated well when receiving healthcare services at public clinics.	( )	( )	( )	( )	( )

In the past 12 months, how often have the following happened to you because someone knew or assumed you were a [question("option title"), id="3275"] man?

	Never	Once	2-3 times	4 or more times	Does not apply
I felt afraid to go to health care services at private clinics.	( )	( )	( )	( )	( )
I avoided going to health care services at private clinics.	( )	( )	( )	( )	( )
I was denied health care services at private clinics.	( )	( )	( )	( )	( )
I was not treated well when receiving healthcare services at private clinics.	( )	( )	( )	( )	( )

Page entry logic: This page will show when: (Question "What was the result of your most recent HIV test?" #50 contains any ("Positive") OR Question "Have you ever gotten a positive test result for HIV?" #52 contains any ("Yes"))

HIV Stigma Enacted

We would also like to ask you some questions about stigma related to living with HIV.

In the past 12 months, how often did the following happen to you because you are HIV-positive?

	Never	Once	2-3 times	4 or more times	Does not apply
I lost friends by telling them I am HIV-positive.	( )	( )	( )	( )	( )
Family members looked down on me.	( )	( )	( )	( )	( )
People treated me with less respect.	( )	( )	( )	( )	( )
People didn’t want me around their children once they knew that I am HIV-positive.	( )	( )	( )	( )	( )
People cut down visiting me once they knew that I am HIV-positive.	( )	( )	( )	( )	( )

Page entry logic: This page will show when: (Question "What was the result of your most recent HIV test?" #50 contains any ("Positive") OR Question "Have you ever gotten a positive test result for HIV?" #52 contains any ("Yes"))

HIV Stigma Anticipated

If people are aware or become aware that you are HIV-positive, how likely is it that they will treat you in the following ways in the future because you are HIV-positive?

	Very unlikely	Unlikely	Neutral	Likely	Very likely
People will discriminate against me	( )	( )	( )	( )	( )
People will judge me	( )	( )	( )	( )	( )
People will think I am disgusting	( )	( )	( )	( )	( )
People will reject me	( )	( )	( )	( )	( )
People will be uncomfortable around me	( )	( )	( )	( )	( )
People will look for flaws in my character	( )	( )	( )	( )	( )

Page entry logic: This page will show when: (Question "What was the result of your most recent HIV test?" #50 contains any ("Positive") OR Question "Have you ever gotten a positive test result for HIV?" #52 contains any ("Yes"))

HIV Stigma Internalized

Please rate how much you agree with the following statements.

	Strongly Disagree	Disagree	Neutral	Agree	Strongly Agree
Being HIV-positive makes me feel that I'm a bad person.	( )	( )	( )	( )	( )
I feel I'm not as good as others because I am HIV-positive.	( )	( )	( )	( )	( )
Being HIV-positive makes me feel unclean.	( )	( )	( )	( )	( )
I never feel ashamed of being HIV-positive.	( )	( )	( )	( )	( )

Page entry logic: This page will show when: (Question "What was the result of your most recent HIV test?" #50 contains any ("Positive") OR Question "Have you ever gotten a positive test result for HIV?" #52 contains any ("Yes"))

HIV Stigma Health Care Provider

In the past 12 months, how often have the following happened to you because someone knew or assumed you are HIV-positive?

	Never	Once	2-3 times	4 or more times	Does not apply
I was discharged from the hospital or clinic while still needing care.	( )	( )	( )	( )	( )

I was shuttled around instead of being helped by a nurse.	( )	( )	( )	( )	( )
In the hospital or clinic, my pain was ignored.	( )	( )	( )	( )	( )
I was refused treatment because I was told I was going to die anyway.	( )	( )	( )	( )	( )
At the hospital or clinic, I was left in a soiled bed.	( )	( )	( )	( )	( )
I was denied healthcare.	( )	( )	( )	( )	( )

Page entry logic: This page will show when: (Question "What was the result of your most recent HIV test?" #50 not in list ("Positive") AND Question "Have you ever gotten a positive test result for HIV?" #52 not in list ("Yes"))

HIV Stigma Scale for HIV-negative men

Please rate how much you agree with the following statements?

	**Strongly disagree**	**Disagree**	**Neutral**	**Agree**	**Strongly agree**
[question("option title"), id="3275"] men with HIV should be treated with respect.	( )	( )	( )	( )	( )
I am uncomfortable around [question("option title"), id="3275"] men with HIV.	( )	( )	( )	( )	( )
I would not want a person with HIV to be around children in my family.	( )	( )	( )	( )	( )
I visited a friend or family member less once I knew they had HIV.	( )	( )	( )	( )	( )
I treat [question("option title"), id="3275"] men with HIV the same as I treat other [question("option title"), id="3275"] men.	( )	( )	( )	( )	( )

Condoms

Now we would like to ask you about access to condoms and lubricant and how you feel about using them.

Have you gotten condoms in the past 3 months (since [question("value"), id="2939"])?

( ) Yes

( ) No

Logic: If yes

Where have you gotten condoms in the past 3 months (since [question("value"), id="2939"])? (Tick all that apply)

[ ] Pharmacy
[ ] Health clinic
[ ] LGBT or HIV community-based organization
[ ] My partner has gotten the condoms we used
[ ] Other, please specify: ____

Did you pay for any of the condoms you got?

( ) Yes
( ) No

Lube

Have you gotten water- or silicone-based lube in the past 3 months (since [question("value"), id="2939"])?

( ) Yes
( ) No

Logic: If no

Do you know where you can get water- or silicone-based lube if you needed it?

( ) Yes
( ) No

Logic: If yes

Where have you gotten lubrication products in the past 3 months (since [question("value"), id="2939"])? (Tick all that apply)

[ ] Pharmacy
[ ] Health clinic
[ ] LGBT or HIV community-based organization
[ ] My partner has gotten the lube we used
[ ] Other, please specify:: ____

Did you pay for any of the lube you got?

( ) Yes
( ) No

Condom Errors

In the past 6 months (since the beginning of [question("value"), id="2331"]), have you had anal sex as the insertive partner (top) and used a condom?

( ) Yes
( ) No

Logic: If yes

In the last 6 months (since the beginning of [question("value"), id="2331"]), when you used a condom as the insertive partner (top), did you:

	Yes	No	Don't know
Completely unroll the condom before putting it on?	( )	( )	( )
Squeeze air from the tip of the condom before putting it on?	( )	( )	( )
Leave space at the tip of the condom?	( )	( )	( )
Start having sex, then put the condom on later?	( )	( )	( )
Hold the base of the condom during withdrawal?	( )	( )	( )
Experience a condom breaking?	( )	( )	( )
Experience a condom slipping off your or your partner's penis?	( )	( )	( )
Put the condom on inside out, then flip it over to use?	( )	( )	( )
Re-use a condom?	( )	( )	( )

Condom Attitudes

Please rate how much you agree or disagree with the following statements about condoms.

	Strongly disagree	Moderately disagree	Neutral	Moderately agree	Strongly agree
Condoms are effective at preventing pregnancy	( )	( )	( )	( )	( )
Condoms are	( )	( )	( )	( )	( )

effective at preventing sexually transmitted infections					
Condoms are effective at preventing HIV	( )	( )	( )	( )	( )
Condoms are comfortable	( )	( )	( )	( )	( )
Condoms are convenient to use	( )	( )	( )	( )	( )
Condoms decrease sexual pleasure	( )	( )	( )	( )	( )
Condoms are easy to obtain	( )	( )	( )	( )	( )

Condom Attitudes page 2

Please rate how much you agree or disagree with the following statements about condoms.

	**Strongly disagree**	**Moderately disagree**	**Neutral**	**Moderately agree**	**Strongly agree**
Condoms are something my friends hate	( )	( )	( )	( )	( )
Condoms are something my sexual partners hate	( )	( )	( )	( )	( )
Condoms are exciting	( )	( )	( )	( )	( )
Condoms are embarrassing	( )	( )	( )	( )	( )
Condoms are difficult to discuss with a partner	( )	( )	( )	( )	( )
Condoms are difficult to use	( )	( )	( )	( )	( )
Condoms are messy	( )	( )	( )	( )	( )

Partner genders and numbers: Past 12 Months

During the past 12 months (since [question("value"), id="1778"]), did you have vaginal or anal sex with a person who was: (tick all that apply)*

[ ] Male
[ ] Female
[ ] Transgender

**Page entry logic:** If any male partners

During the past 12 months (since [question("value"), id="1778"]), with how many men did you have anal sex?*

____

**Page entry logic:** If 1 male partner

How would you describe your male anal sex partner?

( ) Main partner (someone that you feel committed to above all others – this is someone you might call your boyfriend, significant other, life partner, or husband)
( ) Casual partner (someone that you do not feel committed to above all others)

In the past 12 months, did you have unprotected anal sex with your male anal sex partner? (This means that you and your partner had anal sex at least once without using a condom or not using it the whole time)

( ) Yes
( ) No
( ) Don't know

Did you have anal sex with your male partner in exchange for money, drugs, food, or something else of value?

( ) Yes
( ) No
( ) Don't know

**Logic: If yes**

Who gave money, drugs, food or something else to who in exchange for anal sex?

( ) I gave money, drugs, food or something else of value to my partner

( ) My partner gave money, drugs, food, or something else of value to me

Page entry logic: If >1 male partner

Of your [%%2364: &n %%] male partners you had anal sex with in the past 12 months, how many were:

________**Main partners?** (someone that you feel committed to above all others – this is someone you might call your boyfriend, significant other, life partner, or husband)
________**Casual partners?** (someone that you do not feel committed to above all others)

Of your [%%2364: &n %%] male partners you had anal sex with in the past 12 months, how many did you have unprotected anal sex with? (This means that you and your partner had anal sex at least once without using a condom or not using it the whole time)

____

Did you have anal sex with any of your [%%2364: &n %%] male partners in exchange for money, drugs, food, or something else of value?

( ) Yes
( ) No

Logic: If yes

Of your male partners that you had anal sex with in exchange for money, drugs, food, or something else of value, how many:

________Did you give money, drugs, food or something else of value to?
________Gave you money, drugs, food, or something else of value?

Page entry logic: If any female partners

During the past 12 months (since [question("value"), id="1778"]), with how many women did you have vaginal or anal sex?*

____

Page entry logic: If 1 female partner

How would you describe your female vaginal or anal sex partner?

( ) Main partner (someone that you feel committed to above all others – this is someone you might call your girlfriend, significant other, life partner, or wife)
( ) Casual partner (someone that you do not feel committed to above all others)

In the past 12 months, did you have unprotected vaginal or anal sex with your female sex partner? (This means that you and your partner had vaginal or anal sex at least once without using a condom or not using it the whole time)
( ) Yes
( ) No
( ) Don't know

Did you have vaginal or anal sex with your female sex partner in exchange for money, drugs, food, or something else of value?
( ) Yes
( ) No
( ) Don't know

**Page entry logic:** If >1 female partner

Of your [%%2458: &n %%] female partners you had vaginal or anal sex with in the past 12 months, how many were:
________**Main partners?** (someone that you feel committed to above all others – this is someone you might call your girlfriend, significant other, life partner, or wife)
________**Casual partners?** (someone that you do not feel committed to above all others)

Of your [%%2458: &n %%] female partners you had vaginal or anal sex with in the past 12 months, how many did you have unprotected vaginal or anal sex with? (This means that you and your partner had vaginal or anal sex at least once without using a condom or not using it the whole time)

____

Of your [%%2458: &n %%] female partners you had sex with in the past 12 months, how many did you have vaginal or anal sex with in exchange for money, drugs, food, or something else of value?

____

**Page entry logic:** If transgender partners

During the past 12 months (since [question("value"), id="1778"]), with how many transgender individuals did you have vaginal or anal sex?*

____

Page entry logic: If 1 transgender partner

How would you describe your transgender vaginal or anal sex partner?
( ) Main partner (someone that you feel committed to above all others – this is someone you might call your boyfriend, significant other, life partner, or husband)
( ) Casual partner (someone that you do not feel committed to above all others)

In the past 12 months, did you have unprotected vaginal or anal sex with your transgender sex partner? (This means that you and your partner had vaginal or anal sex at least once without using a condom or not using it the whole time)
( ) Yes
( ) No
( ) Don't know

Did you have vaginal or anal sex with your transgender sex partner in exchange for money, drugs, food, or something else of value?
( ) Yes
( ) No
( ) Don't know

Page entry logic: If >1 transgender partner

Of your [%%2463: &n %%] transgender partners you had vaginal or anal sex with in the past 12 months, how many were:
________**Main partners?** (someone that you feel committed to above all others – this is someone you might call your significant other or life partner)
________**Casual partners?** (someone that you do not feel committed to above all others)

Of your [%%2463: &n %%] transgender partners you had vaginal or anal sex with in the past 12 months, how many did you have unprotected vaginal or anal sex with? (This means that you and your partner had vaginal or anal sex at least once without using a condom or not using it the whole time)

____

Of your [%%2463: &n %%] transgender partners you had sex with in the past 12 months, how many did you have vaginal or anal sex with in exchange for money, drugs, food, or something else of value?

____

Sex partners: Past 3 Months

Now we would like to ask about your sex partners in the **past 3 months (since [question("value"), id="2939"])**.

Logic: If 1 male sex partner in last 12 months

Did you have anal sex with your male partner in the past 3 months?

( ) Yes
( ) No

Logic: If >1 male sex partner in last 12 months

Of your [%%2364: &n %%] male partners in the past 12 months, how many did you have anal sex with in the past 3 months?

____

Logic: Logic: If 1 female sex partner in last 12 months

Did you have vaginal or anal sex with your female partner in the past 3 months?

( ) Yes
( ) No

Logic: Logic: If >1 female sex partner in last 12 months

Of your [%%2458: &n %%] female partners in the past 12 months, how many did you have vaginal or anal sex with in the past 3 months?

____

Logic: Logic: If 1 transgender sex partner in last 12 months

Did you have vaginal or anal sex with your transgender partner in the past 3 months?

( ) Yes
( ) No

Logic: If >1 transgender sex partner in last 12 months

Of your [%%2463: &n %%] transgender partners in the past 12 months, how many did you have vaginal or anal sex with in the past 3 months?

____

Introduction: partner list

This next section will be about some of your recent sex partners, regardless of gender.
In order to do that, we will ask you to provide nicknames for your recent sex partners. We will then use these nicknames to customize the questions so that they are specific to your partners.

It is important that you choose a nickname that will best help you remember the person. The names you provide are meant to help you only and should not reveal your partner's full identity. We do not want to know who your partners actually are.

Some examples of nicknames that you might choose are: a partner's first name, a nickname you call the partner by, the place where you both met or an online login name.

Partner list

Please give a nickname for each of your most recent sex partners over the last 3 months
(since [question("value"), id="2939"]).

Male, female, and transgender sex partners may be in this list:

- For **male** partners, we mean people you had **anal sex** with.

- For **female** partners, we mean people you had **vaginal or anal sex** with.
- For transgender partners, we mean male to female transgender partners you had **vaginal or anal sex** with.

Space for 3 partners is provided, but you only need to fill in as many spaces as you need or can remember.
If you had more than 3 sex partners in the previous 3 months, please provide nicknames for the most recent 3.
*****
Partner 1 (most recent): ____
Partner 2: ____
Partner 3: ____

NEXT SECTION ASKED FOR EACH PARTNER LISTED:

Partner #1: Demographics

Please tell us a little about **[question("option value"), id="403", option="10392"]**.

Is [question("option value"), id="403", option="10392"] male, female, or transgender?*
( ) Male
( ) Female
( ) Transgender

What is the current age of [question("option value"), id="403", option="10392"]?
Drop-down: Don't know, 13-80, older than 80

**Logic: If don't know**

Which of the following statements about [question("option value"), id="403", option="10392"] is most true?
( ) He/she is more than 10 years younger than I am
( ) He or she is 2 to 10 years younger than I am
( ) He or she is within a year of my age
( ) He or she is 2 to 10 years older than I am
( ) He or she is more than 10 years older than I am
( ) Don't know

What race is [question("option value"), id="403", option="10392"]?
( ) Black

( ) Coloured
( ) Indian or Asian
( ) White
( ) Other: ____

Logic: If male or transgender

Does [question("option value"), id="403", option="10392"] tell other people that [question("option value"), id="403", option="10392"] has sex with men?

( ) Yes
( ) No
( ) I don't know

How would [question("option value"), id="403", option="10392"] think of themself?

( ) Heterosexual or Straight
( ) Homosexual or Gay
( ) Bisexual
( ) Other, please specify:: ____
( ) Don't know

Partner #1 Partnership Description

Please tell us a little about you and [question("option value"), id="403", option="10392"].

Did you have sex with [question("option value"), id="403", option="10392"] once, or more than once during the past 3 months?*

( ) Once
( ) More than once

Is/was [question("option value"), id="403", option="10392"] someone that you feel or felt committed to above all others (someone you might call your boyfriend, significant other, life partner, husband, or wife)?

( ) Yes
( ) No
( ) Don't know

Logic: If more than once

In the past 3 months, how often did you use condoms with [question("option value"), id="403", option="10392"] ?

( ) Always
( ) Most of the time

( ) Sometimes
( ) Rarely
( ) Never

Partner #1: Geography

Where did you first meet [question("option value"), id="403", option="10392"]?

( ) Through friends
( ) School or work
( ) At a party
( ) Bar or club or shebeen
( ) Bath house or sauna
( ) Gym
( ) Public place (e.g. park or beach)
( ) Online

**Logic: If online**

Through which online service did you first meet [question("option value"), id="403", option="10392"]?

( ) MXit
( ) Facebook
( ) 2go
( ) Friendo
( ) WhatsApp
( ) ManHunt
( ) GayDar
( ) Hookups
( ) Mamba Online
( ) Gum Tree
( ) Other, please specify:: ____

Did you and [question("option value"), id="403", option="10392"] live together at any point during the past 3 months (since [question("value"), id="2939"])?

( ) Yes
( ) No

Partner #1: Disclosure of status

Did you and [question("option value"), id="403", option="10392"] share both of your HIV statuses before you first had sex?

( ) Yes
( ) No
( ) Don't know

**Logic: If yes**

What was [question("option value"), id="403", option="10392"]'s status at that time?

( ) HIV-negative
( ) HIV-positive
( ) Don't remember

**Logic: If no**

What do you believe [question("option value"), id="403", option="10392"]'s status was at that time?

( ) HIV-negative
( ) HIV-positive
( ) Don't know

**Page entry logic:** If sex with partner once

Partner #1: Date of sex - one time partner

You indicated that you’ve had sex with [question("option value"), id="403", option="10392"] just once.

When did you have sex with [question("option value"), id="403", option="10392"]?

You may enter just the month and year, but if you know the exact date, please enter that instead.

If you are unsure of when you last had sex, try to select a time that you think is close.

( ) [question("value"), id="421"]
( ) [question("value"), id="428"]
( ) [question("value"), id="429"]

( ) [question("value"), id="2329"]

OR
Exact Date: ____

**Page entry logic: If sex with partner > once**

Partner #1: Date of first sex

When did you ***first*** have sex with [question("option value"), id="403", option="10392"]?

> You may enter just the month and year, but if you know the exact date, please enter that instead.
>
> It's OK if the first time you had sex with [question("option value"), id="403", option="10392"] was longer than 3 months ago. We still would like to know when you first had sex.

Month
Drop-down: Don't know the year, January-December

Year
Drop-down: Don't know the month, 2014-2000, Before 2000

OR
Exact date:____

Partner 1 - ask for month/date ranges

**Logic: If don’t know the month**

It’s OK if you can’t remember the exact month.

> **Think back to the time when you first had sex with [question("option value"), id="403", option="10392"].**

Perhaps you had sex around a special time of the year such as your birthday, or a public holiday like Freedom Day or Boxing Day.. Maybe you can remember that it was warm outside or it was after a trip you took.

Based on what you can recall, try to select what time during the year you first had sex with [question("option value"), id="403", option="10392"]:

( ) January - March
( ) April - June
( ) July - September
( ) October - December
( ) (don't know when during the year)

**Logic: If don't know the year**

It's OK if you can't remember the exact year.

Can you remember about how many years ago you first had sex with [question("option value"), id="403", option="10392"]?

( ) Less than 1 year ago
( ) 1 – 2 years ago
( ) 3 – 5 years ago
( ) 6 – 10 years ago
( ) more than 10 years ago

Now we are going to ask you about the **last time** you had sex with **[question("option value"), id="403", option="10392"]**.

**Page entry logic:** If sex with partner > once

Partner #1: Date of last sex

When did you *last* have sex with [question("option value"), id="403", option="10392"]?

You may enter just the month and year, but if you know the exact date, please enter that instead.

If you are unsure of when you last had sex, try to select a time that you think is close.

(Last 4 months)
( ) [question("value"), id="421"]
( ) [question("value"), id="428"]
( ) [question("value"), id="429"]
( ) [question("value"), id="2329"]

OR
Exact date:____

Partner #1: Ongoing Relationship?

Do you think you will have sex with [question("option value"), id="403", option="10392"] again?
( ) Yes
( ) No
( ) Not sure

**Page entry logic:** If sex with partner> once

Partner #1: Sex Frequency

Now we'd like to ask you a few questions about your relationship with [question("option value"), id="403", option="10392"] in the last 3 months (since [question("value"), id="2939"]).

In the past 3 months (since [question("value"), id="2939"]), how many times have you had anal sex with [question("option value"), id="403", option="10392"]?
( ) 0
( ) 1
( ) 2
( ) 3
( ) 4
( ) 5
( ) 6
( ) 7
( ) 8
( ) 9
( ) 10
( ) more than 10

Logic: If more than 10

About how often did you have anal sex with [question("option value"), id="403", option="10392"] in the past 3 months (since [question("value"), id="2939"])?

( ) About once a month
( ) 2 or 3 times a month
( ) About once a week
( ) 2 or 3 times a week
( ) More than 3 times a week

Have you had unprotected anal sex with [question("option value"), id="403", option="10392"] in the past 3 months? (This means that you and [question("option value"), id="403", option="10392"] had anal sex at least once without using a condom or not using it the whole time)

( ) Yes
( ) No
( ) Don't know

Page entry logic: If partner is female/transgender and had sex >once

Partner #1: Female and Transgender

In the past 3 months (since [question("value"), id="2939"]), how many times have you had vaginal sex with [question("option value"), id="403", option="10392"]?

( ) 0
( ) 1
( ) 2
( ) 3
( ) 4
( ) 5
( ) 6
( ) 7
( ) 8
( ) 9
( ) 10
( ) more than 10

Logic: If more than 10

About how often did you have vaginal sex with [question("option value"), id="403", option="10392"] in the past 3 months (since [question("value"), id="2939"])?

( ) About once a month
( ) 2 or 3 times a month
( ) About once a week
( ) 2 or 3 times a week
( ) More than 3 times a week

Have you had unprotected vaginal sex with [question("option value"), id="403", option="10392"] in the past 3 months? (This means that you and [question("option value"), id="403", option="10392"] had vaginal sex at least once without using a condom or not using it the whole time)

( ) Yes
( ) No
( ) Don't know

Page entry logic: If sex with partner > once

Partner #1: Sexual activity outside relationship

In the past 3 months (since [question("value"), id="2939"]), during the time you were sexually involved with [question("option value"), id="403", option="10392"], with how many other people did you have sex?

( ) 0
( ) 1
( ) 2
( ) 3
( ) 4
( ) 5
( ) 6
( ) 7
( ) 8
( ) 9
( ) 10
( ) More than 10

In the past 3 months (since [question("value"), id="2939"]), during the time you were sexually involved with [question("option value"), id="403", option="10392"] , did [question("option value"), id="403", option="10392"] have sex with anyone else?

( ) Definitely did not
( ) Probably did not
( ) Probably did

( ) Definitely did
( ) Don't know

Page entry logic: If sex with partner > once

Partner #1: Last sex - intro

Now we'd like to ask you about the last time you had sex with **[question("option value"), id="403", option="10392"]**.

Some questions will be very similar to ones we asked about your sexual relationship with **[question("option value"), id="403", option="10392"]** over the last 3 months, but it is important for our study to ask these questions again.

We greatly appreciate your responses to these questions.

(Throughout section, if had sex with partner only once, "the last time" is replaced with "when")

Page entry logic: If male or transgender partner

The last time you had sex with [question("option value"), id="403", option="10392"] did you have receptive anal sex? (This means that you were the bottom)

( ) Yes
( ) No
( ) Don't know

Logic: If yes

Was a condom used the last time you had receptive anal sex (when you were the bottom)?

( ) [question("option value"), id="403", option="10392"] did not use a condom
( ) [question("option value"), id="403", option="10392"] used a condom part of time
( ) [question("option value"), id="403", option="10392"] used a condom the whole time
( ) [question("option value"), id="403", option="10392"] used a condom, but it broke
( ) Don't know

Logic: If no condoms

What are the reasons why a condom was not used the last time you had receptive anal sex with [question("option value"), id="403", option="10392"]? (Tick all that apply)

[ ] I believe condoms decrease pleasure
[ ] I believe condoms negatively impact sex (harder to get erection, harder to reach orgasm)
[ ] I was in a trusted relationship, and condoms are not necessary
[ ] I was embarrassed to buy them
[ ] I was drunk or high
[ ] [question("option value"), id="403", option="10392"] preferred not to use condoms
[ ] A condom was not conveniently available
[ ] Other reason, please specify: ____

**Logic: If condoms used**

Why was a condom used the last time you had receptive anal sex with [question("option value"), id="403", option="10392"]? (Tick all that apply)

[ ] I value my life, and it is worth protecting
[ ] It was a casual partnership (hook-up)
[ ] I follow the rule: No condom, no sex.
[ ] I suspected or knew [question("option value"), id="403", option="10392"] has HIV
[ ] I was unaware of [question("option value"), id="403", option="10392"]'s HIV status
[ ] Condoms lessen the pain of anal sex
[ ] [question("option value"), id="403", option="10392"] preferred to use condoms
[ ] We (me or [question("option value"), id="403", option="10392"]) had a readily available supply of condoms
[ ] Other reason, please specify: ____

The last time you had receptive anal sex with [question("option value"), id="403", option="10392"], which of the following brand or type of condom did you use?

( ) Choice
( ) Durex
( ) Lovers Plus
( ) Play Nice
( ) Assegai
( ) Female condoms
( ) Other, please specify: ____

What type of lube, if any, did you use the last time you had receptive anal sex with [question("option value"), id="403", option="10392"]?

( ) We did not use any lube
( ) Water-based lube (like KY, Assegaai or JO)
( ) Silicone-based lube (like Astroglide)
( ) Oil-based lube (like Vaseline, Dawn cream, massage oil, butter or cooking oil)
( ) Spit or Saliva

( ) We used lube, but I don't know what type
( ) Other, please specify: ____

**Page entry logic:** If male or transgender partner

The last time you had sex with [question("option value"), id="403", option="10392"] , did you have insertive anal sex? (This means that you were the top)
( ) Yes
( ) No
( ) Don't know

**Logic: If yes**

Was a condom used the last time you had insertive anal sex with [question("option value"), id="403", option="10392"] (when you were the top)?
( ) I did not use a condom
( ) I used a condom part of time
( ) I used a condom the whole time
( ) I used a condom, but it broke
( ) Don't know

**Logic: If no condoms**

What are the reasons why a condom was not used the last time you had insertive anal sex with [question("option value"), id="403", option="10392"]? (Tick all that apply)
[ ] I believe condoms decrease pleasure
[ ] I believe condoms negatively impact sex (harder to get erection, harder to reach orgasm)
[ ] I was in a trusted relationship, and condoms are not necessary
[ ] I was embarrassed to buy them
[ ] I was drunk or high
[ ] [question("option value"), id="403", option="10392"] preferred not to use condoms
[ ] A condom was not conveniently available
[ ] Other reason, please: ____

**Logic: If condoms used**

Why was a condom used the last time you had insertive anal sex with [question("option value"), id="403", option="10392"]? (Tick all that apply)
[ ] I value my life, and it is worth protecting
[ ] It was a casual partnership (hook-up)

[ ] I follow the rule: No condom, no sex.
[ ] I suspected or knew [question("option value"), id="403", option="10392"] has HIV
[ ] I was unaware of [question("option value"), id="403", option="10392"]'s HIV status
[ ] Condoms lessen the pain of anal sex
[ ] [question("option value"), id="403", option="10392"] preferred to use condoms
[ ] We (me or [question("option value"), id="403", option="10392"]) had a readily available supply of condoms
[ ] Other reason, please specify: ____

The last time you had insertive anal sex with [question("option value"), id="403", option="10392"], which of the following brand or type of condom did you use?
( ) Choice
( ) Durex
( ) Lovers Plus
( ) Play Nice
( ) Assegai
( ) Female condoms
( ) Other, please specify: ____

What type of lube, if any, did you use the last time you had insertive anal sex with [question("option value"), id="403", option="10392"]?
( ) We did not use any lube
( ) Water-based lube (like KY, Assegaai or JO)
( ) Silicone-based lube (like Astroglide)
( ) Oil-based lube (like Vaseline, Dawn cream, massage oil, butter or cooking oil)
( ) Spit or Saliva
( ) We used lube, but I don’t know what type
( ) Other, please specify: ____

**Page entry logic:** If female or transgender partner

The last time you had sex with [question("option value"), id="403", option="10392"] did you have vaginal sex?
( ) Yes
( ) No
( ) Don't know

**Logic: If yes**

Was a condom used the last time you had vaginal sex?
( ) I did not use a condom
( ) I used a condom part of time
( ) I used a condom the whole time
( ) I used a condom, but it broke
( ) Don't know

Logic: If no condom

What are the reasons why a condom was not used the last time you had vaginal sex with [question("option value"), id="403", option="10392"]? (Tick all that apply)
[ ] I believe condoms decrease pleasure
[ ] I believe condoms negatively impact sex (harder to get erection, harder to reach orgasm)
[ ] I was in a trusted relationship, and condoms are not necessary
[ ] I was embarrassed to buy them
[ ] I was drunk or high
[ ] [question("option value"), id="403", option="10392"] preferred not to use condoms
[ ] A condom was not conveniently available
[ ] Other reason, please specify: ____

Logic: If condom used

Why was a condom used the last time you had vaginal sex with [question("option value"), id="403", option="10392"]? (Tick all that apply)
[ ] I value my life, and it is worth protecting
[ ] It was a casual partnership (hook-up)
[ ] I follow the rule: No condom, no sex.
[ ] I suspected or knew [question("option value"), id="403", option="10392"] has HIV
[ ] I was unaware of [question("option value"), id="403", option="10392"]'s HIV status
[ ] [question("option value"), id="403", option="10392"] preferred to use condoms
[ ] We (me or [question("option value"), id="403", option="10392"]) had a readily available supply of condoms
[ ] Other reason, please specify: ____

The last time you had vaginal sex with [question("option value"), id="403", option="10392"], which of the following brand or type of condom did you use?
( ) Choice
( ) Durex
( ) Lovers Plus
( ) Play Nice
( ) Assegai
( ) Female condoms

( ) Other, please specify: ____

What type of lube, if any, did you use the last time you had vaginal sex with [question("option value"), id="403", option="10392"]?

( ) We did not use any lube
( ) Water-based lube (like KY, Assegaai or JO)
( ) Silicone-based lube (like Astroglide)
( ) Oil-based lube (like Vaseline, Dawn cream, massage oil, butter or cooking oil)
( ) Spit or Saliva
( ) We used lube, but I don't know what type
( ) Other, please specify: ____

**Page entry logic:** If female partner

The last time you had sex with [question("option value"), id="403", option="10392"] did you have anal sex?

( ) Yes
( ) No
( ) Don't know

**Logic: If yes**

Was a condom used the last time you had anal sex with [question("option value"), id="403", option="10392"] ?

( ) I did not use a condom
( ) I used a condom part of time
( ) I used a condom the whole time
( ) I used a condom, but it broke
( ) Don't know

**Logic: If no condom**

What are the reasons why a condom was not used the last time you had anal sex with [question("option value"), id="403", option="10392"]? (Tick all that apply)

[ ] I believe condoms decrease pleasure
[ ] I believe condoms negatively impact sex (harder to get erection, harder to reach orgasm)
[ ] I was in a trusted relationship, and condoms are not necessary
[ ] I was embarrassed to buy them
[ ] I was drunk or high
[ ] [question("option value"), id="403", option="10392"] preferred not to use condoms

[ ] A condom was not conveniently available
[ ] Other reason, please specify: ____

Logic: If condom used

Why was a condom used the last time you had anal sex with [question("option value"), id="403", option="10392"]? (Tick all that apply)

[ ] I value my life, and it is worth protecting
[ ] It was a casual partnership (hook-up)
[ ] I follow the rule: No condom, no sex.
[ ] I suspected or knew [question("option value"), id="403", option="10392"] has HIV
[ ] I was unaware of [question("option value"), id="403", option="10392"]'s HIV status
[ ] Condoms lessen the pain of anal sex
[ ] [question("option value"), id="403", option="10392"] preferred to use condoms
[ ] We (me or [question("option value"), id="403", option="10392"]) had a readily available supply of condoms
[ ] Other reason, please specify: ____

The last time you had anal sex with [question("option value"), id="403", option="10392"], which of the following brand or type of condom did you use?

( ) Choice
( ) Durex
( ) Lovers Plus
( ) Play Nice
( ) Assegai
( ) Female condoms
( ) Other, please specify: ____

What type of lube, if any, did you use the last time you had anal sex with [question("option value"), id="403", option="10392"]?

( ) We did not use any lube
( ) Water-based lube (like KY, Assegaai or JO)
( ) Silicone-based lube (like Astroglide)
( ) Oil-based lube (like Vaseline, Dawn cream, massage oil, butter or cooking oil)
( ) Spit or Saliva
( ) We used lube, but I don’t know what type
( ) Other, please specify: ____

Page entry logic: All partners

Partner #1: Alcohol / Drugs

The last time you had sex with [question("option value"), id="403", option="10392"] were you tipsy or drunk on alcohol?

( ) Yes
( ) No
( ) Don't know

The last time you had sex with [question("option value"), id="403", option="10392"] were you high on drugs?

( ) Yes
( ) No
( ) Don't know

Partner transition: #1 to #2

Thank you for telling us about [question("option value"), id="403", option="10392"].

<u>Next, we'd like to ask you about [question("option value"), id="403", option="10393"].</u>

Click **Next** to continue.

Repeat Section for Partners 2 and 3

Concurrency tables

For each sex partner, tick a box for each month during which you had sex with that partner.

Logic: If 3 partners

	Month 0	Month 1	Month 2	Month 3	Month 4	Month 5
Partner 1	[ ]	[ ]	[ ]	[ ]	[ ]	[ ]
Partner 2	[ ]	[ ]	[ ]	[ ]	[ ]	[ ]
Partner 3	[ ]	[ ]	[ ]	[ ]	[ ]	[ ]

Logic: If 2 partners

	Month 0	Month 1	Month 2	Month 3	Month 4	Month 5
Partner 1	[ ]	[ ]	[ ]	[ ]	[ ]	[ ]
Partner 2	[ ]	[ ]	[ ]	[ ]	[ ]	[ ]

Logic: If 1 partner

	Month 0	Month 1	Month 2	Month 3	Month 4	Month 5
Partner 1	[ ]	[ ]	[ ]	[ ]	[ ]	[ ]

Page entry logic: This page will show when: name_count = "2"

Concurrency clarification questions

Thank you for telling us the months in which you had sex with your partners.

This page will ask some questions to help us better understand what you just told us.

Logic: Ask this for each ambiguous partnership in each month

You indicated that you had sex with both [question("option value"), id="403", option="10392"] and [question("option value"), id="403", option="10393"] in the month of [question("value"), id="2331"].

Which of these statements about [question("value"), id="2331"] is most correct?
( ) I last had sex with [question("option value"), id="403", option="10392"] before I had sex with [question("option value"), id="403", option="10393"].
( ) I last had sex with [question("option value"), id="403", option="10393"] before I had sex with [question("option value"), id="403", option="10392"].
( ) I was having sex with [question("option value"), id="403", option="10392"] and [question("option value"), id="403", option="10393"] during the same time period.
( ) Don't know

Relationships among partners

Logic: If 2 partners

Did [question("option value"), id="403", option="10392"] and [question("option value"), id="403", option="10393"] have sex with each other in the past 3 months, or do you think they probably have?

( ) Yes
( ) No

Logic: If 3 partners

If you know that two of these partners had sex with each other in the last 6 months, or think they probably have, tick the box that matches both partners' nicknames below.

[ ] [question("option value"), id="403", option="10392"] and [question("option value"), id="403", option="10393"]
[ ] [question("option value"), id="403", option="10392"] and [question("option value"), id="403", option="10394"]
[ ] [question("option value"), id="403", option="10393"] and [question("option value"), id="403", option="10394"]

Thank You!
